# Esthetical and patient‐reported outcomes after root coverage procedures for multiple gingival recessions: A systematic review and meta‐analysis

**DOI:** 10.1111/prd.70050

**Published:** 2026-05-14

**Authors:** Martina Stefanini, Ilham Mounssif, Elena Figuero, Giovanni Zucchelli, Anton Sculean, Raluca Cosgarea

**Affiliations:** ^1^ Periodontal Unit, Department of Biomedical and Neuromotor Sciences University of Bologna Bologna Italy; ^2^ Etiology and Therapy of Periodontal and Peri‐Implant Diseases (ETEP) Research Group, Department of Dental Clinical Specialties, Faculty of Dentistry University Complutense of Madrid Madrid Spain; ^3^ Department of Periodontology, Dental School of Dental Medicine University of Bern Bern Switzerland; ^4^ Department of Periodontology, Operative and Preventive Dentistry University of Bonn Bonn Germany; ^5^ Department of Periodontology and Peri‐Implant Diseases University of Marburg Marburg Germany; ^6^ Faculty of Dentistry Iuliu Hatieganu University Cluj‐Napoca Romania

**Keywords:** esthetics, gingival recession, patient‐reported outcome measures, periodontium, surgical flaps

## Abstract

**Background:**

Multiple gingival recessions can lead to esthetic impairments and may require surgical root coverage. Esthetic outcome measures can be assessed from both professional and patient perspectives. Several objective and subjective outcome measures have been applied for these evaluations.

**Aim:**

To systematically review and conduct a meta‐analysis of the available literature on professional esthetic assessments and patient‐related outcome measures (PROMs) following surgical root coverage procedures for the treatment of multiple gingival recessions.

**Materials and Methods:**

A computerized systematic search was conducted in the MEDLINE (via PubMed), EMBASE, and Cochrane Central Register of Controlled Trials databases up to May 2024 to identify eligible studies meeting the inclusion criteria.

**Results:**

A total of 32 randomized controlled trials involving 1012 patients and 3589 multiple gingival recessions were included. No case‐series studies meeting the inclusion criteria were retrieved. Meta‐analyses demonstrated that root coverage procedures statistically significantly improved both professionally assessed and patient‐reported esthetic outcomes. The overall weighted mean Root Coverage Esthetic Score (RES) was 8.31 (95% CI: 8.11–8.50), with comparable results across coronally advanced flap (CAF) and tunnel (TUN) techniques, particularly when combined with autogenous connective tissue grafts or graft substitutes. Patient‐reported esthetic satisfaction, measured primarily by visual analog scales (VAS), showed a pooled mean of 8.59 (95% CI: 8.29–8.89). Mean root coverage (MRC) reached 82.6% (95% CI: 71.3–93.9), and complete root coverage (CRC) was 62.7% (95% CI: 57.0–68.4). Statistically significant reductions in recession depth (mean difference = 2.22 mm) and gains in keratinized tissue (0.74 mm), gingival thickness (0.56 mm), and clinical attachment level (2.17 mm) were observed. Postoperative pain was low across techniques (VAS 0–10 mean: 2.67; VAS 0–100 mean: 24.34). Metaregression revealed a positive association between MRC and RES (*R* ^2^ = 0.345) but no significant correlation between MRC and patient esthetic perception (*R* ^2^ = 0.091), underscoring the divergence between clinical and patient‐reported outcomes.

**Conclusions:**

The results of this systematic review and meta‐analysis, focusing on multiple gingival recessions, suggest that (a) CAF and TUN with the adjunctive use of autogenous graft support esthetic improvement from both professional and patient perspectives and (b) CAF and TUN with the adjunctive use of autogenous graft or graft substitutes are effective in root coverage outcomes with a minimal postoperative morbidity.

## 1 INTRODUCTION

Presence of gingival recession (GR), characterized by the apical displacement of the gingival margin beyond the cementoenamel junction, and its correlated factors (i.e., esthetic concerns, hypersensitivity, root caries, plaque accumulation) can significantly impair oral health and diminish the overall quality of life^1^, ^2^. It represents a common mucogingival condition impacting over 75% of the global population,^3^ and its occurrence may be attributed to various factors, including periodontal disease, anatomical predisposition, incorrect toothbrushing, inadequate oral hygiene, traumatic malocclusion, or iatrogenic causes.^4^, ^5^, ^6^, ^7^ Gingival recession (RT1/RT2) can be limited to one tooth or affect several adjacent teeth (multiple gingival recessions), presenting significant concerns for periodontal health and esthetics.^8^, ^9^, ^10^, ^11^ These defects are more challenging to treat than a single gingival recession, as the surgical field is larger, and more anatomical variations may be present (such as prominent roots, shallow vestibules, or defect size).^12^

While root coverage procedures for single defects are well‐established and highly predictable, as supported by robust evidence in the literature,^13^, ^14^, ^15^ the optimal approach for multiple defects remains uncertain.^12^, ^16^ Techniques such as the coronally advanced flap, with or without adjunctive materials (e.g., autogenous grafts, graft substitutes, enamel matrix derivatives), have demonstrated consistent success in single recessions. However, data on their effectiveness for multiple gingival recessions remains scarce. Notably, in the systematic review by Chambrone et al., only five studies addressed multiple defects.^16^ Despite this limited evidence, the authors drew a significant conclusion that root coverage procedures can be beneficial for multiple gingival recessions, similar to their effectiveness in single defects.

From a professional perspective, the esthetic goal of surgical gingival recession coverage is achieving 100% root coverage (i.e., up to the cementoenamel junction), with soft tissue that exhibits optimal texture and color integration. This should occur without visible scarring and ensure continuity and blending with the surrounding anatomical structures (e.g., mucogingival line, gingival margin, and presence of keratinized tissue). However, the professional success assessment must be complemented by the patient's satisfaction.

Until recently, surrogate endpoints such as mean root coverage, complete root coverage, and recession reduction were the primary parameters used to assess surgical outcomes,^17^, ^18^, ^19^ often supplemented by anecdotal methods for evaluating esthetic results. However, more standardized approaches (such as the Visual Analog Scale (VAS))^20^, ^21^, ^22^, ^23^, ^24^ and especially the Root Coverage Esthetic Score (RES)^25^ have been introduced to enhance consistency in outcome assessment. Especially RES is a reliable tool for assessing the esthetic outcomes after root coverage procedures for operators with different experience levels,^26^, ^27^ playing a crucial role in this, providing a structured and reproducible method for scoring esthetic outcomes. These tools facilitate clearer communication between clinicians and enable more reliable comparisons across clinical trials.

From the patient's perspective, there is increasing recognition of the importance of patient‐reported outcomes (PROMs) in evaluating the success of root coverage procedures.^17^, ^18^, ^19^ While esthetics often serve as the primary motivation for undergoing such procedures, traditional evaluations have primarily relied on professional assessments, with limited incorporation of patient perspectives.^18^, ^19^ However, the role of patient‐reported outcomes in enhancing patient‐centered care in periodontology is of utmost significance, as it increasingly emphasizes the need to integrate subjective experiences into clinical decision‐making and treatment evaluation.

Beyond esthetic considerations, a more comprehensive assessment of PROMs is now widely acknowledged as essential for understanding the true impact of root coverage procedures on patients' well‐being. Factors such as postoperative morbidity, pain, discomfort, and healing experiences significantly influence patient satisfaction and should be considered alongside clinical parameters. Furthermore, oral function, including speech, mastication, and overall comfort, plays a crucial role in daily life and can be affected by both the condition and its treatment. Psychological well‐being is another critical dimension, as concerns about gingival recession often extend beyond esthetics to include self‐esteem, social confidence, and anxiety about oral health. The use of a comprehensive range of patient‐reported outcome measures (PROMs) is crucial in enabling a more patient‐centered evaluation of treatment success, ensuring that therapeutic approaches align with both clinical effectiveness and patient satisfaction.

While professional outcomes in root coverage procedures have significantly advanced in recent years, gaining valuable recognition within the scientific community, including indices such as the RES, there remain substantial challenges in effectively assessing outcomes from the patient's perspective.

The present systematic review, conducted in the context of the European Federation of Periodontology (EFP) Focused Workshop on Esthetics and Patient‐Reported Outcomes in Periodontology and Implant Dentistry, aims to evaluate the effect of root coverage therapy for the treatment of multiple gingival recessions in terms of professional esthetic and patient‐reported outcome measures (PROMs).

The following focused question was addressed: “In patients with a clinical diagnosis of multiple RT1/2 GR (MGR), what is the impact of root coverage surgical therapy in terms of esthetic and patient‐reported outcome measures?”

## 2 MATERIALS AND METHODS

### 2.1 Study registration and reporting format

The review protocol has been registered and assigned the ID number CRD42024549350 in the PROSPERO International prospective Register of Systematic Reviews, hosted by the National Institute for Health Research, University of York, Centre for Reviews and Dissemination. A detailed protocol was designed according to the PRISMA statement,^28^ Cochrane Handbook of Systematic Reviews,^29^ and instructions from the Scientific Committee of the EFP Focused Workshop on Esthetics and Patient‐Reported Outcomes in Periodontology and Implant Dentistry.

### 2.2 Focused questions

The goal of conducting this systematic review (SR) was to answer the following question: “In individuals with a clinical diagnosis of multiple RT1/2 GR, what is the impact of root coverage surgical therapy in terms of esthetic and patient‐reported outcome measures?”

### 2.3 Patient, intervention, comparison, outcome, and time (PICOST) question

The following Population, Intervention, Comparison, Outcomes, Study design, and Timeframe (PICOST) were used to guide the inclusion and exclusion of studies for the above‐mentioned focused question:

- Population: Studies including individuals with a clinical diagnosis of multiple gingival recessions in any jaw, categorized as Miller class 1, 2, or 3, or RT 1 or 2.
- Intervention: Studies including any surgical root coverage procedure
- Comparison: Studies including any possible comparisons between surgical interventions for root coverage.
- O: Primary outcomes: Professionally determined esthetic scores and patient esthetic perception and satisfaction (PROMs).

Secondary outcome measures: root sensitivity, postoperative morbidity and complications, mean and complete root coverage, residual recession, keratinized tissue gain, gingival thickness, and clinical attachment level (CAL).

- S: Randomized controlled trials (RCTs) and case series with at least 10 patients per arm.
- T: Studies with a minimum follow‐up of 6 months.

### 2.4 Eligibility criteria

#### 2.4.1 Criteria for inclusion

- Participants ≥18 years old, systemically healthy patients with two or more adjacent gingival recession defects (categorized as Miller class 1, 2, or 3, or RT 1 or 2) affecting either maxillary, mandibular arches, or both.
- Surgical treatments for root coverage involving coronally advanced flap (CAF), tunnel techniques (TUN), free gingival graft (FGG), VISTA or Pinhole surgical technique (PST) in combination with an Adjunctive [autogenous grafts (ATG) or graft substitutes (GS), collagen matrices or acellular dermal matrices (ADMs) and/or Biologic Agents (BA) (i.e., enamel matrix derivative, platelet rich fibrin (PRF), Chorion membrane, hyaluronic acid, Grow factors, recombinant human factor)]. Studies reporting technical variations of the same type of intervention (e.g., flap without releasing incisions versus flap with vertical releasing incisions, etc.) were included and categorized in the review.
- Professionally determined esthetic scores (measured through VAS, RES, or other scales (numerical, Likert)) and self‐reported patient esthetic satisfaction (PROMs measured through VAS or other scales (numerical, Likert)).
- Studies with root coverage (mean root coverage‐MRC, complete root coverage‐CRC) purposes
- Randomized Controlled Trials (RCTs), noncontrolled studies, case series with at least 10 patients of minimum follow‐up of 6 months.

#### 2.4.2 Criteria for exclusion

- Studies that combined the analysis of single and multiple gingival recessions were not eligible.
- Studies published in languages other than English were excluded due to time constraints.

### 2.5 Search strategy

See Appendix S1.

### 2.6 Study selection

See Appendix S1.

### 2.7 Data extraction and management

Mean values for each outcome were extracted based on the reported unit level in each individual study (See Appendix S1).

### 2.8 Quality and risk of bias assessment

See Appendix S1.

### 2.9 Data analyses

To summarize and compare studies, mean changes between final and baseline visits and standard errors (SE) values were directly pooled and analyzed with weighted mean effects (WME) and 95% confidence intervals (CIs). When the differences between (∆) baseline‐end were not reported, they were calculated using the formula: ∆Var = Var2 – Var1, where, Var1 was the mean value before treatment and Var2 the mean value after treatment. In addition, the variance of ∆Var was estimated with the formula: SVar^2^ = SVar1^2^ + SVar2^2^−(2*r*SVar1*SVar2), where SVar^2^ was the variance of the difference, SVar1^2^ is the variance of the mean baseline value, Svar2^2^ is the variance of the mean end value. A correlation *r* of 0.5 was assumed as described before.^30^

In order to compare the changes in study outcomes between baseline and final visits, all study designs were included, but considering each arm of RCTs or controlled clinical trials (CCTs) as an independent study.^31^, ^32^ Subgroup analyses were performed based on the type of technique and on the presence of any adjunctives. Comparisons between interventions were restricted to subgroup analyses. A random‐effects approach with the DerSimonian & Laird method for between‐study variance was chosen a priori for pooling individual study effects.^33^ The statistical absolute and relative between‐trial heterogeneity was assessed using the Tau^2^ and the I2 index, respectively; the latter is defined as the percentage of variation in the global estimate that was attributable to heterogeneity (I^2^ = 25%: low; I^2^ = 50%: moderate; I^2^ = 75%: high heterogeneity). Forest plots were created to illustrate the effects of the meta‐analysis on the global estimation and the various subanalyses. Fixed‐effect metaregressions were performed to determine the association between MRC and patient or professional esthetic evaluation (eprf_res), as well as between patient (eptf_vas) and professional (eprf_res) esthetic evaluations.

STATA®14 (StataCorp LP, Lakeway Drive, College Station, Texas, USA) intercooled software was used to conduct all analyses. Statistical significance was set at *p* < 0.05.

## 3 RESULTS

### 3.1 Search results and study selection

Following the removal of duplicates, 835 records were screened based on titles and abstracts, and a full‐text assessment was performed for 64 articles. Inter‐examiner reliability was high in both the title and abstract (κ score = 0.83, 95% CI: 0.79–0.87) and the full‐text (κ score = 0.91; 95% CI, 0.88–0.94) selection. Based on the predetermined inclusion criteria, 35 publications pertaining to 32 randomized clinical trials (24 parallel design and 11 split‐mouth design) were ultimately included in this SR. Only 20 parallel and 10 split‐mouth design studies were included in the meta‐analysis of the primary outcomes due to a lack of data.

Publications by Rotundo et al. were related to the same population study evaluating only patient esthetic satisfaction in the first one (2019) and professional outcomes in the second paper (2021) in the same follow‐up of 12 months.^34^, ^35^ Moreover, publications by Tonetti et al., Pelekos et al., and Tonetti et al. used the same cohort baseline population to evaluate Proms in the first (2018) and only professional esthetic outcomes in the second paper by Pelekos et al.^36^, ^37^, ^38^ Tonetti et al. reported long‐term (36 months) clinical outcomes of the same patient evaluated in manuscript from 2018 publication^37^, ^38^ and could not be included in the meta‐analyses, as the significant loss of patients during follow‐up relative to the baseline cohort would have compromised the consistency of the analysis.

**FIGURE 1:**
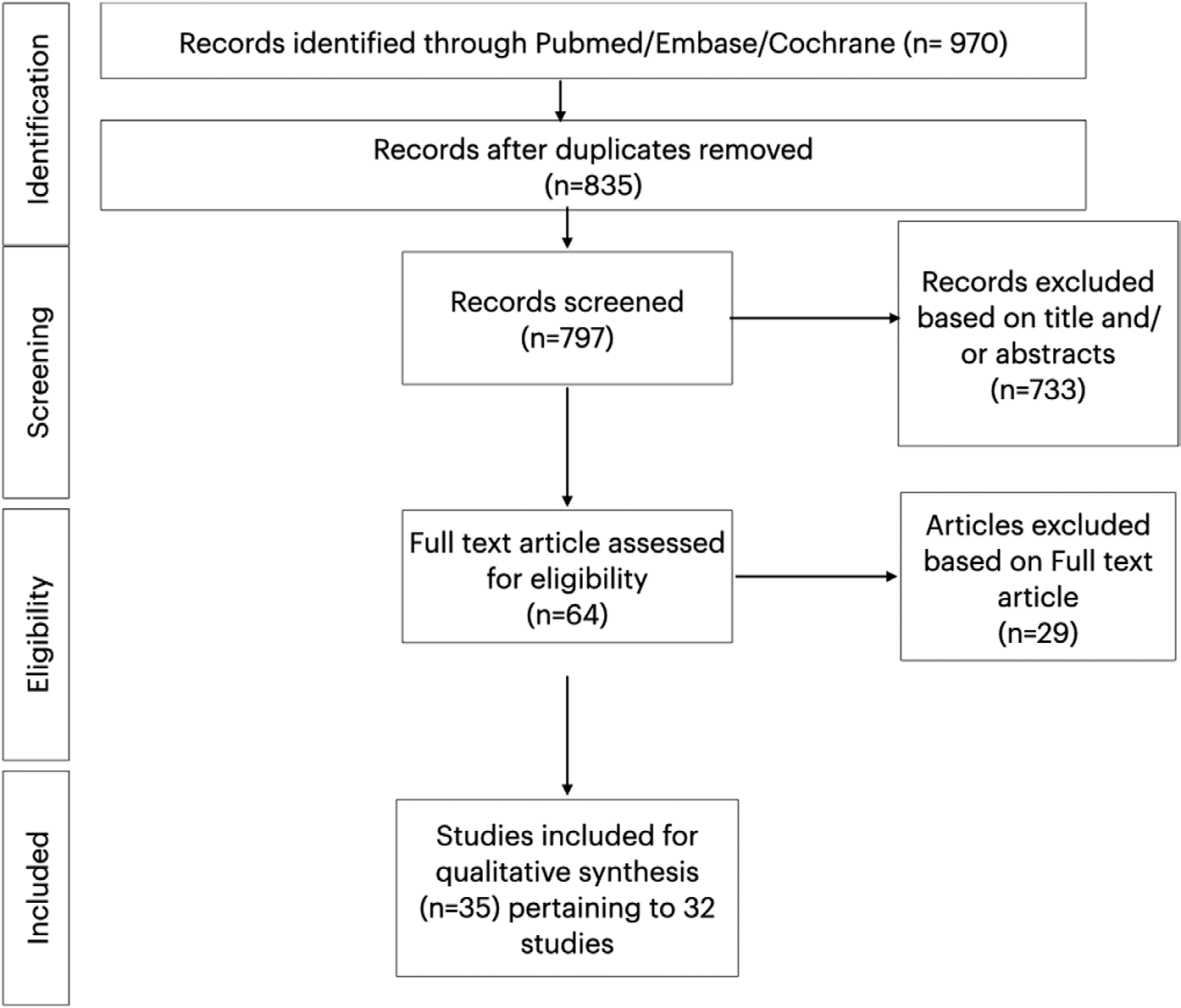
PRISMA flow chart displaying the performed search strategy leading to the inclusion of 35 papers.

Figure 1 presents a flowchart that outlines the article selection process. No case‐series studies meeting the inclusion criteria were retrieved.

The reason for the exclusion of the other 29 articles is presented in the Table S1.

### 3.2 Risk of bias assessment

**FIGURE 2:**
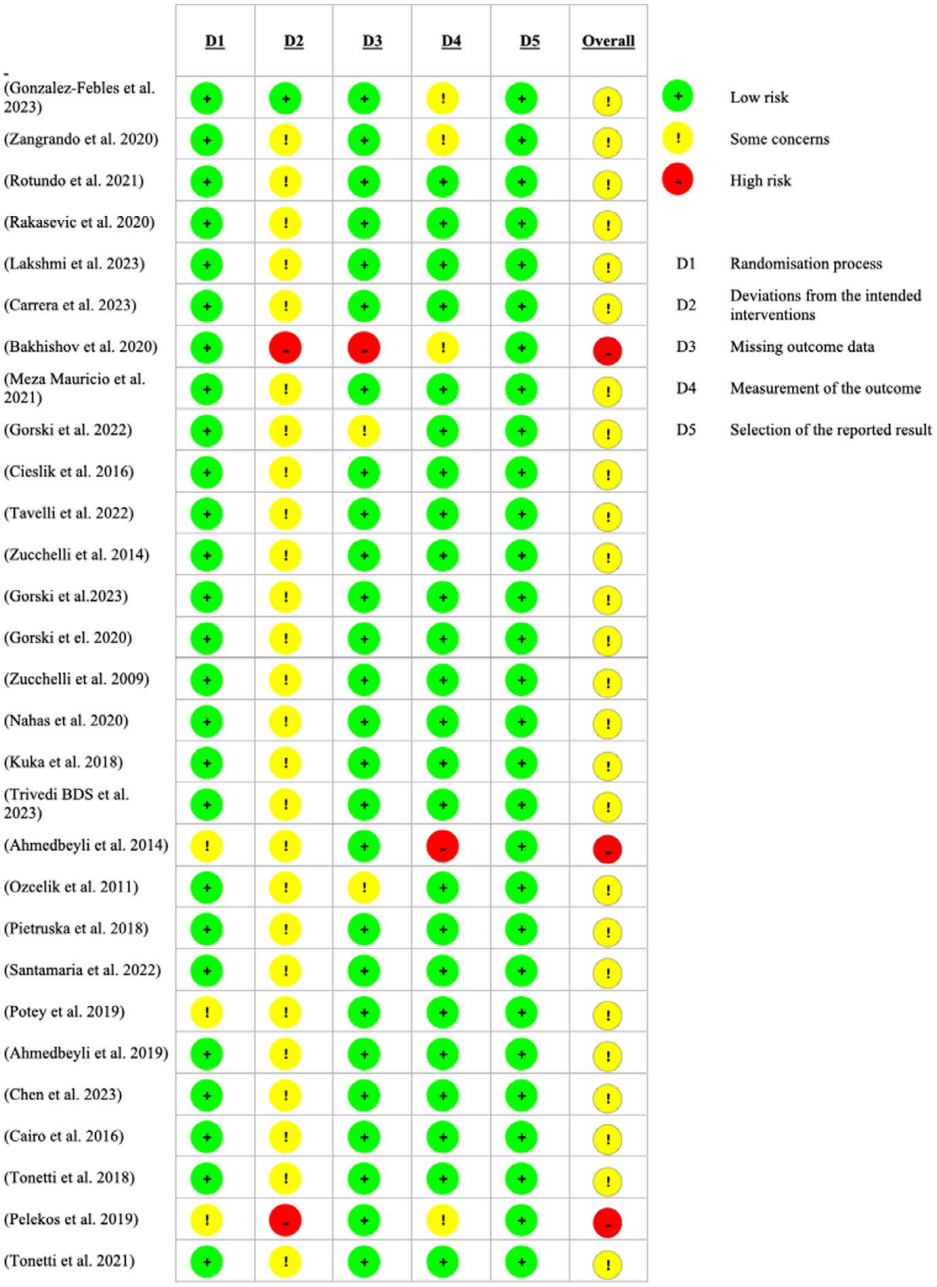
Risk of bias assessment for the included RCTs for Professionally determined Esthetic Outcome.

**FIGURE 3:**
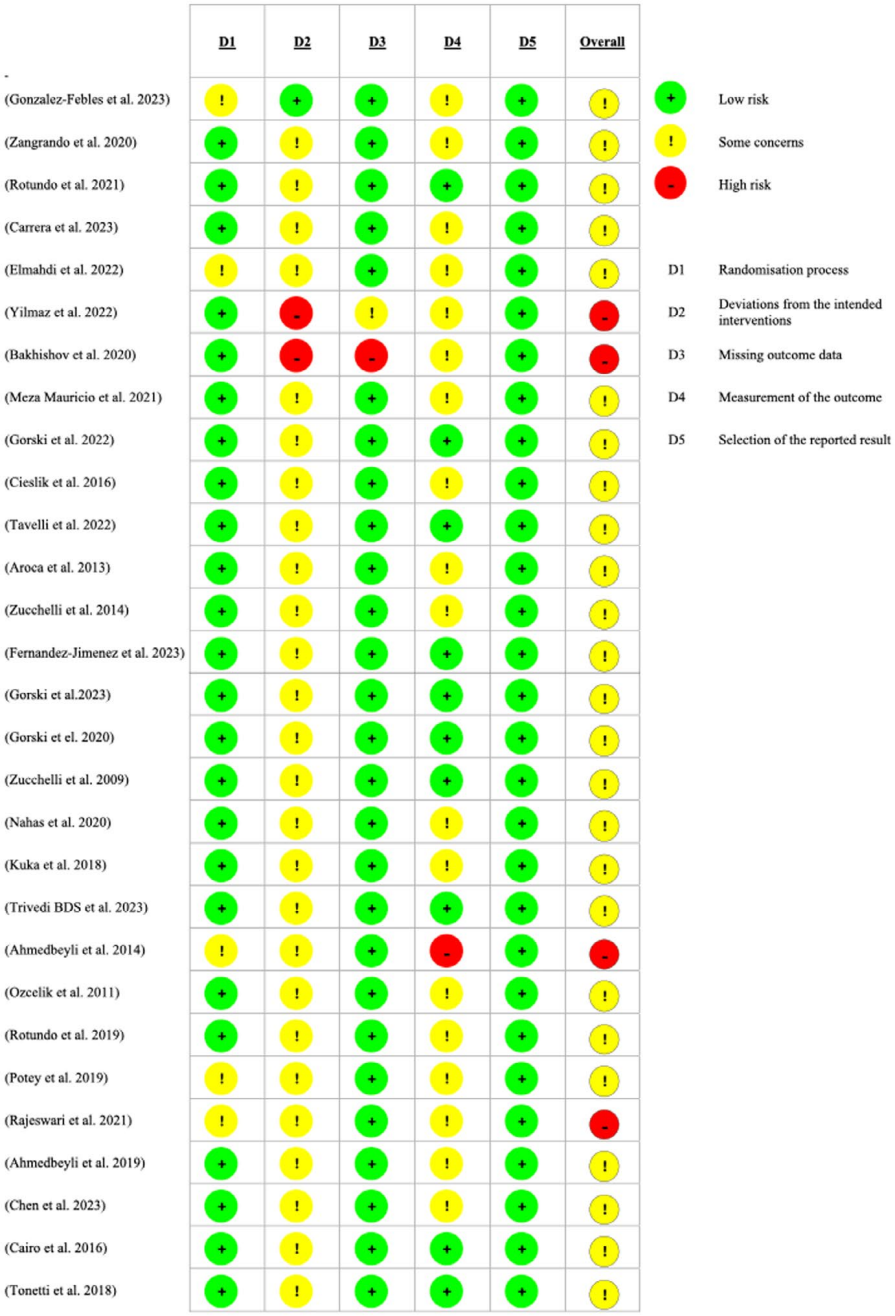
Risk of bias assessment for the included Patient‐determined Esthetic Outcomes.

Quality assessment of the included studies, evaluated for each outcome, is summarized in Figures 2 and 3.

Concerning the Professionally Determined Esthetic Scores, the overall assessment of the risk of bias raised some concerns regarding the totality of the included studies. Three papers were found to have a high risk of bias due to significant issues related to analyses that excluded trial participants postrandomization, missing outcome data (domain 3), and problems concerning the implementation of the intervention (domain 2), which could potentially influence the outcome.^36^, ^39^, ^40^

Although all reported results for the outcome measures were aligned with the intended analyses, Domain 2 was consistently rated across the individual assessments at moderate risk of bias when intention‐to‐treat (ITT) analyses were not employed or adequately reported.

Considering PROMs, none of the included studies were rated as having an overall low risk of bias.

Four studies were identified as having a high risk of bias due to issues related to the measurement of outcomes and concerns about deviations from the intended intervention.^39^, ^40^, ^41^, ^42^

A detailed report on the quality of the included studies is provided in Table S2–S5, categorized by outcome and domains.

### 3.3 Study description

This SR incorporated 32 RCTs, which involved 1012 patients and treated 3589 multiple gingival recession defects using various surgical techniques. CAF (including envelope design or vertical releasing incisions) was implemented in 18 studies.^21^, ^23^, ^35^, ^38^, ^39^, ^41^, ^43^, ^44^, ^45^, ^46^, ^47^, ^48^, ^49^, ^50^, ^51^, ^52^, ^53^, ^54^ CAF alone was assessed in 7 studies.^21^, ^23^, ^34^, ^35^, ^39^, ^49^, ^54^ CAF was combined with an ATG in 9 RCTs,^23^, ^38^, ^44^, ^45^, ^47^, ^48^, ^51^, ^53^, ^54^ a GS in 8 studies,^35^, ^38^, ^39^, ^43^, ^47^, ^48^, ^51^, ^52^ and a BA in 2 trials.^41^, ^46^ Additionally, CAF with the adjunctive use of GS combined with BA,^52^ or OB,^49^ or both OB + BA^50^ was reported.

The tunnel technique (TUN) with the adjunctive use of an ATG was applied in 14 studies,^40^, ^42^, ^45^, ^55^, ^56^, ^57^, ^58^, ^59^, ^60^, ^61^, ^62^, ^63^, ^64^, ^65^ or in combination with a GS in 6 trials,^55^, ^58^, ^59^, ^63^, ^64^, ^65^ a BA in one study,^56^ and in conjunction with ATG + BA^60^, ^61^, ^62^ or ATG + EDTA.^62^ Vista technique was implemented in three studies,^41^, ^44^, ^57^ PST^66^ and FGG^42^ in one trial.

The follow‐up duration varied from 6 to 12 months for most publications; only four papers reported longer evaluation periods: 16 months,^56^ 18 months,^41^ 3 years,^37^ and 5 years.^23^

Primary data on age and gender distribution, smoking status, MGR defect type, duration of surgery, painkillers, and antibiotics consumption are available in the data collection form under Table S6.

### 3.4 Study outcomes

Tables 1 and 2 display the primary and secondary outcomes of the included studies. Patient was used as the unit of analysis in 6 studies, while defect was used in 23 studies when professional outcomes were evaluated. Defect‐level values could not be aggregated to the patient level since individual patient data could not be retrieved from Authors.

**TABLE 1:** Descriptive data on primary outcomes of the included studies.

id	Study	Technique	Adjunctive	Professional esthetic evaluation					Patient esthetic evaluation				Method of assestment
				Unit of evaluation	Number	Mean	SD	Method of assestment	Unit of evaluation	Number	Mean	SD	
1	Gonzalez‐Febles 2023_CAF_ATG^45^	CAF	ATG	Recession	42	8.8	1.8	RES	Patient	14	2.2	1.2	5‐point Likert scale
1	Gonzalez‐Febles 2023_TUN_ATG^45^	TUN	ATG	Recession	41	8.8	1.5	RES	Patient	15	2	1.1	5‐point Likert scale
2	Zangrando 2020_CAF_ATG^53^	CAF	ATG	Patient	21	8.04	1.18	RES	Patient	21	8.48	1.72	VAS
2	Zangrando 2020_CAF_ATG^53^	CAF	ATG	Patient	21	8.36	1.17	RES	Patient	21	8.57	1.57	VAS
3&4	Rotundo 2019_2021_CAF_NO_ADG^34^	CAF	NO_ADJ	Recession	31	0.3	0.5	SEI	Patient	12	8.8	2.0	VAS
3&4	Rotundo 2019_2021_CAF_GS^34^	CAF	GS	Recession	31	0.7	0.5	SEI	Patient	12	9.3	1.0	VAS
5	Rakasevic 2020_TUN_GS^65^	TUN	GS	Recession	52	8.96	1.45	RES	na	na	na	na	na
5	Rakasevic 2020_TUN_ATG^65^	TUN	ATG	Recession	62	8.52	1.49	RES	na	na	na	na	na
6	Lakshmi 2023_TUN_ATG^63^	TUN	ATG	Recession	31	6.36	1.81	RES	na	na	na	na	na
6	Lakshmi 2023_TUN_GS^63^	TUN	GS	Recession	33	8.19	1.7	RES	na	na	na	na	na
7	Carrera 2023_TUN_ATG^56^	TUN	ATG	Recession	36	7.0	6.12	RES	Patient	14	9.65	na	VAS
7	Carrera 2023_TUN_BA^56^	TUN	BA	Recession	36	7.0	8.42	RES	Patient	14	8.60	na	VAS
8	Elmahdi 2022_TUN_GS^59^	TUN	GS	na	na	na	na	na	Patient	12	8.24	0.65	VAS
8	Elmahdi 2022_TUN_ATG^59^	TUN	ATG	na	na	na	na	na	Patient	12	8.24	0.43	VAS
10	Ylmaz 2022_FGG_NO_ADJ^42^	FGG	NO_ADJ	na	na	na	na	na	Patient	20	0.65	0.68	4‐point verbal scale
10	Ylmaz 2022_TUN_ATG^42^	TUN	ATG	na	na	na	na	na	Patient	18	1.62	0.56	
11	Bakhishov 2021_TUN_ATG^40^	TUN	ATG	Recession	31	8.58	1.85	RES	na	na	na	na	na
11	Bakhishov 2021_TUN_ATG^40^	TUN	ATG	Recession	30	8.76	1.52	RES	na	na	na	na	na
12	Meza‐Mauricio 2021_CAF_GS^47^	CAF	GS	Recession	66	8.31	0.2	RES	na	na	na	na	na
12	Meza‐Mauricio 2021_CAF_ATG^47^	CAF	ATG	Recession	64	8.12	1.03	RES	na	na	na	na	na
13	Gorski 2022_TUN_ATG^62^	TUN	ATG	Recession	67	8.79	1.31	RES	Patient	19	8.47	1.39	VAS
13	Gorski 2022_TUN_ATG_EDTA^62^	TUN	ATG_EDTA	Recession	69	8.98	1.3	RES	Patient	19	7.8	2.3	VAS
14	Tavelli 2022_CAF_GS^67^	CAF	GS	Recession	44	6.98	1.41	RES	Patient	15	8.91	1.22	VAS
14	Tavelli 2022_CAF_GS_BA^67^	CAF	GS_BA	Recession	47	8.17	1.99	RES	Patient	15	9.0	1.14	VAS
15	Aroca 2013_TUN_GS^55^	TUN	GS	na	na	na	na	na	Patient	22	9.06	0.8	VAS
15	Aroca 2013_TUN_ATG^55^	TUN	ATG	na	na	na	na	na	Patient	22	9.29	0.8	VAS
16	Zucchelli 2014_CAF_NO_ADJ^23^	CAF	NO_ADG	Patient	25	9.08 8.76	NA	COLOR VAS CONTOUR VAS	Patient	25	8.84	na	VAS
16	Zucchelli 2014_CAF_ATG^23^	CAF	ATG	Patient	25	7.84 8.96	NA	COLOR VAS CONTOUR VAS	Patient	25	8.44	na	VAS
17	Fernández‐Jiménez 2023_CAF_ATG^44^	CAF	ATG	na	na	na	na	na	Patient	12	8.07		VAS
17	Fernández‐Jiménez 2023_VISTA_ATG^44^	VISTA	ATG	na	na	na	na	na	Patient	10	8.38		VAS
18	Gorski 2023_TUN_ATG^61^	TUN	ATG	Recession	133	9.04	1.31	RES	Patient	24	8.54	1.05	VAS
18	Gorski 2023_TUN_ATG_BA^61^	TUN	ATG_BA	Recession	133	9.47	1.00	RES	Patient	24	8.74	0.88	VAS
19	Gorski 2020_TUN_ATG_BA^60^	TUN	ATG_BA	Recession	75	8.71	1.37	RES	Patient	20	8.15	1.35	VAS
19	Gorski 2020_TUN_ATG^60^	TUN	ATG	Recession	75	9.25	1.27	RES	Patient	20	8.3	1.25	VAS
20	Zucchelli 2009_CAF_NO_ADG^21^	CAF	NO_ADJ	Patient	16	8.7 7.0	1.4 1.96	COLOR VAS CONTOUR VAS	na	na	na	na	na
20	Zucchelli 2009_CAF_NO_ADG^21^	CAF	NO_ADJ	Patient	16	9.5	0.89	COLOR VAS CONTOUR VAS	na	na	na	na	na
21	Cieślik‐Wegemund 2016_TUN_GS^58^	TUN	GS	Patient	14	8.77 8.27	1.8 2.88	COLOR VAS CONTOUR VAS	na	na	na	na	na
21	Cieślik‐Wegemund 2016_TUN_ATG^58^	TUN	ATG	Patient	14	9.88 8.83	0.2 0.5	COLOR VAS CONTOUR VAS	na	na	na	na	na
22	Nahas 2020_CAF_ATG^48^	CAF	ATG	Recession	40	8.94	na	VAS	Patient	15	9.57	NA	VAS
22	Nahas 2020_CAF_GS^48^	CAF	GS	Recession	42	8.40	na	VAS	Patient	15	9.23	NA	VAS
23	Kuka 2018_CAF_NO_ADG^46^	CAF	NO_ADJ	Patient	12	7.0	0.00	RES	na	na	na	na	na
23	Kuka 2018_CAF_BA^46^	CAF	BA	Patient	12	7.8	1.32	RES	na	na	na	na	na
24	Trivedi 2024_PST_NO_ADG^66^	PST	NO_ADJ	Recession	81	8.7	1.53	RES	na	na	na	na	na
24	Trivedi 2024_PST_BA^66^	PST	BA	Recession	84	9.38	1.23	RES	na	na	na	na	na
25	Ahmedbeyli 2014_CAF_NO_ADG^39^	CAF	NO_ADJ	Patient	12	7.58	2.02	RES	na	na	na	na	na
25	Ahmedbeyli 2014_CAF_GS^39^	CAF	GS	Patient	12	9.08	1.5	RES	na	na	na	na	na
26	Ozcelik 2011_CAF_NO_ADG^49^	CAF	NO_ADJ	Recession	77	7.43	1.56	RES	Patient	77	7.15	1.18	VAS
26	Ozcelik 2011_CAF_OB^49^	CAF	OB	Recession	78	8.65	1.47	RES	Patient	78	8.18	0.73	VAS
27	Pietruska 2018_TUN_GS^64^	TUN	GS	Recession	45	8.36	1.78	RES	na	na	na	na	na
27	Pietruska 2018_TUN_ATG^64^	TUN	ATG	Recession	46	7.11	1.95	RES	na	na	na	na	na
28	Santamaria 2022_CAF_GS^51^	CAF	GS	Recession	39	6.4	2.4	RES	Patient	19	9.5	0.6	VAS
28	Santamaria 2022_CAF_ATG^51^	CAF	ATG	Recession	39	6.8	2.3	RES	Patient	19	9.7	0.5	VAS
29	Potey 2019_CAF_OB^50^	CAF	OB	Recession	75	8.69	1.46	RES	Patient	10	7.25	2.55	VAS
29	Potey 2019_CAF_OB_BA^50^	CAF	OB_BA	Recession	75	8.84	1.41	RES	Patient	10	7.5	2.56	VAS
30	Rajeswari 2021_CAF_BA^41^	CAF	BA	na	na	na	na	na	Patient	16	6.6	0.72	VAS
30	Rajeswari 2021_VISTA_BA^41^	VISTA	BA	na	na	na	na	na	Patient	16	9.1	0.81	VAS
31	Ahmedbeyli 2019_CAF_GS^43^	CAF	GS	Recession	11	8.91	1.97	RES	na	na	na	na	na
31	Ahmedbeyli 2019_CAF_GS^43^	CAF	GS	Recession	11	9.45	1.21	RES	na	na	na	na	na
32	Chen 2023_VISTA_ATG^57^	VISTA	ATG	Recession	28	8.82	1.44	RES	Patient	19	9.5	0.6	VAS
32	Chen 2023_TUN_ATG^57^	TUN	ATG	Recession	31	8.52	1.46	RES	Patient	19	9.7	0.5	VAS
33	Cairo 2016_CAF_NO_ADG^54^	CAF	NO_ADG	Recession	38	7.9	1.4	RES	Patient	16	8.66	1.31	VAS
33	Cairo 2016_CAF_ATG^54^	CAF	ATG	Recession	36	7.9	1.4	RES	Patient	16	9.35	0.6	VAS
34&35	Tonetti 2018_Pelekos 2019_CAF_ATG^36^, ^38^	CAF	ATG	Recession	207	*7.9*	2.4	RES	Patient	95.0	1.3	1.3	Final 5‐point Likert scale
34&35	Tonetti 2018_Pelekos 2019_CAF_GS^36^, ^38^	CAF	GS	Recession	186	6.4	3.7	RES	Patient	92.0	1.5	1.3	

Abbreviations: ADM, allodermic membrane; ATG, autogenous graft (any type of technique); BA, biologic agents (enamel matrix derivative, platelet rich fibrin, chorion membrane, hyaluronic acid, growth factors, recombinant human factors); CAF, coronally advanced flap; CM, collagen membrane; FGG, free gingival graft; OB, orthodontic application; PST, Pinhole surgical technique; TUN, tunnel; VISTA, Vista.

**TABLE 2:** Descriptive data on relevant secondary outcomes of the included studies.

Id	Study	Technique	Adjunctive	MRC mean (%)	MRC SD	CRC (%)	Rec Red mean (mm)	Rec Red SD (mm)	KT change mean (mm)	KT change SD	GT change mean (mm)	GT change SD	CAL change mean (mm)	CAL change SD	Pain VAS (0–10) mean	Pain VAS (0–10) SD	Pain VAS (0–100) mean	Pain VAS (0–100) SD
1	Gonzalez‐Febles 2023_CAF_ATG^45^	CAF	ATG	91.10	18.8	78.1	2.7	1.00	na	na	na	na	na	na			32.60	27.30
1	Gonzalez‐Febles 2023_TUN_ATG^45^	TUN	ATG	94.00	14.1	80.9	2.6	2.50	na	na	na	na	1.98	1.29			21.80	21.20
2	Zangrando 2020_CAF_ATG^53^	CAF	ATG	78.00	22.22	38	2.7	0.83	0.84	1.03	0.6	0.4	1.61	1.15	4.66	2.31		
2	Zangrando 2020_CAF_ATG^53^	CAF	ATG	82.50	21.8	38	2.09	0.72	0.62	1.41	0.6	0.5	2.20	1.50	7.86	1.24		
3, 4	Rotundo 2019_2021_CAF_NO_ADG^34^	CAF	NO_ADJ	75.00	30.00	52	2.00	1.10	−1.1	1.30	−0.3	0.7	2.10	0.80	1.20	2.20		
3, 4	Rotundo 2019_2021_CAF_GS^34^	CAF	GS	87.00	19.00	63	2.00	1.80	−1.6	0.60	0.2	0.7	3.17	1.25	0.70	0.90		
5	Rakasevic 2020_TUN_GS^65^	TUN	GS	85.25	14.9	46.8	2.31	0.93	0.85	1.20	0.8	0.3	2.98	1.40				
5	Rakasevic 2020_TUN_ATG^65^	TUN	ATG	87.6	15.1	51.9	2.26	1.17	0.84	1.00	0.7	0.3	2.89	0.62				
6	Lakshmi 2023_TUN_ATG^63^	TUN	ATG	56.8	23.9	9.1	1.52	0.66	0.58	0.66	0.6	0.1	3.05	0.66	6.58	0.50		
6	Lakshmi 2023_TUN_GS^63^	TUN	GS	79.03	20.16	45.16	2.00	0.50	1.29	0.51	0.8	0.1	0.77	0.92	2.06	0.25		
7	Carrera 2023_TUN_ATG^56^	TUN	ATG	55.42	37.14	55.55	1.06	0.82	−0.52	1.04	0.2	0.6	0.36	0.96			3.90	6.14
7	Carrera 2023_TUN_BA^56^	TUN	BA	29.53	0.8	24.32	0.40	0.75	−1.90	1.41	−0.20	0.5	2.46	1.94			2.40	2.91
8	Elmahdi 2022_TUN_GS^59^	TUN	GS	72.72	23.36	na	2.1	0.64	0.21	0.84	0.5	0.4	2.47	1.28	0.12			
8	Elmahdi 2022_TUN_ATG^59^	TUN	ATG	82.62	16.3	na	2.23	0.68	1.15	1.16	0.9	0.5	2.05	1.16	2.00			
10	Ylmaz 2022_FGG_NO_ADJ^42^	FGG	NO_ADJ	63.54	26.69	20	2.29	0.92	4.43	1.34	0.7	0.2	2.95	0.51				
10	Ylmaz 2022_TUN_ATG^42^	TUN	ATG	85.82	12.74	35	2.75	0.41	1.95	1.06	1.4	0.3	2.07	1.22				
11	Bakhishov 2021_TUN_ATG^40^	TUN	ATG	83.16	23.32	61.3	2.31	1.10	1.71	1.10	0.8	0.3	2.01	0.83	0.8	1.72		
11	Bakhishov 2021_TUN_ATG^40^	TUN	ATG	91.72	16.59	76.7	2.36	0.78	1.50	1.18	0.9	0.5	1.39	0.98	0.6	1.00		
12	Meza‐Mauricio 2021_CAF_GS^47^	CAF	GS	80.19	16.3	73.3	2.39	0.12	0.63	0.83	na	na	1.67	1.25				
12	Meza‐Mauricio 2021_CAF_ATG^47^	CAF	ATG	91.79	10.1	83.3	2.75	0.11	0.99	1.23	na	na	1.27	1.14				
13	Gorski 2021_TUN_ATG^62^	TUN	ATG	84.69	30.82	91.4	1.51	1.35	0.79	1.00	0.5	0.5	1.34	1.10			4.75	7.15
13	Gorski 2021_TUN_ATG_EDTA^62^	TUN	ATG_EDTA	86.08	31.76	90.2	1.56	1.18	0.78	1.18	0.6	0.5	na	na			4.50	7.59
14	Tavelli 2022_CAF_GS^52^	CAF	GS	77.72	14.4	20.45	2.3	1.05	0.25	1.08	0.5	0.3	na	na				
14	Tavelli 2022_CAF_GS_BA^52^	CAF	GS_BA	88.25	16.31	59.57	2.54	0.68	0.32	0.84	0.8	0.4	1.30	0.60				
15	Aroca 2013_TUN_GS^55^	TUN	GS	71.00	20.00	42	1.30	0.56	0.30	0.82	0.2	0.3	1.70	0.46				
15	Aroca 2013_TUN_ATG^55^	TUN	ATG	90.00	18.00	85	1.60	0.44	0.70	0.75	0.5	0.4	3.05	0.79				
16	Zucchelli 2014_CAF_NO_ADJ^23^	CAF	NO_ADG	na	na	89.04	2.95	0.81	0.65	0.50	na	na	3.01	0.83				
16	Zucchelli 2014_CAF_ATG^23^	CAF	ATG	na	na	86.84	3.02	0.84	1.00	0.51	na	na	1.75	1.16				
17	Fernández‐Jiménez 2023_CAF_ATG^44^	CAF	ATG	56.49	29.27	27.5	1.78	1.10	0.58	1.10	na	na	2.40	1.43				
17	Fernández‐Jiménez 2023_VISTA_ATG^44^	VISTA	ATG	73.26	23.64	47.2	1.98	0.77	0.88	0.84	na	na	2.18	1.07				
18	Gorski 2023_TUN_ATG^61^	TUN	ATG	83.00	35.00	93	1.59	1.15	0.65	1.35	0.8	1.0	2.21	1.12	1.50	2.14		
18	Gorski 2023_TUN_ATG_BA^61^	TUN	ATG_BA	85.00	34.00	91	1.66	1.06	0.67	1.33	1.0	0.1	2.04	1.40	1.60	2.12		
19	Gorski 2020_TUN_ATG_BA^60^	TUN	ATG_BA	87.49	29.43	86.7	1.91	1.14	0.94	1.12	0.6	0.5	1.79	1.59	1.70	0.67		
19	Gorski 2020_TUN_ATG^60^	TUN	ATG	90.93	23.2	85.3	2.08	1.50	1.02	1.27	0.7	0.7	2.28	0.72	2.13	1.31		
20	Zucchelli 2009_CAF_NO_ADG^21^	CAF	NO_ADJ	92.64	14.25	77.7	2.33	0.85	0.44	0.50	na	na	2.57	0.92				
20	Zucchelli 2009_CAF_NO_ADG^21^	CAF	NO_ADJ	97.27	8.08	89.3	2.49	0.93	0.68	0.51	na	na	2.60	0.70				
21	Cieślik‐Wegemund 2016_TUN_GS^58^	TUN	GS	91.00	13.00	14.3	2.60	0.70	0.80	1.67	na	na	2.60	0.69				
21	Cieślik‐Wegemund 2016_TUN_ATG^58^	TUN	ATG	95.00	11.00	71.4	2.50	0.78	1.00	1.61	na	na	1.90	1.40				
22	Nahas 2020_CAF_ATG^48^	CAF	ATG	82.14	na	68	2.2	1.20	1.20	1.10	na	na	2.00	1.40	*3.17*	3.2		
22	Nahas 2020_CAF_GS^48^	CAF	GS	77.7		60	2.00	1.20	0.30	0.70	na	na	1.74	0.24	0.8	0.9		
23	Kuka 2018_CAF_NO_ADG^46^	CAF	NO_ADJ	74.63	8.05	33.3	2.51	0.33	0.65	0.47	0.1	0.1	2.10	0.61				
23	Kuka 2018_CAF_BA^46^	CAF	BA	88.36	15.45	52	2.75	0.35	0.70	0.42	0.5	0.1	1.31	0.92				
24	Trivedi 2024_PST_NO_ADG^66^	PST	NO_ADJ	72.31	38.25	59.3	0.98	0.30	0.79	0.73	na	na	1.78	1.01				
24	Trivedi 2024_PST_BA^66^	PST	BA	86.31	26.02	75	1.29	0.69	1.49	0.91	na	na	2.17	0.81				
25	Ahmedbeyli 2014_CAF_NO_ADG^39^	CAF	NO_ADJ	74.99	28.07	50	2.37	0.83	0.60	0.36	0.1	0.0	2.75	0.54				
25	Ahmedbeyli 2014_CAF_GS^39^	CAF	GS	94.84	12.09	83.33	3.08	0.51	1.21	0.23	0.7	0.1	3.93	1.28				
26	Ozcelik 2011_CAF_NO_ADG^49^	CAF	NO_ADJ	89.1	14.3	61	3.89	0.98	0.66	0.95	na	na	4.69	1.29				
26	Ozcelik 2011_CAF_OB^49^	CAF	OB	96.2	9.4	84.6	4.65	0.99	0.48	0.97	na	na	1.10	0.91				
27	Pietruska 2018_TUN_GS^64^	TUN	GS	53.2	na	20	1.00	0.69	0.52	0.65	0.3	0.4	1.54	0.82				
27	Pietruska 2018_TUN_ATG^64^	TUN	ATG	83.1	na	67	1.54	0.58	2.78	1.53	1.1	0.5	1.50	1.20				
28	Santamaria 2022_CAF_GS^51^	CAF	GS	63.6	18.5	50.7	1.57	0.80	0.30	0.70	0.3	0.3	1.50	1.10				
28	Santamaria 2022_CAF_ATG^51^	CAF	ATG	91.3	18.00	72.9	2.3	0.80	0.96	1.20	0.9	0.6	3.08	0.72				
29	Potey 2019_CAF_OB^50^	CAF	OB	93.17	13.23	78.66	2.72	0.67	0.88	0.58	na	na	3.13	0.68			70.00	29.91
29	Potey 2019_CAF_OB_BA^50^	CAF	OB_BA	95.68	10.13	82.66	2.81	0.66	1.14	0.57	na	na	na	na			65.00	28.56
30	Rajeswari 2021_CAF_BA^41^	CAF	BA	93.95			2.39	0.53	0.45	0.73	0.3	0.2	na	na				
30	Rajeswari 2021_VISTA_BA^41^	VISTA	BA	96.84			2.26	0.81	0.32	1.01	0.4	0.2	2.35	0.57				
31	Ahmedbeyli 2019_CAF_GS^43^	CAF	GS	90.84	12.83	70.37	2.78	0.50	1.28	0.28	na	na	2.39	0.61				
31	Ahmedbeyli 2019_CAF_GS^43^	CAF	GS	95.69	9.52	82.2	2.89	0.52	1.34	0.33	na	na	1.42	0.86				
32	Chen 2023_VISTA_ATG^57^	VISTA	ATG	91.13	16.96	70.97	2.08	0.63	0.61	0.64	0.4	0.6	1.61	1.02	3.17	3.82		
32	Chen 2023_TUN_ATG^57^	TUN	ATG	91.4	13.53	67.86	2.3	0.67	0.54	0.86	0.7	0.5	2.40	0.79	3.60	3.92		
33	Cairo 2016_CAF_NO_ADG^54^	CAF	NO_ADG	na	na	47	2.4	0.70	−0.4	0.90	−0.002	0.1	3.00	0.69			28.90	7.00
33	Cairo 2016_CAF_ATG^54^	CAF	ATG	na	na	83	3.00	0.70	1.80	0.60	0.7	0.2	na	na			44.00	9.30
34&35	Tonetti 2018_Pelekos 2019_CAF_ATG^36^, ^38^	CAF	ATG	na	na	70	2.1	1.00	0.50	1.20	na	na	na	na	8.60	16.20		
34&35	Tonetti 2018_Pelekos 2019_CAF_GS^36^, ^38^	CAF	GS	na	na	48	1.7	1.10	−0.1	1.10	na	na	na	na	3.90	10.00		

Abbreviations: ADJ, adjunctives; ADM, allodermic membrane; ATG, autogenous graft (any type of technique); BA, biologic agents (enamel matrix derivative, platelet rich fibrin, chorion membrane, hyaluronic acid, growth factors, recombinant human factors); CAF, coronally advanced flap; TUN: tunnel; CAL, clinical attachment level; CM, collagen membrane; CRC, complete root coverage; FGG, free gingival graft; GS, gingival substitute; GT, gingival thickness; KT, keratinized tissue; MRC, mean root coverage; OB, orthodontic application; PST, Pinhole surgical technique; RecRed, recession reduction; VAS, Visual Analog Scale; VISTA, Vista.

#### 3.4.1 Primary outcomes

Both professional and patient‐determined esthetic outcomes were retrieved from 16 RCTs^23^, ^34^, ^35^, ^36^, ^38^, ^45^, ^48^, ^49^, ^50^, ^51^, ^52^, ^53^, ^54^, ^56^, ^57^, ^60^, ^61^, ^62^ while only patient esthetic satisfaction was investigated in five studies.^41^, ^42^, ^44^, ^55^, ^59^ In 22 papers RES was used as objective method to assess the esthetic outcomes of the treatment,^36^, ^39^, ^40^, ^43^, ^45^, ^46^, ^47^, ^49^, ^50^, ^51^, ^52^, ^53^, ^54^, ^56^, ^57^, ^60^, ^61^, ^62^, ^63^, ^64^, ^65^, ^66^ three trials adopted VAS through color and contour evaluation,^21^, ^23^, ^58^ one study^34^ applied the SEI method and one study^48^ implemented VAS for the overall esthetic appearance. Regarding patient esthetic assessments, 18 studies used VAS^23^, ^35^, ^41^, ^44^, ^48^, ^49^, ^50^, ^51^, ^52^, ^53^, ^54^, ^55^, ^56^, ^57^, ^59^, ^60^, ^61^, ^62^ and two studies^42^, ^45^ adopted a verbal scale.

#### 3.4.2 Secondary outcomes

Patient morbidity, defined as postoperative pain assessed at 7 days postsurgery, was primarily evaluated using VAS with varying endpoints: 0–10 (where 0 indicates no pain and 10 represents maximum pain)^35^, ^38^, ^40^, ^48^, ^53^, ^57^, ^59^, ^60^, ^61^, ^63^; 0–100 (where 0 indicates no pain and 100 indicates maximum pain)^44^, ^45^, ^50^, ^54^, ^56^, ^62^; and 0–100 reverse (where 0 indicates maximum pain and 100 indicates no pain).^21^, ^58^ Hypersensitivity was assessed in only a few studies^35^, ^38^, ^40^, ^41^, ^42^, ^45^, ^48^, ^51^, ^54^, ^63^ using different scales, such as VAS and the presence or absence of hypersensitivity. All studies provided data on recession reduction, either as direct values or indirectly through the comparison of baseline and final recession depths. Additionally, the percentage of mean and complete root coverage was reported for most studies. Results related to other significant secondary outcomes, such as CRC, GT change, KT change, and CAL change, are discussed in the following paragraph titled “Main effects of intervention.”

### 3.5 Main effects of interventions

A summary of the meta‐analysis of primary and secondary outcomes is presented in Table S7.

#### 3.5.1 Primary outcomes

RES was the most frequently utilized parameter for professional esthetic evaluation, assessed in 22 studies, corresponding to 43 treatment arms, and demonstrating a consistently high overall score (WME = 8.31; 95% CI [8.11, 8.50]; *p* < 0.001) with considerable heterogeneity (I^2^ = 93.2%; p < 0.001). Regarding the evaluated techniques, CAF with the use of an adjunctive (WME = 8.08; 95% CI [7.82, 8.34]; *p* < 0.001) and the tunnel technique (WME = 8.53; 95% CI [8.23, 8.83]; p < 0.001) achieved the most favorable esthetic outcomes from a professional standpoint compared to Vista, PST, and FGG.

From the patient's perspective, significant esthetic enhancements were reported, as evidenced by consistently high VAS scores across 16 studies, corresponding to 32 treatment arms (WME = 8.59; 95% CI [8.29, 8.89]; *p* < 0.001) displaying high heterogeneity across the included studies (I2 = 93.9%; *p* < 0.001). Comparable outcomes were observed between CAF, with or without adjunctive (including ATG, BA, and GS) (WME = 8.52; 95% CI [8.02, 9.02]; *p* < 0.001) and the tunneling approach (WME = 8.61; 95% CI [8.35, 8.88]; p < 0.001).

**FIGURE 4:**
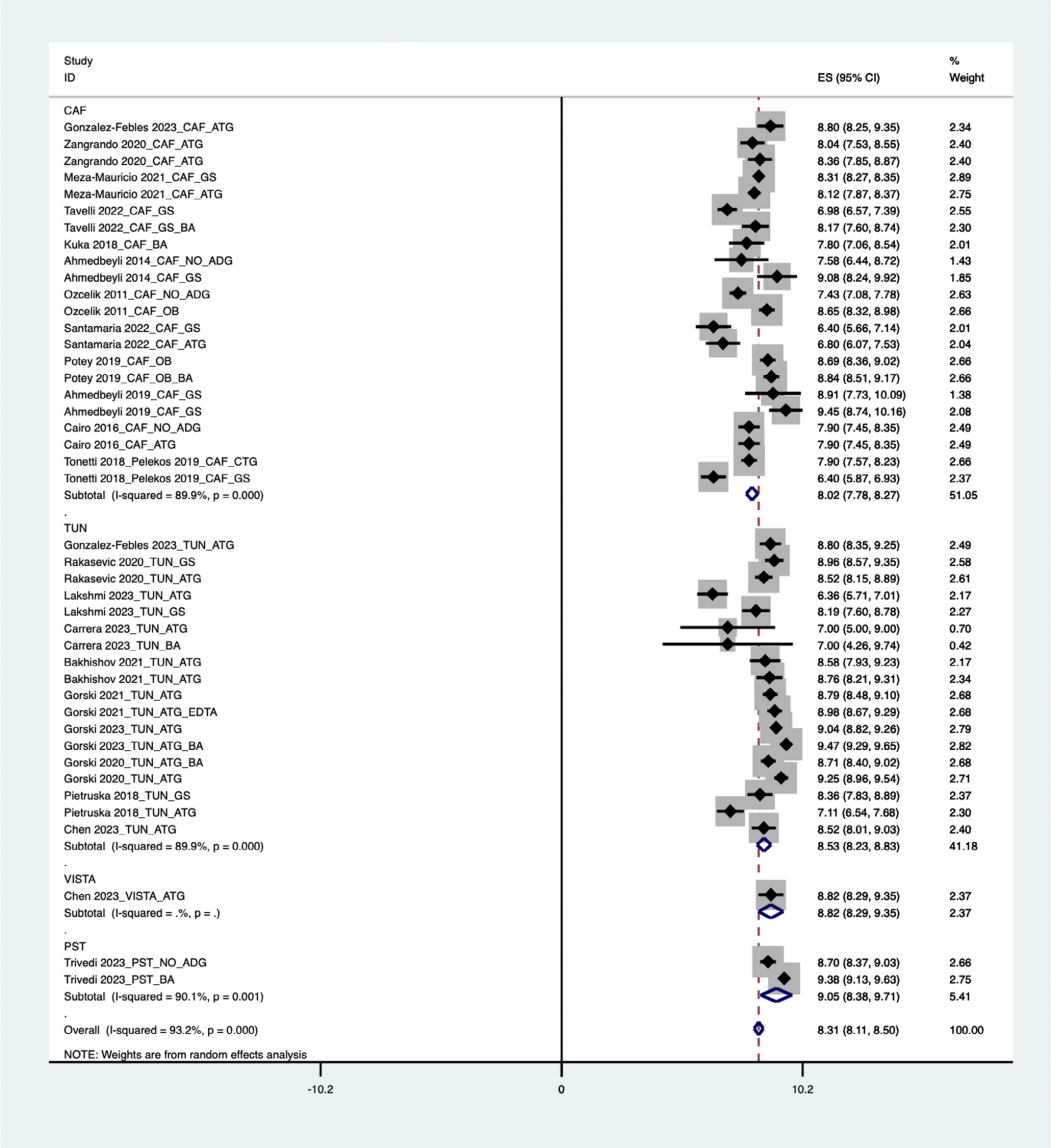
Forest plot from random effects of meta‐analysis evaluating the difference in RES after surgical treatments [weighted mean difference, 95% confidence interval (CI)]. ADJ, adjunctives; ADM, allodermic membrane; ATG, autogenous graft (any type of technique); BA, Biologic agents (enamel matrix derivative, platelet‐rich fibrin, chorion membrane, hyaluronic acid, growth factors, recombinant human factors); CAF, coronally advanced flap; CM, collagen membrane; FGG, free gingival graft; GS, gingival substitute; OB, orthodontic application; PST, pinhole surgical technique; TUN, tunnel; VISTA, Vista.

**FIGURE 5:**
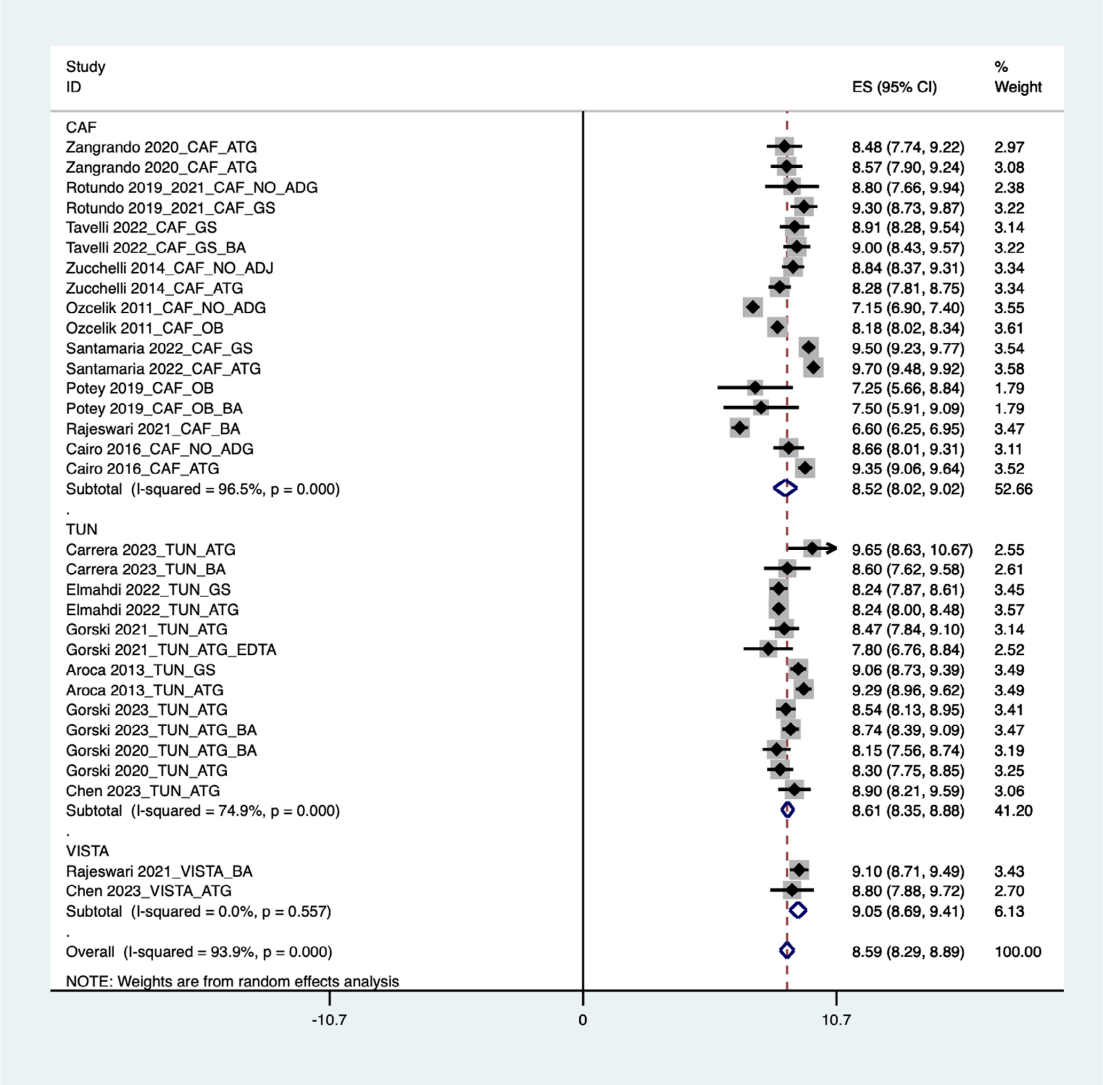
Forest plot from random effects of meta‐analysis evaluating the difference in Patient Esthetic VAS after surgical treatments [weighted mean difference, 95% confidence interval (CI)]. CAF = coronally advanced flap; TUN = tunnel; Vista = Vista; PST = pinhole surgical technique. ADJ, adjunctives; ADM, allodermic membrane; ATG, autogenous graft (any type of technique); BA, Biologic agents (enamel matrix derivative, platelet‐rich fibrin, chorion membrane, hyaluronic acid, growth factors, recombinant human factors); CAF, coronally advanced flap; CM, collagen membrane; FGG, free gingival graft; GS, gingival substitute; OB, orthodontic application; PST, pinhole surgical technique; TUN, tunnel; VISTA, Vista.

Results are depicted in Figure 4 and Figure 5 through forest plots.

#### 3.5.2 Secondary outcomes

##### 3.5.2.1 Root coverage

Analysis from 26 studies, corresponding to 52 arms, revealed an overall statistically significant improvement in mean root coverage percentage with a WME of 82.59% (95% CI [71.33, 93.86]; *p* < 0.001) although high heterogeneity was observed (I^2^ = 99.8%; *p* < 0.001). CAF procedures reported the highest MRC (WME = 86.53%; 95% CI [83.31, 89.75]; *p* < 0.001) followed by the tunneling technique (WME = 80.85%; 95% CI [64.50, 97.21]; *p* < 0.001) (Figure S1).

In the context of CRC, an analysis of 30 studies and 60 treatment arms revealed a statistically significant overall CRC rate, with a WME of 62.7% (95% CI [57.0, 68.4]; *p* < 0.001) with a consistent trend between the CAF and tunneling procedures. The findings indicate substantial interstudy heterogeneity, reflected by an I^2^ value of 94.06% (*p* < 0.001).

##### 3.5.2.2 Recession reduction (RecRed)

The depth of the recession defects was evaluated in all 30 included studies (60 arms), reporting an overall significant reduction with a weighted mean estimate (WME) of 2.22 mm (95% CI [2.11; 2.32]; *p* < 0.001) with considerable heterogeneity (*I* ^2^ = 99.2%, *p <* 0.001) (Figure S2).

##### 3.5.2.3 Other relevant clinical parameters

Keratinized tissue change (KT change) was evaluated across 30 studies (60 arms) reporting a low but statistically significant improvement, with a WME of 0.74 mm (95% CI [0.59; 0.89]; *p* < 0.001). However, there was considerable heterogeneity observed in the results, with an I^2^ value of 96.9% (p < 0.001). The comparisons between the CAF with adjunctive procedures and tunneling procedures showed similar outcomes regarding KT change (Figure S3).

(Figure S3).

Gingival thickness change (GT change) was evaluated in 19 studies (38 arms), reporting the greatest improvement when CAF was implemented alongside adjunctive and tunneling techniques, yielding an overall mean weighted effect (WME) of 0.56 mm (95% CI [0.43; 0.68]; *p* < 0.001) with considerable heterogeneity (*I* ^2^ = 99.6%; *p* < 0.001) (Figure S4).

Clinical attachment level (CAL change), evaluated in 28 studies and 56 arms, reported a statistically significant change with an overall WME of 2.17 mm (95% CI [1.97; 2.38]; *p* < 0.001), alongside significant heterogeneity (*I* ^2^ = 97.3%, p < 0.001). CAL change was primarily observed when CAF was performed with an adjunctive treatment (Figure S5).

##### 3.5.2.4 Postoperative pain

Patient postoperative pain was primarily assessed using VAS with different endpoints. Overall, the perceived pain was very low across all techniques, with a WME of 2.67 (95% CI [2.28; 3.06]; *p* < 0.001) and 24.34 (95% CI [16.46; 32.22]; *p* < 0.001) for VAS 0–10 (9 studies and 18 arms) and VAS 0–100 (5 studies and 10 arms), respectively. The included studies that evaluated pain using both scales exhibited considerable heterogeneity (VAS 0–10: *I* ^2^ = 99.3% (*p* < 0.001) and VAS 0–100: *I* ^2^ = 98.4% (*p <* 0.001)) (Figure S6).

##### 3.5.2.5 Metaregression analysis

**FIGURE 6:**
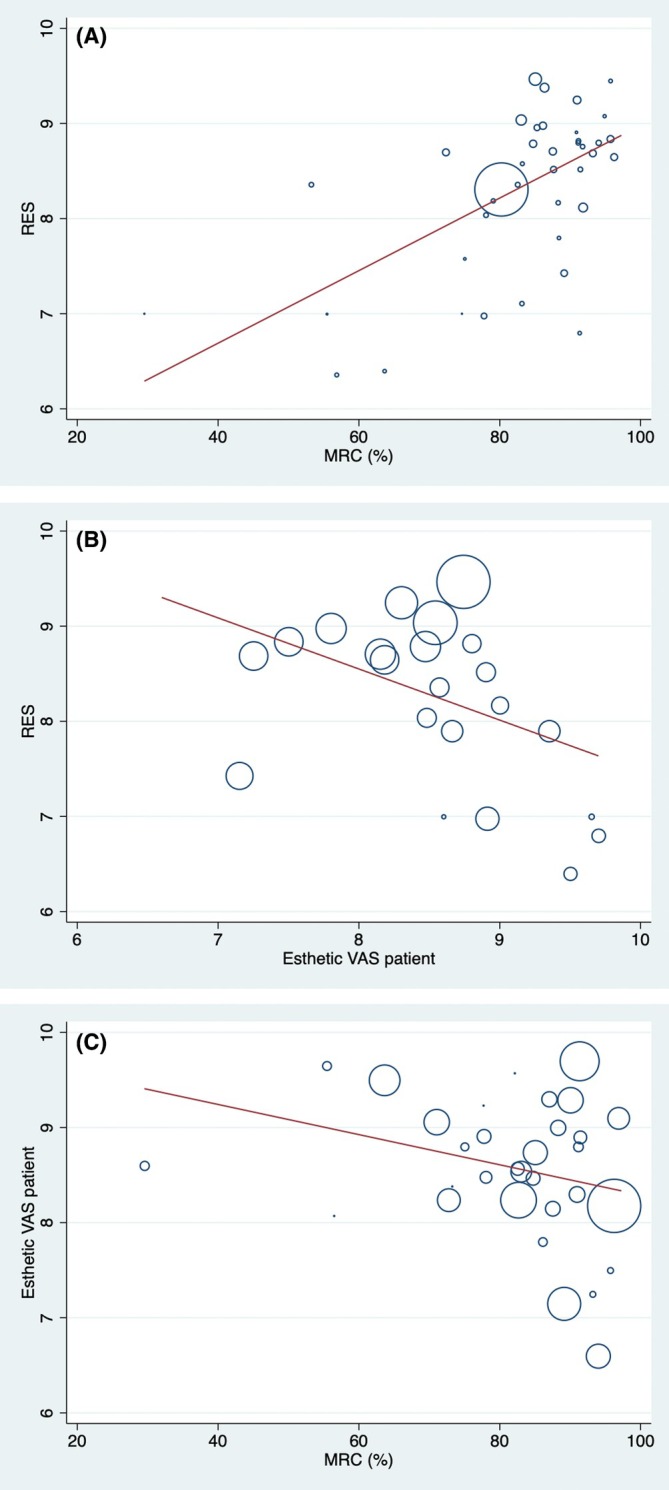
(A) Metaregression showing a statistically significant positive linear association between mean root coverage (MRC) and RES (root coverage esthetic score). Equation: RES = 0.038 + 5.16* MRC; *R* ^2^ (adjusted) = 25.41%; *p* = 0.001. (B) Metaregression showing a negative linear association between RES and Esthetic VAS patient. Equation: RES = 12.85–0.54*Esthetic VAS Patient; *R* ^2^ (adjusted) = 13.15%; *p* = 0.042. (C) Metaregression showing a lack of linear association between MRC and Esthetic VAS patient. Equation: Esthetic VAS patient = 9.87–0.02*MRC; *R* ^2^ (adjusted) = 4.07%; *p* = 0.155 a.

Metaregression analyses and scatter plots (Figure 6) revealed a statistically significant positive linear association between mean root coverage (MRC) and RES (eprf_res = 5.25 + 0.037* MRC; *p* < 0.001; *R* ^2^ = 0.345); a negative linear association between RES and Esthetic VAS patient (eptf_vas = 12.68–0.50*eprf_res; *p* = 0.016; *R* ^2^ = 0.257) and lack of linear association between MRC and Esthetic VAS patient (eptf_vas = 10.04–0.018* MRC; *p* = 0.094: *R* ^2^ = 0.091).

## 4 DISCUSSION

This systematic review was designed to provide a comprehensive understanding of the effects of root coverage procedures for multiple gingival recessions in terms of esthetics and patient‐reported outcomes. To date, this is the most robust evaluation of professionally assessed esthetic outcomes and patient‐reported outcomes (PROMs) including 32 RCTs and over 1000 patients. Our findings offer clear evidence that MGR can be predictably treated using a variety of surgical techniques, with high esthetic satisfaction reported by both clinicians and patients. In particular, CAF and tunnel techniques, especially in combination with ATG or GS, produced the most favorable outcomes across nearly all domains. These esthetic improvements were paralleled by significant gains in mean and complete root coverage, gingival thickness, keratinized tissue, and clinical attachment level, all accompanied by minimal postoperative discomfort. Although subgroup analyses suggest differences among techniques, the present study was not designed to perform direct comparisons between interventions.

### 4.1 Summary of main findings and comparison with other data

#### 4.1.1 Primary outcome variables: Professional and patient‐reported esthetic evaluation

Professional esthetic assessment was reported in 22 studies using mostly RES and indicated consistently high overall scores (WME = 8.31; 95% CI [8.11, 8.50]; *p* < 0.001), with CAF combined with adjunctives (WME = 8.08; 95% CI [7.82, 8.34]; *p* < 0.001) and TUN (WME = 8.53; 95% CI [8.23, 8.83]; *p* < 0.001) exhibiting statistically significantly superior results than Vista, PST, and FGG. From the patient's perspective, VAS was the most common assessment method that indicated significant esthetic improvements, with comparable outcomes for TUN and CAF with/without adjunctives. Nonetheless, high heterogeneity was observed across the included studies, which may be attributed to center effects, study methodology, and operator skills.

Our results corroborate those reported by Cairo et al., who included in their SR of RCTs both single as well as MGR, showing greater statistically significant esthetical improvements as assessed by RES with TUN + ATG (WMD 0.84) and CAF + ATG/GS (WMD 0.74; WMD 0.55) as compared to CAF alone.^68^ No significant differences were observed between CAF + ATG and TUN + ATG.^68^ VAS values for patient satisfaction were significantly higher for CAF + ATG as compared to CAF alone (WMD 5.91), and higher for ATG (WMD 8.12) and ATG + EMD (WMD 4.31) than to flap alone.^68^ In a consecutive subanalysis, significantly less natural tissue texture (WMD: −0.21) and poorer color integration (WMD: −0.06) were observed for the addition of ATG as compared to CAF alone.^68^

In a previous SR of the same group that assessed by means of a Bayesian network meta‐analysis the efficacy of periodontal plastic procedures in improving esthetics for the treatment of single and multiple gingival recession, CAF + ATG, CAF + acellular dermal matrix (ADM), and autologous fibroblasts showed the best professional esthetical results using RES (best probability 24% and 64%, respectively).^69^ From the patient's perspective, higher VAS scores were reported for CAF + ATG and CAF + Enamel Matrix Derivatives (EMD) (best probability 44% and 26%, respectively). Similar results were also reported by another group, indicating no significant differences comparing TUN and CAF and a high heterogeneity among the studies.^67^

The professional esthetic perception (using RES) in the present SR was significantly correlated with the level of mean RC (eprf_res = 5.25 + 0.037* MRC; *p* < 0.001; *R* ^2^ = 0.345). Interestingly, this correlated linearly negatively with the patient's perception (VAS). Moreover, no linear correlation could be seen between mean RC and the patient esthetic VAS (eptf_vas = 10.04–0.018* MRC; *p* = 0.094: *R* ^2^ = 0.091), indicating that probably any improvement (i.e., incomplete root coverage) in a recession defect, may be sufficient to enhance the patient's satisfaction.

#### 4.1.2 Secondary outcome variables‐ clinical parameters

In the current SR, an overall significant mean RC (WME 82.59%, 95% CI [71.33, 93.86]; *p* < 0.001) and CRC (WME 62.7%, 95% CI [57.0, 68.4]; p < 0.001) were observed, with CAF showing the highest mean RC followed by the tunnel technique. As expected, these results are followed by an overall significant reduction in the recession depth (WME, 2.22 mm, 95% CI [2.11; 2.32]; p < 0.001). However, substantial heterogeneity among all studies was noted. In agreement with our results, a WMD of 4.38 mm (95% CI –9.06, 17.83; *p* = 0.52) between TUN and CAF was reported in the SR of Tavelli et al.,^67^ also indicating a substantial heterogeneity among the studies (I^2^ = 93.37%, p < 0.001). Their overall analysis for CRC could not show any significant differences between CAF or TUN. However, the subgroup analyses for ATG or ADM indicated statistically significantly more CRC for CAF + ATG or CAF + ADM compared to TUN + ATG or TUN + ADM.^67^ In the SR of Cairo et al.,^68^ higher levels of the gingival margin were obtained for CAF + ATG and TUN + ATG versus CAF alone, and no statistically significant differences were observed between TUN + GS or CAF + GS.^68^ No direct values for recession reduction, mean RC, or CRC were assessed and reported in their SR; however, we can indirectly relate to their other clinical parameters, including the level of GM. A further SR that included only two RCTs with MGR, emphasized as a result of their meta‐analysis, statistically significantly more depth change in GR and KT width change for CAF + ATG versus CAF + PRF.^16^ Also in agreement with our results is the SR of Graziani et al.^12^ They reported for Miller Class I/II CRC values of 23.8%–89.3% and a significant partial root coverage of 86.27% (*p* < 0.01). This was in line with the values for recession reduction that reached a WMD of 2.53 mm (*p* < 0.01).

Even though lower values, statistically significant changes in the keratinized tissue width and gingival thickness were observed in all included studies that evaluated this parameter, reaching a weighted mean estimate of 0.74 mm (95% CI [0.59; 0.89]; *p* < 0.001) and 0.56 mm (95% CI [0.43; 0.68]; *p* < 0.001) respectively. No significant differences between CAF with adjunctives and tunneling procedures were observed for KT, both showing the greatest GT increase. Corroborating our data, Tavelli et al. could not point out a statistically significant difference for KT gain between TUN or CAF, except for the results of a subgroup analysis for the studies using ADM, where significantly more KT gain was obtained with CAF (WMD 0.36 mm; *p* < 0.001).^67^ Also in line with our results, Graziani et al. reported for Miller Class I/II recessions a KT gain of 0.35 mm (*p* < 0.06) that didn't reach statistical significance.^12^

#### 4.1.3 Patient‐reported outcomes

Pain is an important aspect for patients when deciding on a surgical recession coverage. Interestingly, the postoperative pain as assessed by VAS in our SR, either on a 0–10 scale or on a 0–100 scale, showed a low but significant level of pain (WME 2.67, 95% CI [2.28; 3.06]; *p* < 0.001 and WME 24.34, 95% CI [16.46; 32.22]; *p* < 0.001, respectively). In the studies using CAF (8 studies with VAS‐scale 0–10, 5 studies with VAS‐scale 0–100), the meta‐analysis evidenced statistically significant more pain when adjunctives (i.e., grafts) were used (VAS 0–10: WMD 3.75, *p* < 0.001; VAS 0–100: WMD 45.75, *p* = 0.002). Statistically significant higher pain scores were observed also for the tunneling technique (9 studies with VAS‐scale 0–10: WMD 2.25, *p* < 0.001, 5 studies with VAS‐scale 0–100: WMD 24.34, *p* = 0.003). No direct comparisons were performed between these two techniques (TUN vs. CAF).

Root coverage surgical procedures for treating multiple gingival recessions seem to show a minimal impact on postoperative pain, with patients reporting very low levels of discomfort. Although the procedures involve multiple teeth, the surgery duration in the studies included in the current SR was relatively short (ranging from 25.23 ± 1.87 min to 79.4 ± 5.6 min). However, it is important to mention that excepting one study that did not report on this information,^58^ in all studies, painkillers were prescribed. Furthermore, in the majority of the postoperative protocols, antibiotics were also given, probably explaining the low levels of postoperative pain. Noteworthy to mention is that most studies included nonsmokers, which may have also contributed to a complication‐free healing with lower pain levels, as reported by other authors.^70^ Nonetheless, data from another research group, evidenced in their analysis a significantly higher patient morbidity for the ATG groups (CAF + ATG, TUN + ATG, ATG‐based groups) as compared to flap alone.^68^ Despite the fact that patient morbidity is an important outcome measure for PROMs, this is not always assessed and reported in studies, and consequently, also not included in the majority of SRs.

To the best of our knowledge, this is the first systematic review and meta‐analysis to explore both professional assessments and patient‐perceived esthetic outcomes following the surgical management of multiple gingival recessions. While a prior review by Graziani et al. predominantly concentrated on root coverage, demonstrating the effectiveness of periodontal plastic surgery, the current analysis adopts a broader perspective by including patient‐reported outcome measures (PROMs).^12^ This expanded focus underscores the increasing importance of patient satisfaction and subjective esthetic evaluations as key indicators of clinical success, reflecting the ongoing shift in periodontal research toward a more patient‐oriented paradigm.

Our review underscores the potential of alternative approaches, including the use of collagen matrices (CMs), acellular dermal matrices (ADMs), and biologic agents (BAs). Procedures combining CAF with CMs or BAs showed promising results in both professional evaluations and patient satisfaction metrics, suggesting that these techniques may serve as viable options, especially for patients who cannot undergo autogenous grafting. Notably, techniques such as the tunnel approach (TUN) combined with biologics also demonstrated significant esthetic improvements, highlighting their utility in specific clinical scenarios.

## 5 LIMITATIONS

One of the limitations of the current SR is the limited number of studies that employed a common measure outcome for professional and/or patient‐related esthetic evaluation leading to an impediment in performing meta‐analyses for all the intended primary and secondary outcomes. The high heterogeneity among included studies, particularly in terms of surgical techniques, graft materials, and outcome measures, poses challenges for direct comparisons.

The limited number of trials per intervention‐outcome combination reduced the precision of some meta‐analyses. Consequently, these statistical outcomes (meta‐analyses) are based solely on results from a very limited number of trials that followed the same intervention and outcome combination, which calls for careful and cautious interpretation of the results.

A further limitation of the included studies is reflected in the risk of bias analysis. Two studies included in the professional esthetic evaluation group were classified as high risk due to missing outcome data, issues related to the intervention, and limitations for trial participant exclusion. Almost all studies showed a moderate risk when assessing deviations from the intended interventions, which consecutively raised concerns about the outcome parameters (Figures 2 and 3). High risk of bias was assigned to three trials providing input for esthetic patient satisfaction due to issues related to the evaluation of outcome measures or deviations from the intended interventions (Figures 2 and 3). These risk‐of‐bias assessments suggest there is no unanimity in assessing esthetic outcome measures and that greater care should be taken to follow the intended interventions.

Further limitations of the included studies comprise: (1) The limited (short) follow‐up durations, limiting thus the ability to assess the long‐term stability of outcomes and patient satisfaction over time; (2) The lack of individual patient data (although Authors were specifically contacted) determining the combination of defect‐level and patient level outcomes; (3) The combination of split‐mouth (*n* = 11) and parallel (*n* = 24) studies, which might “artificially increase the weight” of split‐mouth studies in the meta‐analyses.

### 5.1 Recommendations for future research

The lack of trials assessing similar outcome measures for professional esthetic evaluation highlights the urgent need for future research to incorporate objective parameters for esthetic assessments with data reported in a standardized manner, with a consistent unit of evaluation (i.e., “universal scale”) for both the patient and the site. Another important parameter would be the objective assessment of hypersensitivity in a standardized manner (i.e., Schiff's score) which was infrequently reported and often assessed using different scales.

## 6 CONCLUSIONS

The results of this systematic review and meta‐analysis, focusing on professional and patient‐related esthetic evaluation after surgical treatment of multiple gingival recessions, suggest that:

- CAF and TUN with adjunctive use of ATG support esthetic improvement from both professional and patient perspectives.
- CAF and TUN with adjunctive use of ATG or GS are effective in root coverage outcomes with minimal postoperative morbidity.

## 7 CLINICAL RELEVANCE

### 7.1 Scientific Rationale for the Study

While root coverage procedures for multiple gingival recessions are well‐established and generally predictable, there is a notable gap in research regarding their esthetic results and the impact on patient satisfaction.

### 7.2 Principal Findings

All surgical techniques evaluated demonstrate esthetic improvements from both professional and patient perspectives. Adjunctive soft tissue augmentation, using either autogenous connective tissue or replacement grafts in combination with biologic agents, may further enhance esthetic outcomes, promoting more natural tissue integration.

### 7.3 Practical Implications

Root coverage procedures for multiple gingival recessions are effective treatments. Adjunctive techniques involving connective tissue or replacement grafts with biologic agents improve both esthetic results and patient satisfaction. However, the current evidence highlights the need for longitudinal studies with robust head‐to‐head comparisons and standardized metrics to advance the field and refine clinical practices.

## FUNDING INFORMATION

There are no funding sources. The study was self‐supported.

## CONFLICT OF INTEREST STATEMENT

The authors declare no conflicts of interest.

## ETHICS STATEMENT

The authors have nothing to report.

## CONSENT STATEMENT

The authors have nothing to report.

## Supporting information


**Table S1.** Reasons for exclusion of studies after full‐text assessment.
**Table S2**. Risk of bias assessment for the included RCTs using The Cochrane Risk of Bias Tool 2 (RoB2) for Randomized Controlled Trials studies (Sterne et al. 2019) for Professionally‐determined Esthetic Outcome.
**Table S3**. The individual assessment of the risk of bias for Professionally‐determined Esthetic Outcome.
**Table S4**. Risk of bias assessment for the included RCTs using The Cochrane Risk of Bias Tool 2 (RoB2) for Randomized Controlled Trials studies (Sterne et al. 2019) for Patient‐determined Esthetic Outcome.
**Table S5**. Individual Risk of bias assessment for Patient‐determined Esthetic Outcome.
**Table S6**. Descriptive demographic data and characteristics of the studies.
**Table S7**. Summary of the meta‐analysis of primary and secondary outcomes.
**Figure S1**. Forest plot from random effects of a meta‐analysis evaluating the difference in percentage of mean root coverage among techniques [weight mean difference. 95% confidence interval (CI)]. CAF = coronally advanced flap; TUN = tunnel; Vista = Vista; PST = Pinhole surgical technique.
**Figure S2**. Forest plot from random effects of a meta‐analysis evaluating the difference in recession reduction among techniques [weight mean difference. 95% confidence interval (CI)]. CAF = coronally advanced flap; TUN = tunnel; Vista = Vista; PST = Pinhole surgical technique.
**Figure S3**. Forest plot from random effects of a meta‐analysis evaluating the difference in keratinized tissue change among techniques [weight mean difference. 95% confidence interval (CI)]. CAF = coronally advanced flap; TUN = tunnel; Vista = Vista; PST = Pinhole surgical technique.
**Figure S4**. Forest plot from random effects of a meta‐analysis evaluating the difference in gingival thickness change among techniques [weight mean difference. 95% confidence interval (CI)]. CAF = coronally advanced flap; TUN = tunnel; Vista = Vista; PST = Pinhole surgical technique.
**Figure S5**. Forest plot from random effects of a meta‐analysis evaluating the difference in clinical attachment level change among techniques [weight mean difference. 95% confidence interval (CI)]. CAF = coronally advanced flap; TUN = tunnel; Vista = Vista; PST = Pinhole surgical technique.
**Figure S6**. Forest plot from random effects of a meta‐analysis evaluating the difference in postoperative pain among techniques through VAS 0–10 (a) and VAS 0–100 (b) [weight mean difference. 95% confidence interval (CI)]. CAF = coronally advanced flap; TUN = tunnel; Vista = Vista; PST = Pinhole surgical technique.

## Data Availability

The data that support the findings of this study are available on request from the corresponding author. The data are not publicly available due to privacy or ethical restrictions.
