# Supplementary material for: Esthetical and patient‐reported outcomes after root coverage procedures for multiple gingival recessions: A systematic review and meta‐analysis
Source: Periodontol 2000. 2026 May 14;99(1):21–41. doi: 10.1111/prd.70050 (PMC13428093; doi:10.1111/prd.70050)
Supplement: Supplementary file 1 — Table S1. Reasons for exclusion of studies after full‐text assessment. Table S2. Risk of bias assessment for the included RCTs using The Cochrane Risk of Bias Tool 2 (RoB2) for Randomized Controlled Trials studies (Sterne et al. 2019) for Professionally‐determined Esthetic Outcome. Table S3. The individual assessment of the risk of bias for Professionally‐determined Esthetic Outcome. Table S4. Risk of bias assessment for the included RCTs using The Cochrane Risk of Bias Tool 2 (RoB2) for Randomized Controlled Trials studies (Sterne et al. 2019) for Patient‐determined Esthetic Outcome. Table S5. Individual Risk of bias assessment for Patient‐determined Esthetic Outcome. Table S6. Descriptive demographic data and characteristics of the studies. Table S7. Summary of the meta‐analysis of primary and secondary outcomes. Figure S1. Forest plot from random effects of a meta‐analysis evaluating the difference in percentage of mean root coverage among techniques [weight mean difference. 95% confidence interval (CI)]. CAF = coronally advanced flap; TUN = tunnel; Vista = Vista; PST = Pinhole surgical technique. Figure S2. Forest plot from random effects of a meta‐analysis evaluating the difference in recession reduction among techniques [weight mean difference. 95% confidence interval (CI)]. CAF = coronally advanced flap; TUN = tunnel; Vista = Vista; PST = Pinhole surgical technique. Figure S3. Forest plot from random effects of a meta‐analysis evaluating the difference in keratinized tissue change among techniques [weight mean difference. 95% confidence interval (CI)]. CAF = coronally advanced flap; TUN = tunnel; Vista = Vista; PST = Pinhole surgical technique. Figure S4. Forest plot from random effects of a meta‐analysis evaluating the difference in gingival thickness change among techniques [weight mean difference. 95% confidence interval (CI)]. CAF = coronally advanced flap; TUN = tunnel; Vista = Vista; PST = Pinhole surgical technique. Figure S5. Forest plot from random e [file PRD-99-21-s001.docx]

**Supplementary Appendix**

Aesthetics and Patient-Reported Outcomes after root coverage procedures for multiple gingival recessions. A systematic review and meta-analysis

Martina Stefanini^1^, Ilham Mounssif^1^, Elena Figuero^2^, Giovanni Zucchelli^3^, Anton Sculean^3^, Raluca Cosgarea^4,5,6^

**MATERIALS & METHODS**

**Page 2 Search key terms strategy for the electronic databases**

**Page 2 Study selection**

**Page 3 Data extraction and management**

**Page 3 Quality & risk of bias assessment**

**TABLES & REFERENCES**

Page 4 Table S1. Reasons for exclusion after full-text assessment.

Page 5 Table S2. Overall Risk of bias assessment for the included RCTs using The Cochrane Risk of Bias Tool 2 (RoB2) for Randomized Controlled Trials studies for Professionally-determined Aesthetic Outcome

Page 7 Tables S3 elaborating on the individual assessments for the potential risk of Bias for the included RCTs using The Cochrane Risk of Bias Tool for Randomized Controlled Trials studies for Professionally-determined Aesthetic Outcome

Page 69 Table S4. Overall Risk of bias assessment for the included RCTs using The Cochrane Risk of Bias Tool 2 (RoB2) for Randomized Controlled Trials studies for Patient-determined Aesthetic Outcomes

Page 72 Tables S5 elaborating on the individual assessments for the potential risk of Bias for the included RCTs using The Cochrane Risk of Bias Tool for Randomized Controlled Trials studies for Patient-determined Aesthetic Outcome

Page 133 Tables S6. General overview of the included studies

Page 139 Table S7. Summary of the meta-analysis of primary and secondary outcomes

Page 142 Figure S1. Forest plot comparing the mean root coverage

Page 143 Figure S2. Forest plot comparing the Recession reduction

Page 144 Figure S3. Forest plot comparing keratinized tissue change

Page 145 Figure S4. Forest plot comparing the gingival thickness change

Page 146 Figure S5. Forest plot comparing the clinical attachment change

Page 147 Figure S6. Forest plot comparing the postoperative pain

Page 148 References

**2.6 Search key terms strategy for the electronic databases**

A detailed computerized systematic search was conducted in the literature to identify eligible studies using three databases: MEDLINE (via PubMed). EMBASE. and Cochrane Central Register of Controlled Trials. The search terms were as follows. and a literature search was performed for studies published up to May 31. 2024:

1. The National Library of Medicine (MEDLINE by PubMed). using the strategy: (‘‘Gingival Recession/surgery’’ ^1^ OR ‘‘Gingival Recession/ therapy’’ ^1^) AND ((Humans ^1^) AND (Randomized Controlled Trial[ptyp]w1w));
2. The Cochrane Oral Health Group Trials Register. using the following strategy: ‘‘Gingival Recession’’ [Search All Text] AND ‘‘Root Coverage’’ [Search All Text];
3. EMBASE. utilizing the strategy: “Gingival Recession” ^1^ AND (Randomized Controlled Trial).

Moreover. a free-text-word research strategy using “multiple gingival recessions “and “root coverage procedures” was conducted on PubMed and the Cochrane Database with the filter “clinical trial.”

A final study collection file with all potentially eligible studies was created by merging items provided by the three different electronic searches. Due to time limitations. no hand search was done. and only publications written in English were included.

**2.7 Study selection**

Two calibrated examiners (IM and RC) screened the titles and abstracts (if available) of the entries identified in the search. in duplicate and independently. Next. the full-text version of all studies that potentially meet the eligibility criteria or for which there were insufficient information in the title and abstract to make a decision was obtained. Any article considered as potentially relevant by at least one of the reviewers was included in the next screening phase. Subsequently. the full-text publications was evaluated in duplicate and independently by the same review examiners. The examiners was calibrated with the first 10 full-text. consecutive publications. Any disagreement on the eligibility of the studies was resolved through open discussion between both reviewers until an agreement is reached or through settlement by an arbiter (MS). All articles that did not meet the eligibility criteria were excluded. and the reasons for exclusion were noted. The inter-examiner agreement following the full-text assessment was calculated using Kappa statistics. Disagreement on including the studies at any point was resolved in the same manner as previously mentioned.

Any missing information that could contribute to this systematic review was requested to the corresponding author(s) via email communication*.*

**2.8 Data extraction and management**

Two examiners (IM and RC) independently retrieved all relevant information from the included articles using a data extraction sheet designed for this review. At any stage. disagreements between the reviewers were resolved through open discussion and consensus. A third person (MS) would settle the debate if a disagreement persisted.

- Study characteristics such as study type and design, number of centers, geographic location, setting (university vs. private practice), and source of funding;
- Population characteristics: age of participants, number of participants and treated sites (baseline/follow-up), gender, follow-up period, and smoking habits
- Interventions: intervention and comparison
- Outcomes: outcomes specified and collected, and time points reported

Information needed to assess bias (e.g. any deviations from intended interventions, whether data were imputed for key outcomes. etc.).

**2.9 Quality and Risk of Bias assessment**

Two authors (IM and RC) independently and in duplicate assessed the risk of bias for the included studies. For RCTs. according to the recommended approach by the Cochrane Collaboration Group RoB 2.0 tool;^2^ ROBINS-I tool was used to determine the potential risk of bias for non-randomized cohort studies; the Joanna Briggs Institute Critical Appraisal tool was utilized for case series.^3^ Risk of bias judgments was summarized for each outcome across different studies for each of the domains listed. where the overall risk of bias for the result was the least favorable assessment across the domains of bias was condected. Any disagreement was discussed between the same authors. Another author (GZ) was consulted in case no agreement was reached. However. no study was excluded on the basis of the risk of bias within a study. The primary analysis was limited to studies at an overall low risk of bias; however. sensitivity analysis was performed that includes studies not rated at a low risk of bias. Results from the risk of bias assessment were presented in tables and/ figures.

**Table 1S**. Reasons for exclusion of studies after full-text assessment.

| **Reason** | **Reference** |
| --- | --- |
| Mixed single and multiple gingival recessions (n=22) | Carcuac et al. 2023.^4^ Stähli et al. 2023.^5^  Mercado et al. 2020.^6^ Bommala et al. 2023.^7^  Geisinger et al. 2022.^7^ Peter et al. 2023.^8^ George et al. 2018.^9^ Milinkovic et al. 2015.^10^ Roman et al. 2013.^11^ Suzuki et al 2020.^12^ McGuire et al. 2021.^13^  Parlak et al. 2023.^14^ Zucchelli et al. 2010.^15^  Elena et al. 2024.^16^ Zuhr et al. 2014.^17^ Nickles et al. 2010.^18^ Cardaropoli et al. 2009.^19^ Boltchi et al. 2000.^20^ Damante et al. 2019.^21^ Nizam et al. 2015.^22^ George et al. 2023^9^ |
| Single recession (n=3) | Ucak Turer et al. 2019.^23^ Fernandes-Diaz et 2015.^24^ Barootchi et al. 2021^25^ |
| Not enough patients per arm (< 10) (n=2) | Pradahan et al. 2022^26^ Ongoz Dede et al. ^27^ |
| No root coverage purpose (n=2) | Cevallos et al. 2020;^28^ Naziker& Ertugrul 2023^29^ |

***3.4 Risk of Bias Assessment***

The individual and overall assessment of the risk of bias per outcome is presented in the following tables.

**Table S2.** Risk of bias assessment for the included RCTs using The Cochrane Risk of Bias Tool 2 (RoB2) for Randomized Controlled Trials studies (Higgins et al..2023; Sterne et al..2019) for Professionally-determined Aesthetic Outcome

| **Publication** | **Randomization process** | **Deviations from intended interventions** | **Missing outcome data** | **Measurement of the outcome** | **Selection of the reported result** | **Overall risk of bias** |
| --- | --- | --- | --- | --- | --- | --- |
| (Gonzalez-Febles et al. 2023)^30^ | Low | Low | Low | Some concerns | Low | Low |
| (Zangrando et al. 2020)^31^ | Low | Some concerns | Low | Low | Low | Some concerns |
| (Rotundo et al. 2021)^32^ | Low | Some concerns | Low | Low | Low | Some concerns |
| (Rakasevic et al. 2020)^33^ | Low | Some concerns | Low | Low | Low | Some concerns |
| (Lakshmi et al. 2023)^1^ | Low | Some concerns | Low | Low | Low | Some concerns |
| (Carrera et al. 2023)^34^ | Low | Some concerns | Low | Low | Low | Some concerns |
| (Bakhishov et al. 2020)^35^ | Low | High | High | Some concerns | Low | High |
| (Meza Mauricio et al. 2021)^36^ | Low | Some concerns | Low | Low | Low | Some concerns |
| (Gorski et al. 2022)^37^ | Low | Some concerns | Some concerns | Low | Low | Some concerns |
| (Cieslik et al. 2016)^38^ | Low | Some concerns | Low | Low | Low | Some concerns |
| (Tavelli et al. 2022)^39^ | Low | Some concerns | Low | Low | Low | Some concerns |
| (Zucchelli et al. 2014)^40^ | Low | Some concerns | Low | Low | Low | Some concerns |
| (Gorski et al.2023)^41^ | Low | Some concerns | Low | Low | Low | Some concerns |
| (Gorski et el. 2020)^42^ | Low | Some concerns | Low | Low | Low | Some concerns |
| (Zucchelli et al. 2009)^43^ | Low | Some concerns | Low | Low | Low | Some concerns |
| (Nahas et al. 2020)^44^ | Low | Some concerns | Low | Low | Low | Some concerns |
| (Kuka et al. 2018)^45^ | Low | Some concerns | Low | Low | Low | Some concerns |
| (Trivedi BDS et al. 2023)^46^ | Low | Some concerns | Low | Low | Low | Some concerns |
| (Ahmedbeyli et al. 2014)^47^ | Some concerns | Some concerns | Low | High | Low | High |
| (Ozcelik et al. 2011)^48^ | Low | Some concerns | Some concerns | Low | Low | Some concerns |
| (Pietruska et al. 2018)^49^ | Low | Some concerns | Low | Low | Low | Some concerns |
| (Santamaria et al. 2022)^50^ | Low | Some concerns | Low | Low | Low | Some concerns |
| (Potey et al. 2019)^51^ | Some concerns | Some concerns | Low | Low | Low | Some concerns |
| (Ahmedbeyli et al. 2019)^52^ | Low | Some concerns | Low | Low | Low | Some concerns |
| (Chen et al. 2023)^53^ | Low | Some concerns | Low | Low | Low | Some concerns |
| (Cairo et al. 2016)^54^ | Low | Some concerns | Low | Low | Low | Some concerns |
| (Tonetti et al. 2018)^55^ | Low | Some concerns | Low | Low | Low | Some concerns |
| (Pelekos et al. 2019)^56^ | Some concerns | High | Low | Some concerns | Low | Some concerns |
| (Tonetti et al. 2021)^57^ | Low | Some concerns | Low | Low | Low | Some concerns |

1) Domain 1. Randomization process: allocation sequence was considered random when coin tossing was used; when not specified in the text. allocation concealment was rated as “No Information”. (NI). leading to a risk of bias judgment of “some concerns”; a small number of differences identified as ‘statistically significant’ (p > 0.05) were considered compatible with chance. thus not leading to a risk of bias

2) Domain 2. Deviation from intended intervention: when not otherwise specified and according to the study design and intervention used. participants were considered aware of their assigned intervention; intention-to-treat (ITT) analyses were considered appropriate. analyses excluding eligible trial participants post-randomization were considered inappropriate. leading to a risk of bias judgment of “some concerns”.

3) Domain 3. Missing outcome data: the availability of data from 95% of the participants was considered sufficient.

4) Domain 4. Measurement of the outcome: when the blinding of the assessor was not specified. the risk of bias was rated as “some concerns”.

5) Domain 5. selection of the reported results: all reported results for the outcome measurements were considered in line with the intended analyses.

**Table S3.** The individual assessment of the risk of bias for Professionally-determined Aesthetic Outcome

**Gonzalez-Febles 2023^30^**

| **Domain** | **Signalling question** | | | **Response** | **Comments** |
| --- | --- | --- | --- | --- | --- |
| **Bias arising from the randomization process** | 1.1 Was the allocation sequence random? | | | Y | Subjects were randomized using a block randomization list (in blocks of 4 stratified by study centre) |
|  | 1.2 Was the allocation sequence concealed until participants were enrolled and assigned to interventions? | | | NI |  |
|  | 1.3 Did baseline differences between intervention groups suggest a problem with the randomization process? | | | N |  |
|  | **Risk of bias judgement** | | | **Low** |  |
| **Bias due to deviations from intended interventions** | 2.1.Were participants aware of their assigned intervention during the trial? | | | Y |  |
|  | 2.2.Were carers and people delivering the interventions aware of participants' assigned intervention during the trial? | | | Y |  |
|  | 2.3. If Y/PY/NI to 2.1 or 2.2: Were there deviations from the intended intervention that arose because of the experimental context? | | | N |  |
|  | 2.4 If Y/PY to 2.3: Were these deviations likely to have affected the outcome? | | | NA |  |
|  | 2.5. If Y/PY/NI to 2.4: Were these deviations from intended intervention balanced between groups? | | | NA |  |
|  | 2.6 Was an appropriate analysis used to estimate the effect of assignment to intervention? | | | Y | Intention-to-treat principle was applied |
|  | 2.7 If N/PN/NI to 2.6: Was there potential for a substantial impact (on the result) of the failure to analyse participants in the group to which they were randomized? | | | NA |  |
|  | **Risk of bias judgement** | | | **Low** |  |
| **Bias due to missing outcome data** | 3.1 Were data for this outcome available for all. or nearly all. participants randomized? | | | Y |  |
|  | 3.2 If N/PN/NI to 3.1: Is there evidence that result was not biased by missing outcome data? | | | NA |  |
|  | 3.3 If N/PN to 3.2: Could missingness in the outcome depend on its true value? | | | NA |  |
|  | 3.4 If Y/PY/NI to 3.3: Is it likely that missingness in the outcome depended on its true value? | | | NA |  |
|  | **Risk of bias judgement** | | | **Low** |  |
| **Bias in measurement of the outcome** | 4.1 Was the method of measuring the outcome inappropriate? | | | N | RES at 6 months (5 parameters evaluated and scores of the 5 componets addedd to obtain a total sum score). |
|  | 4.2 Could measurement or ascertainment of the outcome have differed between intervention groups? | | | N |  |
|  | 4.3 Were outcome assessors aware of the intervention received by study participants? | | | Y | The lack of blinding of the outcome assessor is specified in the limitations |
|  | 4.4 If Y/PY/NI to 4.3: Could assessment of the outcome have been influenced by knowledge of intervention received? | | | PY |  |
|  | 4.5 If Y/PY/NI to 4.4: Is it likely that assessment of the outcome was influenced by knowledge of intervention received? | | | PN |  |
|  | **Risk of bias judgement** | | | **Some concerns** |  |
| **Bias in selection of the reported result** | 5.1 Were the data that produced this result analysed in accordance with a pre-specified analysis plan that was finalized before unblinded outcome data were available for analysis? | | | Y |  |
|  | 5.2 ... multiple eligible outcome measurements (e.g. scales. definitions. time points) within the outcome domain? | | | N |  |
|  | 5.3 ... multiple eligible analyses of the data? | | | N |  |
|  | **Risk of bias judgement** | | | **Low** |  |
| **Overall bias** | **Risk of bias judgement** | | | **Some concerns** |  |
|  |  |  |  |  |  |
| **Zangrando 2020^31^** |  |  |  |  |  |
| **Domain** | **Signalling question** | | | **Response** | **Comments** |
| **Bias arising from the randomization process** | 1.1 Was the allocation sequence random? | | | Y | A randomization tool was used to define side and technique. At the time of surgery the envelop was opened. |
|  | 1.2 Was the allocation sequence concealed until participants were enrolled and assigned to interventions? | | | Y |  |
|  | 1.3 Did baseline differences between intervention groups suggest a problem with the randomization process? | | | N |  |
|  | **Risk of bias judgement** | | | **Low** |  |
| **Bias due to deviations from intended interventions** | 2.1.Were participants aware of their assigned intervention during the trial? | | | NI |  |
|  | 2.2.Were carers and people delivering the interventions aware of participants' assigned intervention during the trial? | | | Y |  |
|  | 2.3. If Y/PY/NI to 2.1 or 2.2: Were there deviations from the intended intervention that arose because of the experimental context? | | | N |  |
|  | 2.4 If Y/PY to 2.3: Were these deviations likely to have affected the outcome? | | | NA |  |
|  | 2.5. If Y/PY/NI to 2.4: Were these deviations from intended intervention balanced between groups? | | | NA |  |
|  | 2.6 Was an appropriate analysis used to estimate the effect of assignment to intervention? | | | NI |  |
|  | 2.7 If N/PN/NI to 2.6: Was there potential for a substantial impact (on the result) of the failure to analyse participants in the group to which they were randomized? | | | N |  |
|  | **Risk of bias judgement** | | | **Some concerns** |  |
| **Bias due to missing outcome data** | 3.1 Were data for this outcome available for all. or nearly all. participants randomized? | | | N |  |
|  | 3.2 If N/PN/NI to 3.1: Is there evidence that result was not biased by missing outcome data? | | | N |  |
|  | 3.3 If N/PN to 3.2: Could missingness in the outcome depend on its true value? | | | PN |  |
|  | 3.4 If Y/PY/NI to 3.3: Is it likely that missingness in the outcome depended on its true value? | | | NA |  |
|  | **Risk of bias judgement** | | | **Low** |  |
| **Bias in measurement of the outcome** | 4.1 Was the method of measuring the outcome inappropriate? | | | N | RES at 6 months. |
|  | 4.2 Could measurement or ascertainment of the outcome have differed between intervention groups? | | | N |  |
|  | 4.3 Were outcome assessors aware of the intervention received by study participants? | | | NI | Not specified if the esthetic outcome was evaluated by a blinded assessor |
|  | 4.4 If Y/PY/NI to 4.3: Could assessment of the outcome have been influenced by knowledge of intervention received? | | | NI |  |
|  | 4.5 If Y/PY/NI to 4.4: Is it likely that assessment of the outcome was influenced by knowledge of intervention received? | | | PN |  |
|  | **Risk of bias judgement** | | | **Some concerns** |  |
| **Bias in selection of the reported result** | 5.1 Were the data that produced this result analysed in accordance with a pre-specified analysis plan that was finalized before unblinded outcome data were available for analysis? | | | Y |  |
|  | 5.2 ... multiple eligible outcome measurements (e.g. scales. definitions. time points) within the outcome domain? | | | N |  |
|  | 5.3 ... multiple eligible analyses of the data? | | | N |  |
|  | **Risk of bias judgement** | | | **Low** |  |
| **Overall bias** | **Risk of bias judgement** | | | **Some concerns** |  |
|  |  |  |  |  |  |
| **Rotundo 2021^32^** |  |  |  |  |  |
| **Domain** | **Signalling question** | | | **Response** | **Comments** |
| **Bias arising from the randomization process** | 1.1 Was the allocation sequence random? | | | Y | Absence of detailed information oin the generation of the sequence. but mention to the sealed envelopes opened before surgery. |
|  | 1.2 Was the allocation sequence concealed until participants were enrolled and assigned to interventions? | | | Y |  |
|  | 1.3 Did baseline differences between intervention groups suggest a problem with the randomization process? | | | N |  |
|  | **Risk of bias judgement** | | | **Low** |  |
| **Bias due to deviations from intended interventions** | 2.1.Were participants aware of their assigned intervention during the trial? | | | NI |  |
|  | 2.2.Were carers and people delivering the interventions aware of participants' assigned intervention during the trial? | | | Y |  |
|  | 2.3. If Y/PY/NI to 2.1 or 2.2: Were there deviations from the intended intervention that arose because of the experimental context? | | | N |  |
|  | 2.4 If Y/PY to 2.3: Were these deviations likely to have affected the outcome? | | | NA |  |
|  | 2.5. If Y/PY/NI to 2.4: Were these deviations from intended intervention balanced between groups? | | | NA |  |
|  | 2.6 Was an appropriate analysis used to estimate the effect of assignment to intervention? | | | NI |  |
|  | 2.7 If N/PN/NI to 2.6: Was there potential for a substantial impact (on the result) of the failure to analyse participants in the group to which they were randomized? | | | N |  |
|  | **Risk of bias judgement** | | | **Some concerns** |  |
| **Bias due to missing outcome data** | 3.1 Were data for this outcome available for all. or nearly all. participants randomized? | | | Y |  |
|  | 3.2 If N/PN/NI to 3.1: Is there evidence that result was not biased by missing outcome data? | | | NA |  |
|  | 3.3 If N/PN to 3.2: Could missingness in the outcome depend on its true value? | | | NA |  |
|  | 3.4 If Y/PY/NI to 3.3: Is it likely that missingness in the outcome depended on its true value? | | | NA |  |
|  | **Risk of bias judgement** | | | **Low** |  |
| **Bias in measurement of the outcome** | 4.1 Was the method of measuring the outcome inappropriate? | | | N | SEI by an independent examiner |
|  | 4.2 Could measurement or ascertainment of the outcome have differed between intervention groups? | | | N |  |
|  | 4.3 Were outcome assessors aware of the intervention received by study participants? | | | N |  |
|  | 4.4 If Y/PY/NI to 4.3: Could assessment of the outcome have been influenced by knowledge of intervention received? | | | NA |  |
|  | 4.5 If Y/PY/NI to 4.4: Is it likely that assessment of the outcome was influenced by knowledge of intervention received? | | | NA |  |
|  | **Risk of bias judgement** | | | **Low** |  |
| **Bias in selection of the reported result** | 5.1 Were the data that produced this result analysed in accordance with a pre-specified analysis plan that was finalized before unblinded outcome data were available for analysis? | | | Y |  |
|  | 5.2 ... multiple eligible outcome measurements (e.g. scales. definitions. time points) within the outcome domain? | | | N |  |
|  | 5.3 ... multiple eligible analyses of the data? | | | N |  |
|  | **Risk of bias judgement** | | | **Low** |  |
| **Overall bias** | **Risk of bias judgement** | | | **Some concerns** |  |
|  |  |  |  |  |  |
| **Rakasevic 2020^33^** |  |  |  |  |  |
| **Domain** | **Signalling question** | | | **Response** | **Comments** |
| **Bias arising from the randomization process** | 1.1 Was the allocation sequence random? | | | Y |  |
|  | 1.2 Was the allocation sequence concealed until participants were enrolled and assigned to interventions? | | | Y |  |
|  | 1.3 Did baseline differences between intervention groups suggest a problem with the randomization process? | | | N |  |
|  | **Risk of bias judgement** | | | **Low** |  |
| **Bias due to deviations from intended interventions** | 2.1.Were participants aware of their assigned intervention during the trial? | | | NI |  |
|  | 2.2.Were carers and people delivering the interventions aware of participants' assigned intervention during the trial? | | | Y |  |
|  | 2.3. If Y/PY/NI to 2.1 or 2.2: Were there deviations from the intended intervention that arose because of the experimental context? | | | N |  |
|  | 2.4 If Y/PY to 2.3: Were these deviations likely to have affected the outcome? | | | NA |  |
|  | 2.5. If Y/PY/NI to 2.4: Were these deviations from intended intervention balanced between groups? | | | NA |  |
|  | 2.6 Was an appropriate analysis used to estimate the effect of assignment to intervention? | | | NI |  |
|  | 2.7 If N/PN/NI to 2.6: Was there potential for a substantial impact (on the result) of the failure to analyse participants in the group to which they were randomized? | | | PN |  |
|  | **Risk of bias judgement** | | | **Some concerns** |  |
| **Bias due to missing outcome data** | 3.1 Were data for this outcome available for all. or nearly all. participants randomized? | | | Y |  |
|  | 3.2 If N/PN/NI to 3.1: Is there evidence that result was not biased by missing outcome data? | | | NA |  |
|  | 3.3 If N/PN to 3.2: Could missingness in the outcome depend on its true value? | | | NA |  |
|  | 3.4 If Y/PY/NI to 3.3: Is it likely that missingness in the outcome depended on its true value? | | | NA |  |
|  | **Risk of bias judgement** | | | **Low** |  |
| **Bias in measurement of the outcome** | 4.1 Was the method of measuring the outcome inappropriate? | | | N |  |
|  | 4.2 Could measurement or ascertainment of the outcome have differed between intervention groups? | | | N |  |
|  | 4.3 Were outcome assessors aware of the intervention received by study participants? | | | N |  |
|  | 4.4 If Y/PY/NI to 4.3: Could assessment of the outcome have been influenced by knowledge of intervention received? | | | NA |  |
|  | 4.5 If Y/PY/NI to 4.4: Is it likely that assessment of the outcome was influenced by knowledge of intervention received? | | | NA |  |
|  | **Risk of bias judgement** | | | **Low** |  |
| **Bias in selection of the reported result** | 5.1 Were the data that produced this result analysed in accordance with a pre-specified analysis plan that was finalized before unblinded outcome data were available for analysis? | | | Y |  |
|  | 5.2 ... multiple eligible outcome measurements (e.g. scales. definitions. time points) within the outcome domain? | | | N |  |
|  | 5.3 ... multiple eligible analyses of the data? | | | N |  |
|  | **Risk of bias judgement** | | | **Low** |  |
| **Overall bias** | **Risk of bias judgement** | | | **Some concerns** |  |
|  |  |  |  |  |  |
| **Lakshmi 2023^1^** |  |  |  |  |  |
| **Domain** | **Signalling question** | | | **Response** | **Comments** |
| **Bias arising from the randomization process** | 1.1 Was the allocation sequence random? | | | Y | Allocation concealment was done using a computer generated sequence sealed in opaque envelopes. Opened after tunnel preparation |
|  | 1.2 Was the allocation sequence concealed until participants were enrolled and assigned to interventions? | | | Y |  |
|  | 1.3 Did baseline differences between intervention groups suggest a problem with the randomization process? | | | N |  |
|  | **Risk of bias judgement** | | | **Low** |  |
| **Bias due to deviations from intended interventions** | 2.1.Were participants aware of their assigned intervention during the trial? | | | NI |  |
|  | 2.2.Were carers and people delivering the interventions aware of participants' assigned intervention during the trial? | | | Y |  |
|  | 2.3. If Y/PY/NI to 2.1 or 2.2: Were there deviations from the intended intervention that arose because of the experimental context? | | | N |  |
|  | 2.4 If Y/PY to 2.3: Were these deviations likely to have affected the outcome? | | | NA |  |
|  | 2.5. If Y/PY/NI to 2.4: Were these deviations from intended intervention balanced between groups? | | | NA |  |
|  | 2.6 Was an appropriate analysis used to estimate the effect of assignment to intervention? | | | NI |  |
|  | 2.7 If N/PN/NI to 2.6: Was there potential for a substantial impact (on the result) of the failure to analyse participants in the group to which they were randomized? | | | PN |  |
|  | **Risk of bias judgement** | | | **Some concerns** |  |
| **Bias due to missing outcome data** | 3.1 Were data for this outcome available for all. or nearly all. participants randomized? | | | Y |  |
|  | 3.2 If N/PN/NI to 3.1: Is there evidence that result was not biased by missing outcome data? | | | NA |  |
|  | 3.3 If N/PN to 3.2: Could missingness in the outcome depend on its true value? | | | NA |  |
|  | 3.4 If Y/PY/NI to 3.3: Is it likely that missingness in the outcome depended on its true value? | | | NA |  |
|  | **Risk of bias judgement** | | | **Low** |  |
| **Bias in measurement of the outcome** | 4.1 Was the method of measuring the outcome inappropriate? | | | PN | Not thoroughly described |
|  | 4.2 Could measurement or ascertainment of the outcome have differed between intervention groups? | | | PN |  |
|  | 4.3 Were outcome assessors aware of the intervention received by study participants? | | | N |  |
|  | 4.4 If Y/PY/NI to 4.3: Could assessment of the outcome have been influenced by knowledge of intervention received? | | | NA |  |
|  | 4.5 If Y/PY/NI to 4.4: Is it likely that assessment of the outcome was influenced by knowledge of intervention received? | | | NA |  |
|  | **Risk of bias judgement** | | | **Low** |  |
| **Bias in selection of the reported result** | 5.1 Were the data that produced this result analysed in accordance with a pre-specified analysis plan that was finalized before unblinded outcome data were available for analysis? | | | Y |  |
|  | 5.2 ... multiple eligible outcome measurements (e.g. scales. definitions. time points) within the outcome domain? | | | N |  |
|  | 5.3 ... multiple eligible analyses of the data? | | | N |  |
|  | **Risk of bias judgement** | | | **Low** |  |
| **Overall bias** | **Risk of bias judgement** | | | **Some concerns** |  |
|  |  |  |  |  |  |
| **Carrera 2023^34^** |  |  |  |  |  |
| **Domain** | **Signalling question** | | | **Response** | **Comments** |
| **Bias arising from the randomization process** | 1.1 Was the allocation sequence random? | | | PY | Coin tossing. on the day of surgery. immediately before the procedure. |
|  | 1.2 Was the allocation sequence concealed until participants were enrolled and assigned to interventions? | | | Y |  |
|  | 1.3 Did baseline differences between intervention groups suggest a problem with the randomization process? | | | N |  |
|  | **Risk of bias judgement** | | | **Low** |  |
| **Bias due to deviations from intended interventions** | 2.1.Were participants aware of their assigned intervention during the trial? | | | NI |  |
|  | 2.2.Were carers and people delivering the interventions aware of participants' assigned intervention during the trial? | | | Y |  |
|  | 2.3. If Y/PY/NI to 2.1 or 2.2: Were there deviations from the intended intervention that arose because of the experimental context? | | | N |  |
|  | 2.4 If Y/PY to 2.3: Were these deviations likely to have affected the outcome? | | | NA |  |
|  | 2.5. If Y/PY/NI to 2.4: Were these deviations from intended intervention balanced between groups? | | | NA |  |
|  | 2.6 Was an appropriate analysis used to estimate the effect of assignment to intervention? | | | NI |  |
|  | 2.7 If N/PN/NI to 2.6: Was there potential for a substantial impact (on the result) of the failure to analyse participants in the group to which they were randomized? | | | PN |  |
|  | **Risk of bias judgement** | | | **Some concerns** |  |
| **Bias due to missing outcome data** | 3.1 Were data for this outcome available for all. or nearly all. participants randomized? | | | Y |  |
|  | 3.2 If N/PN/NI to 3.1: Is there evidence that result was not biased by missing outcome data? | | | NA |  |
|  | 3.3 If N/PN to 3.2: Could missingness in the outcome depend on its true value? | | | NA |  |
|  | 3.4 If Y/PY/NI to 3.3: Is it likely that missingness in the outcome depended on its true value? | | | NA |  |
|  | **Risk of bias judgement** | | | **Low** |  |
| **Bias in measurement of the outcome** | 4.1 Was the method of measuring the outcome inappropriate? | | | N | RES. VAS and Questionnaire |
|  | 4.2 Could measurement or ascertainment of the outcome have differed between intervention groups? | | | PN |  |
|  | 4.3 Were outcome assessors aware of the intervention received by study participants? | | | N |  |
|  | 4.4 If Y/PY/NI to 4.3: Could assessment of the outcome have been influenced by knowledge of intervention received? | | | NA |  |
|  | 4.5 If Y/PY/NI to 4.4: Is it likely that assessment of the outcome was influenced by knowledge of intervention received? | | | NA |  |
|  | **Risk of bias judgement** | | | **Low** |  |
| **Bias in selection of the reported result** | 5.1 Were the data that produced this result analysed in accordance with a pre-specified analysis plan that was finalized before unblinded outcome data were available for analysis? | | | Y |  |
|  | 5.2 ... multiple eligible outcome measurements (e.g. scales. definitions. time points) within the outcome domain? | | | N |  |
|  | 5.3 ... multiple eligible analyses of the data? | | | N |  |
|  | **Risk of bias judgement** | | | **Low** |  |
| **Overall bias** | **Risk of bias judgement** | | | **Some concerns** |  |
|  |  |  |  |  |  |
| **Bakhishov 2021^35^** |  |  |  |  |  |
| **Domain** | **Signalling question** | | | **Response** | **Comments** |
| **Bias arising from the randomization process** | 1.1 Was the allocation sequence random? | | | Y | Permuted block design used. Envelopes opened immediately after preparation of the recipient sites |
|  | 1.2 Was the allocation sequence concealed until participants were enrolled and assigned to interventions? | | | Y |  |
|  | 1.3 Did baseline differences between intervention groups suggest a problem with the randomization process? | | | N |  |
|  | **Risk of bias judgement** | | | **Low** |  |
| **Bias due to deviations from intended interventions** | 2.1.Were participants aware of their assigned intervention during the trial? | | | Y | Single blinded |
|  | 2.2.Were carers and people delivering the interventions aware of participants' assigned intervention during the trial? | | | Y |  |
|  | 2.3. If Y/PY/NI to 2.1 or 2.2: Were there deviations from the intended intervention that arose because of the experimental context? | | | N |  |
|  | 2.4 If Y/PY to 2.3: Were these deviations likely to have affected the outcome? | | | NA |  |
|  | 2.5. If Y/PY/NI to 2.4: Were these deviations from intended intervention balanced between groups? | | | NA |  |
|  | 2.6 Was an appropriate analysis used to estimate the effect of assignment to intervention? | | | NI | No ITT and exclusion from the analysis of 7 dropouts |
|  | 2.7 If N/PN/NI to 2.6: Was there potential for a substantial impact (on the result) of the failure to analyse participants in the group to which they were randomized? | | | PY | 7 patients are 20% of the sample |
|  | **Risk of bias judgement** | | | **High** |  |
| **Bias due to missing outcome data** | 3.1 Were data for this outcome available for all. or nearly all. participants randomized? | | | N | Data are not available for all participants randomized |
|  | 3.2 If N/PN/NI to 3.1: Is there evidence that result was not biased by missing outcome data? | | | N |  |
|  | 3.3 If N/PN to 3.2: Could missingness in the outcome depend on its true value? | | | NI |  |
|  | 3.4 If Y/PY/NI to 3.3: Is it likely that missingness in the outcome depended on its true value? | | | NI |  |
|  | **Risk of bias judgement** | | | **High** |  |
| **Bias in measurement of the outcome** | 4.1 Was the method of measuring the outcome inappropriate? | | | N |  |
|  | 4.2 Could measurement or ascertainment of the outcome have differed between intervention groups? | | | N |  |
|  | 4.3 Were outcome assessors aware of the intervention received by study participants? | | | NI |  |
|  | 4.4 If Y/PY/NI to 4.3: Could assessment of the outcome have been influenced by knowledge of intervention received? | | | NI |  |
|  | 4.5 If Y/PY/NI to 4.4: Is it likely that assessment of the outcome was influenced by knowledge of intervention received? | | | PN |  |
|  | **Risk of bias judgement** | | | **Some concerns** |  |
| **Bias in selection of the reported result** | 5.1 Were the data that produced this result analysed in accordance with a pre-specified analysis plan that was finalized before unblinded outcome data were available for analysis? | | | Y |  |
|  | 5.2 ... multiple eligible outcome measurements (e.g. scales. definitions. time points) within the outcome domain? | | | N |  |
|  | 5.3 ... multiple eligible analyses of the data? | | | N |  |
|  | **Risk of bias judgement** | | | **Low** |  |
| **Overall bias** | **Risk of bias judgement** | | | **High** |  |
|  |  |  |  |  |  |
| **Meza-Mauricio 2021^36^** |  |  |  |  |  |
| **Domain** | **Signalling question** | | | **Response** | **Comments** |
| **Bias arising from the randomization process** | 1.1 Was the allocation sequence random? | | | Y | Randomization and allocation concealment was guaranteed by an operator not involved in the clinical trial. Envelopes were opened during surgery after flap elevation |
|  | 1.2 Was the allocation sequence concealed until participants were enrolled and assigned to interventions? | | | Y |  |
|  | 1.3 Did baseline differences between intervention groups suggest a problem with the randomization process? | | | N |  |
|  | **Risk of bias judgement** | | | **Low** |  |
| **Bias due to deviations from intended interventions** | 2.1.Were participants aware of their assigned intervention during the trial? | | | PY |  |
|  | 2.2.Were carers and people delivering the interventions aware of participants' assigned intervention during the trial? | | | Y |  |
|  | 2.3. If Y/PY/NI to 2.1 or 2.2: Were there deviations from the intended intervention that arose because of the experimental context? | | | PN |  |
|  | 2.4 If Y/PY to 2.3: Were these deviations likely to have affected the outcome? | | | NA |  |
|  | 2.5. If Y/PY/NI to 2.4: Were these deviations from intended intervention balanced between groups? | | | NA |  |
|  | 2.6 Was an appropriate analysis used to estimate the effect of assignment to intervention? | | | NI | No mention to ITT |
|  | 2.7 If N/PN/NI to 2.6: Was there potential for a substantial impact (on the result) of the failure to analyse participants in the group to which they were randomized? | | | PN |  |
|  | **Risk of bias judgement** | | | **Some concerns** |  |
| **Bias due to missing outcome data** | 3.1 Were data for this outcome available for all. or nearly all. participants randomized? | | | N | 1 patient lost to follow up after receiving allocated intervention |
|  | 3.2 If N/PN/NI to 3.1: Is there evidence that result was not biased by missing outcome data? | | | PN |  |
|  | 3.3 If N/PN to 3.2: Could missingness in the outcome depend on its true value? | | | N |  |
|  | 3.4 If Y/PY/NI to 3.3: Is it likely that missingness in the outcome depended on its true value? | | | NA |  |
|  | **Risk of bias judgement** | | | **Low** |  |
| **Bias in measurement of the outcome** | 4.1 Was the method of measuring the outcome inappropriate? | | | N | RES evaluated by to masked and calibrated examiners. VAS |
|  | 4.2 Could measurement or ascertainment of the outcome have differed between intervention groups? | | | N |  |
|  | 4.3 Were outcome assessors aware of the intervention received by study participants? | | | N |  |
|  | 4.4 If Y/PY/NI to 4.3: Could assessment of the outcome have been influenced by knowledge of intervention received? | | | NA |  |
|  | 4.5 If Y/PY/NI to 4.4: Is it likely that assessment of the outcome was influenced by knowledge of intervention received? | | | NA |  |
|  | **Risk of bias judgement** | | | **Low** |  |
| **Bias in selection of the reported result** | 5.1 Were the data that produced this result analysed in accordance with a pre-specified analysis plan that was finalized before unblinded outcome data were available for analysis? | | | Y |  |
|  | 5.2 ... multiple eligible outcome measurements (e.g. scales. definitions. time points) within the outcome domain? | | | N |  |
|  | 5.3 ... multiple eligible analyses of the data? | | | N |  |
|  | **Risk of bias judgement** | | | **Low** |  |
| **Overall bias** | **Risk of bias judgement** | | | **Some concerns** |  |
|  |  |  |  |  |  |
| **Gorski 2022^37^** |  |  |  |  |  |
| **Domain** | **Signalling question** | | | **Response** | **Comments** |
| **Bias arising from the randomization process** | 1.1 Was the allocation sequence random? | | | Y | Computerized random number generator used. Allocation of treatment sites concelaed in opaque envelopes and releaved to the surgeon before the procedure |
|  | 1.2 Was the allocation sequence concealed until participants were enrolled and assigned to interventions? | | | Y |  |
|  | 1.3 Did baseline differences between intervention groups suggest a problem with the randomization process? | | | N |  |
|  | **Risk of bias judgement** | | | **Low** |  |
| **Bias due to deviations from intended interventions** | 2.1.Were participants aware of their assigned intervention during the trial? | | | N |  |
|  | 2.2.Were carers and people delivering the interventions aware of participants' assigned intervention during the trial? | | | Y |  |
|  | 2.3. If Y/PY/NI to 2.1 or 2.2: Were there deviations from the intended intervention that arose because of the experimental context? | | | PN |  |
|  | 2.4 If Y/PY to 2.3: Were these deviations likely to have affected the outcome? | | | NA |  |
|  | 2.5. If Y/PY/NI to 2.4: Were these deviations from intended intervention balanced between groups? | | | NA |  |
|  | 2.6 Was an appropriate analysis used to estimate the effect of assignment to intervention? | | | NI | No mention to ITT nor specified how |
|  | 2.7 If N/PN/NI to 2.6: Was there potential for a substantial impact (on the result) of the failure to analyse participants in the group to which they were randomized? | | | PN |  |
|  | **Risk of bias judgement** | | | **Some concerns** |  |
| **Bias due to missing outcome data** | 3.1 Were data for this outcome available for all. or nearly all. participants randomized? | | | N | 1 patient lost to follow up and was excluded from the analysis |
|  | 3.2 If N/PN/NI to 3.1: Is there evidence that result was not biased by missing outcome data? | | | PN | The missing data from the drop out combined with the lack of strategies to handle missing data (itt or imputation) could result in bias |
|  | 3.3 If N/PN to 3.2: Could missingness in the outcome depend on its true value? | | | PN |  |
|  | 3.4 If Y/PY/NI to 3.3: Is it likely that missingness in the outcome depended on its true value? | | | NA |  |
|  | **Risk of bias judgement** | | | **Some concerns** |  |
| **Bias in measurement of the outcome** | 4.1 Was the method of measuring the outcome inappropriate? | | | N | RES by blinded examiner |
|  | 4.2 Could measurement or ascertainment of the outcome have differed between intervention groups? | | | N |  |
|  | 4.3 Were outcome assessors aware of the intervention received by study participants? | | | N |  |
|  | 4.4 If Y/PY/NI to 4.3: Could assessment of the outcome have been influenced by knowledge of intervention received? | | | NA |  |
|  | 4.5 If Y/PY/NI to 4.4: Is it likely that assessment of the outcome was influenced by knowledge of intervention received? | | | NA |  |
|  | **Risk of bias judgement** | | | **Low** |  |
| **Bias in selection of the reported result** | 5.1 Were the data that produced this result analysed in accordance with a pre-specified analysis plan that was finalized before unblinded outcome data were available for analysis? | | | Y |  |
|  | 5.2 ... multiple eligible outcome measurements (e.g. scales. definitions. time points) within the outcome domain? | | | N |  |
|  | 5.3 ... multiple eligible analyses of the data? | | | N |  |
|  | **Risk of bias judgement** | | | **Low** |  |
| **Overall bias** | **Risk of bias judgement** | | | **Some concerns** |  |
|  |  |  |  |  |  |
| **Cieslik-Wegemund 2016^38^** |  |  |  |  |  |
| **Domain** | **Signalling question** | | | **Response** | **Comments** |
| **Bias arising from the randomization process** | 1.1 Was the allocation sequence random? | | | Y | Block randomization method. sequence handed to the surgeon on the day the procedure was performed |
|  | 1.2 Was the allocation sequence concealed until participants were enrolled and assigned to interventions? | | | Y |  |
|  | 1.3 Did baseline differences between intervention groups suggest a problem with the randomization process? | | | N |  |
|  | **Risk of bias judgement** | | | **Low** |  |
| **Bias due to deviations from intended interventions** | 2.1.Were participants aware of their assigned intervention during the trial? | | | NI |  |
|  | 2.2.Were carers and people delivering the interventions aware of participants' assigned intervention during the trial? | | | Y |  |
|  | 2.3. If Y/PY/NI to 2.1 or 2.2: Were there deviations from the intended intervention that arose because of the experimental context? | | | PN |  |
|  | 2.4 If Y/PY to 2.3: Were these deviations likely to have affected the outcome? | | | NA |  |
|  | 2.5. If Y/PY/NI to 2.4: Were these deviations from intended intervention balanced between groups? | | | NA |  |
|  | 2.6 Was an appropriate analysis used to estimate the effect of assignment to intervention? | | | NI | No mention to ITT |
|  | 2.7 If N/PN/NI to 2.6: Was there potential for a substantial impact (on the result) of the failure to analyse participants in the group to which they were randomized? | | | PN |  |
|  | **Risk of bias judgement** | | | **Some concerns** |  |
| **Bias due to missing outcome data** | 3.1 Were data for this outcome available for all. or nearly all. participants randomized? | | | Y | All patients completed the study and attended all of the recall visits |
|  | 3.2 If N/PN/NI to 3.1: Is there evidence that result was not biased by missing outcome data? | | | NA |  |
|  | 3.3 If N/PN to 3.2: Could missingness in the outcome depend on its true value? | | | NA |  |
|  | 3.4 If Y/PY/NI to 3.3: Is it likely that missingness in the outcome depended on its true value? | | | NA |  |
|  | **Risk of bias judgement** | | | **Low** |  |
| **Bias in measurement of the outcome** | 4.1 Was the method of measuring the outcome inappropriate? | | | PN |  |
|  | 4.2 Could measurement or ascertainment of the outcome have differed between intervention groups? | | | N |  |
|  | 4.3 Were outcome assessors aware of the intervention received by study participants? | | | N |  |
|  | 4.4 If Y/PY/NI to 4.3: Could assessment of the outcome have been influenced by knowledge of intervention received? | | | NA |  |
|  | 4.5 If Y/PY/NI to 4.4: Is it likely that assessment of the outcome was influenced by knowledge of intervention received? | | | NA |  |
|  | **Risk of bias judgement** | | | **Low** |  |
| **Bias in selection of the reported result** | 5.1 Were the data that produced this result analysed in accordance with a pre-specified analysis plan that was finalized before unblinded outcome data were available for analysis? | | | Y |  |
|  | 5.2 ... multiple eligible outcome measurements (e.g. scales. definitions. time points) within the outcome domain? | | | N |  |
|  | 5.3 ... multiple eligible analyses of the data? | | | N |  |
|  | **Risk of bias judgement** | | | **Low** |  |
| **Overall bias** | **Risk of bias judgement** | | | **Some concerns** |  |
|  |  |  |  |  |  |
| **Tavelli 2022^39^** |  |  |  |  |  |
| **Domain** | **Signalling question** | | | **Response** | **Comments** |
| **Bias arising from the randomization process** | 1.1 Was the allocation sequence random? | | | Y | Stratified sequential randomization. sealed envelopes |
|  | 1.2 Was the allocation sequence concealed until participants were enrolled and assigned to interventions? | | | Y |  |
|  | 1.3 Did baseline differences between intervention groups suggest a problem with the randomization process? | | | N |  |
|  | **Risk of bias judgement** | | | **Low** |  |
| **Bias due to deviations from intended interventions** | 2.1.Were participants aware of their assigned intervention during the trial? | | | N | The surgeon received a sealed envelop with the patient ID number and a syringe containing a clear solution. either rhPDGF or saline |
|  | 2.2.Were carers and people delivering the interventions aware of participants' assigned intervention during the trial? | | | N |  |
|  | 2.3. If Y/PY/NI to 2.1 or 2.2: Were there deviations from the intended intervention that arose because of the experimental context? | | | NA |  |
|  | 2.4 If Y/PY to 2.3: Were these deviations likely to have affected the outcome? | | | NA |  |
|  | 2.5. If Y/PY/NI to 2.4: Were these deviations from intended intervention balanced between groups? | | | NA |  |
|  | 2.6 Was an appropriate analysis used to estimate the effect of assignment to intervention? | | | NI |  |
|  | 2.7 If N/PN/NI to 2.6: Was there potential for a substantial impact (on the result) of the failure to analyse participants in the group to which they were randomized? | | | PN |  |
|  | **Risk of bias judgement** | | | **Some concerns** |  |
| **Bias due to missing outcome data** | 3.1 Were data for this outcome available for all. or nearly all. participants randomized? | | | Y |  |
|  | 3.2 If N/PN/NI to 3.1: Is there evidence that result was not biased by missing outcome data? | | | NA |  |
|  | 3.3 If N/PN to 3.2: Could missingness in the outcome depend on its true value? | | | NA |  |
|  | 3.4 If Y/PY/NI to 3.3: Is it likely that missingness in the outcome depended on its true value? | | | NA |  |
|  | **Risk of bias judgement** | | | **Low** |  |
| **Bias in measurement of the outcome** | 4.1 Was the method of measuring the outcome inappropriate? | | | N |  |
|  | 4.2 Could measurement or ascertainment of the outcome have differed between intervention groups? | | | N |  |
|  | 4.3 Were outcome assessors aware of the intervention received by study participants? | | | N |  |
|  | 4.4 If Y/PY/NI to 4.3: Could assessment of the outcome have been influenced by knowledge of intervention received? | | | NA |  |
|  | 4.5 If Y/PY/NI to 4.4: Is it likely that assessment of the outcome was influenced by knowledge of intervention received? | | | NA |  |
|  | **Risk of bias judgement** | | | **Low** |  |
| **Bias in selection of the reported result** | 5.1 Were the data that produced this result analysed in accordance with a pre-specified analysis plan that was finalized before unblinded outcome data were available for analysis? | | | Y |  |
|  | 5.2 ... multiple eligible outcome measurements (e.g. scales. definitions. time points) within the outcome domain? | | | N |  |
|  | 5.3 ... multiple eligible analyses of the data? | | | N |  |
|  | **Risk of bias judgement** | | | **Low** |  |
| **Overall bias** | **Risk of bias judgement** | | | **Some concerns** |  |
|  |  |  |  |  |  |
| **Zucchelli 2014^40^** |  |  |  |  |  |
| **Domain** | **Signalling question** | | | **Response** | **Comments** |
| **Bias arising from the randomization process** | 1.1 Was the allocation sequence random? | | | Y | Computer generated sequence. envelopes opened before surgery |
|  | 1.2 Was the allocation sequence concealed until participants were enrolled and assigned to interventions? | | | Y |  |
|  | 1.3 Did baseline differences between intervention groups suggest a problem with the randomization process? | | | N |  |
|  | **Risk of bias judgement** | | | **Low** |  |
| **Bias due to deviations from intended interventions** | 2.1.Were participants aware of their assigned intervention during the trial? | | | PY |  |
|  | 2.2.Were carers and people delivering the interventions aware of participants' assigned intervention during the trial? | | | Y |  |
|  | 2.3. If Y/PY/NI to 2.1 or 2.2: Were there deviations from the intended intervention that arose because of the experimental context? | | | PN |  |
|  | 2.4 If Y/PY to 2.3: Were these deviations likely to have affected the outcome? | | | NA |  |
|  | 2.5. If Y/PY/NI to 2.4: Were these deviations from intended intervention balanced between groups? | | | NA |  |
|  | 2.6 Was an appropriate analysis used to estimate the effect of assignment to intervention? | | | NI | No ITT |
|  | 2.7 If N/PN/NI to 2.6: Was there potential for a substantial impact (on the result) of the failure to analyse participants in the group to which they were randomized? | | | PN |  |
|  | **Risk of bias judgement** | | | **Some concerns** |  |
| **Bias due to missing outcome data** | 3.1 Were data for this outcome available for all. or nearly all. participants randomized? | | | Y | No patients were lost to follow up |
|  | 3.2 If N/PN/NI to 3.1: Is there evidence that result was not biased by missing outcome data? | | | NA |  |
|  | 3.3 If N/PN to 3.2: Could missingness in the outcome depend on its true value? | | | NA |  |
|  | 3.4 If Y/PY/NI to 3.3: Is it likely that missingness in the outcome depended on its true value? | | | NA |  |
|  | **Risk of bias judgement** | | | **Low** |  |
| **Bias in measurement of the outcome** | 4.1 Was the method of measuring the outcome inappropriate? | | | N |  |
|  | 4.2 Could measurement or ascertainment of the outcome have differed between intervention groups? | | | PN |  |
|  | 4.3 Were outcome assessors aware of the intervention received by study participants? | | | N |  |
|  | 4.4 If Y/PY/NI to 4.3: Could assessment of the outcome have been influenced by knowledge of intervention received? | | | NA |  |
|  | 4.5 If Y/PY/NI to 4.4: Is it likely that assessment of the outcome was influenced by knowledge of intervention received? | | | NA |  |
|  | **Risk of bias judgement** | | | **Low** |  |
| **Bias in selection of the reported result** | 5.1 Were the data that produced this result analysed in accordance with a pre-specified analysis plan that was finalized before unblinded outcome data were available for analysis? | | | Y |  |
|  | 5.2 ... multiple eligible outcome measurements (e.g. scales. definitions. time points) within the outcome domain? | | | N |  |
|  | 5.3 ... multiple eligible analyses of the data? | | | N |  |
|  | **Risk of bias judgement** | | | **Low** |  |
| **Overall bias** | **Risk of bias judgement** | | | **Some concerns** |  |
|  |  |  |  |  |  |
| **Gorski 2023^41^** |  |  |  |  |  |
| **Domain** | **Signalling question** | | | **Response** | **Comments** |
| **Bias arising from the randomization process** | 1.1 Was the allocation sequence random? | | | Y |  |
|  | 1.2 Was the allocation sequence concealed until participants were enrolled and assigned to interventions? | | | Y |  |
|  | 1.3 Did baseline differences between intervention groups suggest a problem with the randomization process? | | | N |  |
|  | **Risk of bias judgement** | | | **Low** |  |
| **Bias due to deviations from intended interventions** | 2.1.Were participants aware of their assigned intervention during the trial? | | | NI |  |
|  | 2.2.Were carers and people delivering the interventions aware of participants' assigned intervention during the trial? | | | Y |  |
|  | 2.3. If Y/PY/NI to 2.1 or 2.2: Were there deviations from the intended intervention that arose because of the experimental context? | | | PN |  |
|  | 2.4 If Y/PY to 2.3: Were these deviations likely to have affected the outcome? | | | NA |  |
|  | 2.5. If Y/PY/NI to 2.4: Were these deviations from intended intervention balanced between groups? | | | NA |  |
|  | 2.6 Was an appropriate analysis used to estimate the effect of assignment to intervention? | | | NI |  |
|  | 2.7 If N/PN/NI to 2.6: Was there potential for a substantial impact (on the result) of the failure to analyse participants in the group to which they were randomized? | | | PN |  |
|  | **Risk of bias judgement** | | | **Some concerns** |  |
| **Bias due to missing outcome data** | 3.1 Were data for this outcome available for all. or nearly all. participants randomized? | | | Y |  |
|  | 3.2 If N/PN/NI to 3.1: Is there evidence that result was not biased by missing outcome data? | | | NA |  |
|  | 3.3 If N/PN to 3.2: Could missingness in the outcome depend on its true value? | | | NA |  |
|  | 3.4 If Y/PY/NI to 3.3: Is it likely that missingness in the outcome depended on its true value? | | | NA |  |
|  | **Risk of bias judgement** | | | **Low** |  |
| **Bias in measurement of the outcome** | 4.1 Was the method of measuring the outcome inappropriate? | | | N |  |
|  | 4.2 Could measurement or ascertainment of the outcome have differed between intervention groups? | | | PN |  |
|  | 4.3 Were outcome assessors aware of the intervention received by study participants? | | | N |  |
|  | 4.4 If Y/PY/NI to 4.3: Could assessment of the outcome have been influenced by knowledge of intervention received? | | | NA |  |
|  | 4.5 If Y/PY/NI to 4.4: Is it likely that assessment of the outcome was influenced by knowledge of intervention received? | | | NA |  |
|  | **Risk of bias judgement** | | | **Low** |  |
| **Bias in selection of the reported result** | 5.1 Were the data that produced this result analysed in accordance with a pre-specified analysis plan that was finalized before unblinded outcome data were available for analysis? | | | Y |  |
|  | 5.2 ... multiple eligible outcome measurements (e.g. scales. definitions. time points) within the outcome domain? | | | N |  |
|  | 5.3 ... multiple eligible analyses of the data? | | | N |  |
|  | **Risk of bias judgement** | | | **Low** |  |
| **Overall bias** | **Risk of bias judgement** | | | **Some concerns** |  |
|  |  |  |  |  |  |
| **Gorski 2020^42^** |  |  |  |  |  |
| **Domain** | **Signalling question** | | | **Response** | **Comments** |
| **Bias arising from the randomization process** | 1.1 Was the allocation sequence random? | | | Y | Random sequence. envelopes opened immediately before the procedure |
|  | 1.2 Was the allocation sequence concealed until participants were enrolled and assigned to interventions? | | | Y |  |
|  | 1.3 Did baseline differences between intervention groups suggest a problem with the randomization process? | | | N |  |
|  | **Risk of bias judgement** | | | **Low** |  |
| **Bias due to deviations from intended interventions** | 2.1.Were participants aware of their assigned intervention during the trial? | | | PN |  |
|  | 2.2.Were carers and people delivering the interventions aware of participants' assigned intervention during the trial? | | | Y |  |
|  | 2.3. If Y/PY/NI to 2.1 or 2.2: Were there deviations from the intended intervention that arose because of the experimental context? | | | N |  |
|  | 2.4 If Y/PY to 2.3: Were these deviations likely to have affected the outcome? | | | NA |  |
|  | 2.5. If Y/PY/NI to 2.4: Were these deviations from intended intervention balanced between groups? | | | NA |  |
|  | 2.6 Was an appropriate analysis used to estimate the effect of assignment to intervention? | | | NI |  |
|  | 2.7 If N/PN/NI to 2.6: Was there potential for a substantial impact (on the result) of the failure to analyse participants in the group to which they were randomized? | | | PN |  |
|  | **Risk of bias judgement** | | | **Some concerns** |  |
| **Bias due to missing outcome data** | 3.1 Were data for this outcome available for all. or nearly all. participants randomized? | | | Y |  |
|  | 3.2 If N/PN/NI to 3.1: Is there evidence that result was not biased by missing outcome data? | | | NA |  |
|  | 3.3 If N/PN to 3.2: Could missingness in the outcome depend on its true value? | | | NA |  |
|  | 3.4 If Y/PY/NI to 3.3: Is it likely that missingness in the outcome depended on its true value? | | | NA |  |
|  | **Risk of bias judgement** | | | **Low** |  |
| **Bias in measurement of the outcome** | 4.1 Was the method of measuring the outcome inappropriate? | | | N |  |
|  | 4.2 Could measurement or ascertainment of the outcome have differed between intervention groups? | | | PN |  |
|  | 4.3 Were outcome assessors aware of the intervention received by study participants? | | | N |  |
|  | 4.4 If Y/PY/NI to 4.3: Could assessment of the outcome have been influenced by knowledge of intervention received? | | | NA |  |
|  | 4.5 If Y/PY/NI to 4.4: Is it likely that assessment of the outcome was influenced by knowledge of intervention received? | | | NA |  |
|  | **Risk of bias judgement** | | | **Low** |  |
| **Bias in selection of the reported result** | 5.1 Were the data that produced this result analysed in accordance with a pre-specified analysis plan that was finalized before unblinded outcome data were available for analysis? | | | Y |  |
|  | 5.2 ... multiple eligible outcome measurements (e.g. scales. definitions. time points) within the outcome domain? | | | N |  |
|  | 5.3 ... multiple eligible analyses of the data? | | | N |  |
|  | **Risk of bias judgement** | | | **Low** |  |
| **Overall bias** | **Risk of bias judgement** | | | **Some concerns** |  |
|  |  |  |  |  |  |
|  |  |  |  |  |  |
| **Unique ID** | 1.20 | **Study ID** | 20 | **Assessor** | idr |
| **Ref or Label** | Zucchelli 2009 | **Aim** | assignment to intervention (the 'intention-to-treat' effect) |  |  |
| **Experimental** | ECAF | **Comparator** | VRIsCAF | **Source** | Journal article(s) |
| **Outcome** | Professionally determined aestethic scores | **Results** | Table 2 | **Weight** | 1 |
| **Domain** | **Signalling question** | | | **Response** | **Comments** |
| **Bias arising from the randomization process** | 1.1 Was the allocation sequence random? | | | Y |  |
|  | 1.2 Was the allocation sequence concealed until participants were enrolled and assigned to interventions? | | | Y |  |
|  | 1.3 Did baseline differences between intervention groups suggest a problem with the randomization process? | | | N |  |
|  | **Risk of bias judgement** | | | **Low** |  |
| **Bias due to deviations from intended interventions** | 2.1.Were participants aware of their assigned intervention during the trial? | | | NI |  |
|  | 2.2.Were carers and people delivering the interventions aware of participants' assigned intervention during the trial? | | | Y |  |
|  | 2.3. If Y/PY/NI to 2.1 or 2.2: Were there deviations from the intended intervention that arose because of the experimental context? | | | PN |  |
|  | 2.4 If Y/PY to 2.3: Were these deviations likely to have affected the outcome? | | | NA |  |
|  | 2.5. If Y/PY/NI to 2.4: Were these deviations from intended intervention balanced between groups? | | | NA |  |
|  | 2.6 Was an appropriate analysis used to estimate the effect of assignment to intervention? | | | NI | No ITT |
|  | 2.7 If N/PN/NI to 2.6: Was there potential for a substantial impact (on the result) of the failure to analyse participants in the group to which they were randomized? | | | PN |  |
|  | **Risk of bias judgement** | | | **Some concerns** |  |
| **Bias due to missing outcome data** | 3.1 Were data for this outcome available for all. or nearly all. participants randomized? | | | Y |  |
|  | 3.2 If N/PN/NI to 3.1: Is there evidence that result was not biased by missing outcome data? | | | NA |  |
|  | 3.3 If N/PN to 3.2: Could missingness in the outcome depend on its true value? | | | NA |  |
|  | 3.4 If Y/PY/NI to 3.3: Is it likely that missingness in the outcome depended on its true value? | | | NA |  |
|  | **Risk of bias judgement** | | | **Low** |  |
| **Bias in measurement of the outcome** | 4.1 Was the method of measuring the outcome inappropriate? | | | N |  |
|  | 4.2 Could measurement or ascertainment of the outcome have differed between intervention groups? | | | N |  |
|  | 4.3 Were outcome assessors aware of the intervention received by study participants? | | | N |  |
|  | 4.4 If Y/PY/NI to 4.3: Could assessment of the outcome have been influenced by knowledge of intervention received? | | | NA |  |
|  | 4.5 If Y/PY/NI to 4.4: Is it likely that assessment of the outcome was influenced by knowledge of intervention received? | | | NA |  |
|  | **Risk of bias judgement** | | | **Low** |  |
| **Bias in selection of the reported result** | 5.1 Were the data that produced this result analysed in accordance with a pre-specified analysis plan that was finalized before unblinded outcome data were available for analysis? | | | Y |  |
|  | 5.2 ... multiple eligible outcome measurements (e.g. scales. definitions. time points) within the outcome domain? | | | N |  |
|  | 5.3 ... multiple eligible analyses of the data? | | | N |  |
|  | **Risk of bias judgement** | | | **Low** |  |
| **Overall bias** | **Risk of bias judgement** | | | **Some concerns** |  |
|  |  |  |  |  |  |
| **Nahas 2020^44^** |  |  |  |  |  |
| **Domain** | **Signalling question** | | | **Response** | **Comments** |
| **Bias arising from the randomization process** | 1.1 Was the allocation sequence random? | | | PY |  |
|  | 1.2 Was the allocation sequence concealed until participants were enrolled and assigned to interventions? | | | Y |  |
|  | 1.3 Did baseline differences between intervention groups suggest a problem with the randomization process? | | | N |  |
|  | **Risk of bias judgement** | | | **Low** |  |
| **Bias due to deviations from intended interventions** | 2.1.Were participants aware of their assigned intervention during the trial? | | | Y |  |
|  | 2.2.Were carers and people delivering the interventions aware of participants' assigned intervention during the trial? | | | Y |  |
|  | 2.3. If Y/PY/NI to 2.1 or 2.2: Were there deviations from the intended intervention that arose because of the experimental context? | | | PN |  |
|  | 2.4 If Y/PY to 2.3: Were these deviations likely to have affected the outcome? | | | NA |  |
|  | 2.5. If Y/PY/NI to 2.4: Were these deviations from intended intervention balanced between groups? | | | NA |  |
|  | 2.6 Was an appropriate analysis used to estimate the effect of assignment to intervention? | | | NI |  |
|  | 2.7 If N/PN/NI to 2.6: Was there potential for a substantial impact (on the result) of the failure to analyse participants in the group to which they were randomized? | | | PN |  |
|  | **Risk of bias judgement** | | | **Some concerns** |  |
| **Bias due to missing outcome data** | 3.1 Were data for this outcome available for all. or nearly all. participants randomized? | | | Y |  |
|  | 3.2 If N/PN/NI to 3.1: Is there evidence that result was not biased by missing outcome data? | | | NA |  |
|  | 3.3 If N/PN to 3.2: Could missingness in the outcome depend on its true value? | | | NA |  |
|  | 3.4 If Y/PY/NI to 3.3: Is it likely that missingness in the outcome depended on its true value? | | | NA |  |
|  | **Risk of bias judgement** | | | **Low** |  |
| **Bias in measurement of the outcome** | 4.1 Was the method of measuring the outcome inappropriate? | | | N |  |
|  | 4.2 Could measurement or ascertainment of the outcome have differed between intervention groups? | | | PN |  |
|  | 4.3 Were outcome assessors aware of the intervention received by study participants? | | | N |  |
|  | 4.4 If Y/PY/NI to 4.3: Could assessment of the outcome have been influenced by knowledge of intervention received? | | | NA |  |
|  | 4.5 If Y/PY/NI to 4.4: Is it likely that assessment of the outcome was influenced by knowledge of intervention received? | | | NA |  |
|  | **Risk of bias judgement** | | | **Low** |  |
| **Bias in selection of the reported result** | 5.1 Were the data that produced this result analysed in accordance with a pre-specified analysis plan that was finalized before unblinded outcome data were available for analysis? | | | Y |  |
|  | 5.2 ... multiple eligible outcome measurements (e.g. scales. definitions. time points) within the outcome domain? | | | N |  |
|  | 5.3 ... multiple eligible analyses of the data? | | | N |  |
|  | **Risk of bias judgement** | | | **Low** |  |
| **Overall bias** | **Risk of bias judgement** | | | **Some concerns** |  |
|  |  |  |  |  |  |
| **Kuka 2018^45^** |  |  |  |  |  |
| **Domain** | **Signalling question** | | | **Response** | **Comments** |
| **Bias arising from the randomization process** | 1.1 Was the allocation sequence random? | | | Y | Coin toss before surgery |
|  | 1.2 Was the allocation sequence concealed until participants were enrolled and assigned to interventions? | | | Y |  |
|  | 1.3 Did baseline differences between intervention groups suggest a problem with the randomization process? | | | N |  |
|  | **Risk of bias judgement** | | | **Low** |  |
| **Bias due to deviations from intended interventions** | 2.1.Were participants aware of their assigned intervention during the trial? | | | Y |  |
|  | 2.2.Were carers and people delivering the interventions aware of participants' assigned intervention during the trial? | | | Y |  |
|  | 2.3. If Y/PY/NI to 2.1 or 2.2: Were there deviations from the intended intervention that arose because of the experimental context? | | | PN |  |
|  | 2.4 If Y/PY to 2.3: Were these deviations likely to have affected the outcome? | | | NA |  |
|  | 2.5. If Y/PY/NI to 2.4: Were these deviations from intended intervention balanced between groups? | | | NA |  |
|  | 2.6 Was an appropriate analysis used to estimate the effect of assignment to intervention? | | | NI |  |
|  | 2.7 If N/PN/NI to 2.6: Was there potential for a substantial impact (on the result) of the failure to analyse participants in the group to which they were randomized? | | | N |  |
|  | **Risk of bias judgement** | | | **Some concerns** |  |
| **Bias due to missing outcome data** | 3.1 Were data for this outcome available for all. or nearly all. participants randomized? | | | Y |  |
|  | 3.2 If N/PN/NI to 3.1: Is there evidence that result was not biased by missing outcome data? | | | NA |  |
|  | 3.3 If N/PN to 3.2: Could missingness in the outcome depend on its true value? | | | NA |  |
|  | 3.4 If Y/PY/NI to 3.3: Is it likely that missingness in the outcome depended on its true value? | | | NA |  |
|  | **Risk of bias judgement** | | | **Low** |  |
| **Bias in measurement of the outcome** | 4.1 Was the method of measuring the outcome inappropriate? | | | N |  |
|  | 4.2 Could measurement or ascertainment of the outcome have differed between intervention groups? | | | PN |  |
|  | 4.3 Were outcome assessors aware of the intervention received by study participants? | | | N |  |
|  | 4.4 If Y/PY/NI to 4.3: Could assessment of the outcome have been influenced by knowledge of intervention received? | | | NA |  |
|  | 4.5 If Y/PY/NI to 4.4: Is it likely that assessment of the outcome was influenced by knowledge of intervention received? | | | NA |  |
|  | **Risk of bias judgement** | | | **Low** |  |
| **Bias in selection of the reported result** | 5.1 Were the data that produced this result analysed in accordance with a pre-specified analysis plan that was finalized before unblinded outcome data were available for analysis? | | | Y |  |
|  | 5.2 ... multiple eligible outcome measurements (e.g. scales. definitions. time points) within the outcome domain? | | | N |  |
|  | 5.3 ... multiple eligible analyses of the data? | | | N |  |
|  | **Risk of bias judgement** | | | **Low** |  |
| **Overall bias** | **Risk of bias judgement** | | | **Some concerns** |  |
|  |  |  |  |  |  |
| **Trivedi 2023^46^** |  |  |  |  |  |
| **Domain** | **Signalling question** | | | **Response** | **Comments** |
| **Bias arising from the randomization process** | 1.1 Was the allocation sequence random? | | | Y |  |
|  | 1.2 Was the allocation sequence concealed until participants were enrolled and assigned to interventions? | | | Y |  |
|  | 1.3 Did baseline differences between intervention groups suggest a problem with the randomization process? | | | N |  |
|  | **Risk of bias judgement** | | | **Low** |  |
| **Bias due to deviations from intended interventions** | 2.1.Were participants aware of their assigned intervention during the trial? | | | N |  |
|  | 2.2.Were carers and people delivering the interventions aware of participants' assigned intervention during the trial? | | | Y |  |
|  | 2.3. If Y/PY/NI to 2.1 or 2.2: Were there deviations from the intended intervention that arose because of the experimental context? | | | PN |  |
|  | 2.4 If Y/PY to 2.3: Were these deviations likely to have affected the outcome? | | | NA |  |
|  | 2.5. If Y/PY/NI to 2.4: Were these deviations from intended intervention balanced between groups? | | | NA |  |
|  | 2.6 Was an appropriate analysis used to estimate the effect of assignment to intervention? | | | NI |  |
|  | 2.7 If N/PN/NI to 2.6: Was there potential for a substantial impact (on the result) of the failure to analyse participants in the group to which they were randomized? | | | PN |  |
|  | **Risk of bias judgement** | | | **Some concerns** |  |
| **Bias due to missing outcome data** | 3.1 Were data for this outcome available for all. or nearly all. participants randomized? | | | Y |  |
|  | 3.2 If N/PN/NI to 3.1: Is there evidence that result was not biased by missing outcome data? | | | NA |  |
|  | 3.3 If N/PN to 3.2: Could missingness in the outcome depend on its true value? | | | NA |  |
|  | 3.4 If Y/PY/NI to 3.3: Is it likely that missingness in the outcome depended on its true value? | | | NA |  |
|  | **Risk of bias judgement** | | | **Low** |  |
| **Bias in measurement of the outcome** | 4.1 Was the method of measuring the outcome inappropriate? | | | N |  |
|  | 4.2 Could measurement or ascertainment of the outcome have differed between intervention groups? | | | PN |  |
|  | 4.3 Were outcome assessors aware of the intervention received by study participants? | | | N |  |
|  | 4.4 If Y/PY/NI to 4.3: Could assessment of the outcome have been influenced by knowledge of intervention received? | | | NA |  |
|  | 4.5 If Y/PY/NI to 4.4: Is it likely that assessment of the outcome was influenced by knowledge of intervention received? | | | NA |  |
|  | **Risk of bias judgement** | | | **Low** |  |
| **Bias in selection of the reported result** | 5.1 Were the data that produced this result analysed in accordance with a pre-specified analysis plan that was finalized before unblinded outcome data were available for analysis? | | | Y |  |
|  | 5.2 ... multiple eligible outcome measurements (e.g. scales. definitions. time points) within the outcome domain? | | | N |  |
|  | 5.3 ... multiple eligible analyses of the data? | | | N |  |
|  | **Risk of bias judgement** | | | **Low** |  |
| **Overall bias** | **Risk of bias judgement** | | | **Some concerns** |  |
|  |  |  |  |  |  |
| **Ahmedbeyli 2014^47^** |  |  |  |  |  |
| **Domain** | **Signalling question** | | | **Response** | **Comments** |
| **Bias arising from the randomization process** | 1.1 Was the allocation sequence random? | | | Y |  |
|  | 1.2 Was the allocation sequence concealed until participants were enrolled and assigned to interventions? | | | NI |  |
|  | 1.3 Did baseline differences between intervention groups suggest a problem with the randomization process? | | | N |  |
|  | **Risk of bias judgement** | | | **Some concerns** |  |
| **Bias due to deviations from intended interventions** | 2.1.Were participants aware of their assigned intervention during the trial? | | | NI |  |
|  | 2.2.Were carers and people delivering the interventions aware of participants' assigned intervention during the trial? | | | NI |  |
|  | 2.3. If Y/PY/NI to 2.1 or 2.2: Were there deviations from the intended intervention that arose because of the experimental context? | | | NI |  |
|  | 2.4 If Y/PY to 2.3: Were these deviations likely to have affected the outcome? | | | NA |  |
|  | 2.5. If Y/PY/NI to 2.4: Were these deviations from intended intervention balanced between groups? | | | NA |  |
|  | 2.6 Was an appropriate analysis used to estimate the effect of assignment to intervention? | | | NI |  |
|  | 2.7 If N/PN/NI to 2.6: Was there potential for a substantial impact (on the result) of the failure to analyse participants in the group to which they were randomized? | | | N |  |
|  | **Risk of bias judgement** | | | **Some concerns** |  |
| **Bias due to missing outcome data** | 3.1 Were data for this outcome available for all. or nearly all. participants randomized? | | | Y |  |
|  | 3.2 If N/PN/NI to 3.1: Is there evidence that result was not biased by missing outcome data? | | | NA |  |
|  | 3.3 If N/PN to 3.2: Could missingness in the outcome depend on its true value? | | | NA |  |
|  | 3.4 If Y/PY/NI to 3.3: Is it likely that missingness in the outcome depended on its true value? | | | NA |  |
|  | **Risk of bias judgement** | | | **Low** |  |
| **Bias in measurement of the outcome** | 4.1 Was the method of measuring the outcome inappropriate? | | | PN |  |
|  | 4.2 Could measurement or ascertainment of the outcome have differed between intervention groups? | | | NI |  |
|  | 4.3 Were outcome assessors aware of the intervention received by study participants? | | | NI |  |
|  | 4.4 If Y/PY/NI to 4.3: Could assessment of the outcome have been influenced by knowledge of intervention received? | | | NI |  |
|  | 4.5 If Y/PY/NI to 4.4: Is it likely that assessment of the outcome was influenced by knowledge of intervention received? | | | NI |  |
|  | **Risk of bias judgement** | | | **High** |  |
| **Bias in selection of the reported result** | 5.1 Were the data that produced this result analysed in accordance with a pre-specified analysis plan that was finalized before unblinded outcome data were available for analysis? | | | Y |  |
|  | 5.2 ... multiple eligible outcome measurements (e.g. scales. definitions. time points) within the outcome domain? | | | N |  |
|  | 5.3 ... multiple eligible analyses of the data? | | | N |  |
|  | **Risk of bias judgement** | | | **Low** |  |
| **Overall bias** | **Risk of bias judgement** | | | **High** |  |
| **Ozcelik 2011^48^** |  |  |  |  |  |
|  |  |  |  |  |  |
| **Domain** | **Signalling question** | | | **Response** | **Comments** |
| **Bias arising from the randomization process** | 1.1 Was the allocation sequence random? | | | Y | Coin toss. Envelopes opened at time of surgery |
|  | 1.2 Was the allocation sequence concealed until participants were enrolled and assigned to interventions? | | | Y |  |
|  | 1.3 Did baseline differences between intervention groups suggest a problem with the randomization process? | | | N |  |
|  | **Risk of bias judgement** | | | **Low** |  |
| **Bias due to deviations from intended interventions** | 2.1.Were participants aware of their assigned intervention during the trial? | | | PY |  |
|  | 2.2.Were carers and people delivering the interventions aware of participants' assigned intervention during the trial? | | | Y |  |
|  | 2.3. If Y/PY/NI to 2.1 or 2.2: Were there deviations from the intended intervention that arose because of the experimental context? | | | PN |  |
|  | 2.4 If Y/PY to 2.3: Were these deviations likely to have affected the outcome? | | | NA |  |
|  | 2.5. If Y/PY/NI to 2.4: Were these deviations from intended intervention balanced between groups? | | | NA |  |
|  | 2.6 Was an appropriate analysis used to estimate the effect of assignment to intervention? | | | NI |  |
|  | 2.7 If N/PN/NI to 2.6: Was there potential for a substantial impact (on the result) of the failure to analyse participants in the group to which they were randomized? | | | N |  |
|  | **Risk of bias judgement** | | | **Some concerns** |  |
| **Bias due to missing outcome data** | 3.1 Were data for this outcome available for all. or nearly all. participants randomized? | | | N | 2 patients (one in test and one in control group) were excluded from the study and from the analysis |
|  | 3.2 If N/PN/NI to 3.1: Is there evidence that result was not biased by missing outcome data? | | | PN |  |
|  | 3.3 If N/PN to 3.2: Could missingness in the outcome depend on its true value? | | | N |  |
|  | 3.4 If Y/PY/NI to 3.3: Is it likely that missingness in the outcome depended on its true value? | | | NA |  |
|  | **Risk of bias judgement** | | | **Some concerns** |  |
| **Bias in measurement of the outcome** | 4.1 Was the method of measuring the outcome inappropriate? | | | N |  |
|  | 4.2 Could measurement or ascertainment of the outcome have differed between intervention groups? | | | PN |  |
|  | 4.3 Were outcome assessors aware of the intervention received by study participants? | | | N |  |
|  | 4.4 If Y/PY/NI to 4.3: Could assessment of the outcome have been influenced by knowledge of intervention received? | | | NA |  |
|  | 4.5 If Y/PY/NI to 4.4: Is it likely that assessment of the outcome was influenced by knowledge of intervention received? | | | NA |  |
|  | **Risk of bias judgement** | | | **Low** |  |
| **Bias in selection of the reported result** | 5.1 Were the data that produced this result analysed in accordance with a pre-specified analysis plan that was finalized before unblinded outcome data were available for analysis? | | | Y |  |
|  | 5.2 ... multiple eligible outcome measurements (e.g. scales. definitions. time points) within the outcome domain? | | | N |  |
|  | 5.3 ... multiple eligible analyses of the data? | | | N |  |
|  | **Risk of bias judgement** | | | **Low** |  |
| **Overall bias** | **Risk of bias judgement** | | | **Some concerns** |  |
|  |  |  |  |  |  |
| **Pietruska 2018^49^** |  |  |  |  |  |
| **Domain** | **Signalling question** | | | **Response** | **Comments** |
| **Bias arising from the randomization process** | 1.1 Was the allocation sequence random? | | | Y |  |
|  | 1.2 Was the allocation sequence concealed until participants were enrolled and assigned to interventions? | | | Y |  |
|  | 1.3 Did baseline differences between intervention groups suggest a problem with the randomization process? | | | N |  |
|  | **Risk of bias judgement** | | | **Low** |  |
| **Bias due to deviations from intended interventions** | 2.1.Were participants aware of their assigned intervention during the trial? | | | Y |  |
|  | 2.2.Were carers and people delivering the interventions aware of participants' assigned intervention during the trial? | | | Y |  |
|  | 2.3. If Y/PY/NI to 2.1 or 2.2: Were there deviations from the intended intervention that arose because of the experimental context? | | | PN |  |
|  | 2.4 If Y/PY to 2.3: Were these deviations likely to have affected the outcome? | | | NA |  |
|  | 2.5. If Y/PY/NI to 2.4: Were these deviations from intended intervention balanced between groups? | | | NA |  |
|  | 2.6 Was an appropriate analysis used to estimate the effect of assignment to intervention? | | | NI |  |
|  | 2.7 If N/PN/NI to 2.6: Was there potential for a substantial impact (on the result) of the failure to analyse participants in the group to which they were randomized? | | | PN |  |
|  | **Risk of bias judgement** | | | **Some concerns** |  |
| **Bias due to missing outcome data** | 3.1 Were data for this outcome available for all. or nearly all. participants randomized? | | | Y |  |
|  | 3.2 If N/PN/NI to 3.1: Is there evidence that result was not biased by missing outcome data? | | | NA |  |
|  | 3.3 If N/PN to 3.2: Could missingness in the outcome depend on its true value? | | | NA |  |
|  | 3.4 If Y/PY/NI to 3.3: Is it likely that missingness in the outcome depended on its true value? | | | NA |  |
|  | **Risk of bias judgement** | | | **Low** |  |
| **Bias in measurement of the outcome** | 4.1 Was the method of measuring the outcome inappropriate? | | | N |  |
|  | 4.2 Could measurement or ascertainment of the outcome have differed between intervention groups? | | | PN |  |
|  | 4.3 Were outcome assessors aware of the intervention received by study participants? | | | N |  |
|  | 4.4 If Y/PY/NI to 4.3: Could assessment of the outcome have been influenced by knowledge of intervention received? | | | NA |  |
|  | 4.5 If Y/PY/NI to 4.4: Is it likely that assessment of the outcome was influenced by knowledge of intervention received? | | | NA |  |
|  | **Risk of bias judgement** | | | **Low** |  |
| **Bias in selection of the reported result** | 5.1 Were the data that produced this result analysed in accordance with a pre-specified analysis plan that was finalized before unblinded outcome data were available for analysis? | | | Y |  |
|  | 5.2 ... multiple eligible outcome measurements (e.g. scales. definitions. time points) within the outcome domain? | | | N |  |
|  | 5.3 ... multiple eligible analyses of the data? | | | N |  |
|  | **Risk of bias judgement** | | | **Low** |  |
| **Overall bias** | **Risk of bias judgement** | | | **Some concerns** |  |
|  |  |  |  |  |  |
| **Santamaria 2022^50^** |  |  |  |  |  |
| **Domain** | **Signalling question** | | | **Response** | **Comments** |
| **Bias arising from the randomization process** | 1.1 Was the allocation sequence random? | | | Y |  |
|  | 1.2 Was the allocation sequence concealed until participants were enrolled and assigned to interventions? | | | Y |  |
|  | 1.3 Did baseline differences between intervention groups suggest a problem with the randomization process? | | | N |  |
|  | **Risk of bias judgement** | | | **Low** |  |
| **Bias due to deviations from intended interventions** | 2.1.Were participants aware of their assigned intervention during the trial? | | | Y |  |
|  | 2.2.Were carers and people delivering the interventions aware of participants' assigned intervention during the trial? | | | Y |  |
|  | 2.3. If Y/PY/NI to 2.1 or 2.2: Were there deviations from the intended intervention that arose because of the experimental context? | | | PN |  |
|  | 2.4 If Y/PY to 2.3: Were these deviations likely to have affected the outcome? | | | NA |  |
|  | 2.5. If Y/PY/NI to 2.4: Were these deviations from intended intervention balanced between groups? | | | NA |  |
|  | 2.6 Was an appropriate analysis used to estimate the effect of assignment to intervention? | | | NI |  |
|  | 2.7 If N/PN/NI to 2.6: Was there potential for a substantial impact (on the result) of the failure to analyse participants in the group to which they were randomized? | | | PN |  |
|  | **Risk of bias judgement** | | | **Some concerns** |  |
| **Bias due to missing outcome data** | 3.1 Were data for this outcome available for all. or nearly all. participants randomized? | | | Y |  |
|  | 3.2 If N/PN/NI to 3.1: Is there evidence that result was not biased by missing outcome data? | | | NA |  |
|  | 3.3 If N/PN to 3.2: Could missingness in the outcome depend on its true value? | | | NA |  |
|  | 3.4 If Y/PY/NI to 3.3: Is it likely that missingness in the outcome depended on its true value? | | | NA |  |
|  | **Risk of bias judgement** | | | **Low** |  |
| **Bias in measurement of the outcome** | 4.1 Was the method of measuring the outcome inappropriate? | | | N |  |
|  | 4.2 Could measurement or ascertainment of the outcome have differed between intervention groups? | | | PN |  |
|  | 4.3 Were outcome assessors aware of the intervention received by study participants? | | | N |  |
|  | 4.4 If Y/PY/NI to 4.3: Could assessment of the outcome have been influenced by knowledge of intervention received? | | | NA |  |
|  | 4.5 If Y/PY/NI to 4.4: Is it likely that assessment of the outcome was influenced by knowledge of intervention received? | | | NA |  |
|  | **Risk of bias judgement** | | | **Low** |  |
| **Bias in selection of the reported result** | 5.1 Were the data that produced this result analysed in accordance with a pre-specified analysis plan that was finalized before unblinded outcome data were available for analysis? | | | Y |  |
|  | 5.2 ... multiple eligible outcome measurements (e.g. scales. definitions. time points) within the outcome domain? | | | N |  |
|  | 5.3 ... multiple eligible analyses of the data? | | | N |  |
|  | **Risk of bias judgement** | | | **Low** |  |
| **Overall bias** | **Risk of bias judgement** | | | **Some concerns** |  |
|  |  |  |  |  |  |
| **Potey 2019^51^** |  |  |  |  |  |
| **Domain** | **Signalling question** | | | **Response** | **Comments** |
| **Bias arising from the randomization process** | 1.1 Was the allocation sequence random? | | | Y |  |
|  | 1.2 Was the allocation sequence concealed until participants were enrolled and assigned to interventions? | | | NI |  |
|  | 1.3 Did baseline differences between intervention groups suggest a problem with the randomization process? | | | N |  |
|  | **Risk of bias judgement** | | | **Some concerns** |  |
| **Bias due to deviations from intended interventions** | 2.1.Were participants aware of their assigned intervention during the trial? | | | PY |  |
|  | 2.2.Were carers and people delivering the interventions aware of participants' assigned intervention during the trial? | | | Y |  |
|  | 2.3. If Y/PY/NI to 2.1 or 2.2: Were there deviations from the intended intervention that arose because of the experimental context? | | | PN |  |
|  | 2.4 If Y/PY to 2.3: Were these deviations likely to have affected the outcome? | | | NA |  |
|  | 2.5. If Y/PY/NI to 2.4: Were these deviations from intended intervention balanced between groups? | | | NA |  |
|  | 2.6 Was an appropriate analysis used to estimate the effect of assignment to intervention? | | | NI |  |
|  | 2.7 If N/PN/NI to 2.6: Was there potential for a substantial impact (on the result) of the failure to analyse participants in the group to which they were randomized? | | | N |  |
|  | **Risk of bias judgement** | | | **Some concerns** |  |
| **Bias due to missing outcome data** | 3.1 Were data for this outcome available for all. or nearly all. participants randomized? | | | Y |  |
|  | 3.2 If N/PN/NI to 3.1: Is there evidence that result was not biased by missing outcome data? | | | NA |  |
|  | 3.3 If N/PN to 3.2: Could missingness in the outcome depend on its true value? | | | NA |  |
|  | 3.4 If Y/PY/NI to 3.3: Is it likely that missingness in the outcome depended on its true value? | | | NA |  |
|  | **Risk of bias judgement** | | | **Low** |  |
| **Bias in measurement of the outcome** | 4.1 Was the method of measuring the outcome inappropriate? | | | N |  |
|  | 4.2 Could measurement or ascertainment of the outcome have differed between intervention groups? | | | PN |  |
|  | 4.3 Were outcome assessors aware of the intervention received by study participants? | | | PY |  |
|  | 4.4 If Y/PY/NI to 4.3: Could assessment of the outcome have been influenced by knowledge of intervention received? | | | PN |  |
|  | 4.5 If Y/PY/NI to 4.4: Is it likely that assessment of the outcome was influenced by knowledge of intervention received? | | | NA |  |
|  | **Risk of bias judgement** | | | **Low** |  |
| **Bias in selection of the reported result** | 5.1 Were the data that produced this result analysed in accordance with a pre-specified analysis plan that was finalized before unblinded outcome data were available for analysis? | | | Y |  |
|  | 5.2 ... multiple eligible outcome measurements (e.g. scales. definitions. time points) within the outcome domain? | | | N |  |
|  | 5.3 ... multiple eligible analyses of the data? | | | N |  |
|  | **Risk of bias judgement** | | | **Low** |  |
| **Overall bias** | **Risk of bias judgement** | | | **Some concerns** |  |
|  |  |  |  |  |  |
| **Ahmedbeyli 2019^52^** |  |  |  |  |  |
| **Domain** | **Signalling question** | | | **Response** | **Comments** |
| **Bias arising from the randomization process** | 1.1 Was the allocation sequence random? | | | Y |  |
|  | 1.2 Was the allocation sequence concealed until participants were enrolled and assigned to interventions? | | | Y |  |
|  | 1.3 Did baseline differences between intervention groups suggest a problem with the randomization process? | | | N |  |
|  | **Risk of bias judgement** | | | **Low** |  |
| **Bias due to deviations from intended interventions** | 2.1.Were participants aware of their assigned intervention during the trial? | | | Y |  |
|  | 2.2.Were carers and people delivering the interventions aware of participants' assigned intervention during the trial? | | | Y |  |
|  | 2.3. If Y/PY/NI to 2.1 or 2.2: Were there deviations from the intended intervention that arose because of the experimental context? | | | PN |  |
|  | 2.4 If Y/PY to 2.3: Were these deviations likely to have affected the outcome? | | | NA |  |
|  | 2.5. If Y/PY/NI to 2.4: Were these deviations from intended intervention balanced between groups? | | | NA |  |
|  | 2.6 Was an appropriate analysis used to estimate the effect of assignment to intervention? | | | NI |  |
|  | 2.7 If N/PN/NI to 2.6: Was there potential for a substantial impact (on the result) of the failure to analyse participants in the group to which they were randomized? | | | N |  |
|  | **Risk of bias judgement** | | | **Some concerns** |  |
| **Bias due to missing outcome data** | 3.1 Were data for this outcome available for all. or nearly all. participants randomized? | | | Y |  |
|  | 3.2 If N/PN/NI to 3.1: Is there evidence that result was not biased by missing outcome data? | | | NA |  |
|  | 3.3 If N/PN to 3.2: Could missingness in the outcome depend on its true value? | | | NA |  |
|  | 3.4 If Y/PY/NI to 3.3: Is it likely that missingness in the outcome depended on its true value? | | | NA |  |
|  | **Risk of bias judgement** | | | **Low** |  |
| **Bias in measurement of the outcome** | 4.1 Was the method of measuring the outcome inappropriate? | | | N |  |
|  | 4.2 Could measurement or ascertainment of the outcome have differed between intervention groups? | | | PN |  |
|  | 4.3 Were outcome assessors aware of the intervention received by study participants? | | | N |  |
|  | 4.4 If Y/PY/NI to 4.3: Could assessment of the outcome have been influenced by knowledge of intervention received? | | | NA |  |
|  | 4.5 If Y/PY/NI to 4.4: Is it likely that assessment of the outcome was influenced by knowledge of intervention received? | | | NA |  |
|  | **Risk of bias judgement** | | | **Low** |  |
| **Bias in selection of the reported result** | 5.1 Were the data that produced this result analysed in accordance with a pre-specified analysis plan that was finalized before unblinded outcome data were available for analysis? | | | Y |  |
|  | 5.2 ... multiple eligible outcome measurements (e.g. scales. definitions. time points) within the outcome domain? | | | N |  |
|  | 5.3 ... multiple eligible analyses of the data? | | | N |  |
|  | **Risk of bias judgement** | | | **Low** |  |
| **Overall bias** | **Risk of bias judgement** | | | **Some concerns** |  |
|  |  |  |  |  |  |
| **Chen 2023^53^** |  |  |  |  |  |
| **Domain** | **Signalling question** | | | **Response** | **Comments** |
| **Bias arising from the randomization process** | 1.1 Was the allocation sequence random? | | | Y |  |
|  | 1.2 Was the allocation sequence concealed until participants were enrolled and assigned to interventions? | | | Y |  |
|  | 1.3 Did baseline differences between intervention groups suggest a problem with the randomization process? | | | N |  |
|  | **Risk of bias judgement** | | | **Low** |  |
| **Bias due to deviations from intended interventions** | 2.1.Were participants aware of their assigned intervention during the trial? | | | Y |  |
|  | 2.2.Were carers and people delivering the interventions aware of participants' assigned intervention during the trial? | | | Y |  |
|  | 2.3. If Y/PY/NI to 2.1 or 2.2: Were there deviations from the intended intervention that arose because of the experimental context? | | | PN |  |
|  | 2.4 If Y/PY to 2.3: Were these deviations likely to have affected the outcome? | | | NA |  |
|  | 2.5. If Y/PY/NI to 2.4: Were these deviations from intended intervention balanced between groups? | | | NA |  |
|  | 2.6 Was an appropriate analysis used to estimate the effect of assignment to intervention? | | | NI |  |
|  | 2.7 If N/PN/NI to 2.6: Was there potential for a substantial impact (on the result) of the failure to analyse participants in the group to which they were randomized? | | | PN |  |
|  | **Risk of bias judgement** | | | **Some concerns** |  |
| **Bias due to missing outcome data** | 3.1 Were data for this outcome available for all. or nearly all. participants randomized? | | | Y |  |
|  | 3.2 If N/PN/NI to 3.1: Is there evidence that result was not biased by missing outcome data? | | | NA |  |
|  | 3.3 If N/PN to 3.2: Could missingness in the outcome depend on its true value? | | | NA |  |
|  | 3.4 If Y/PY/NI to 3.3: Is it likely that missingness in the outcome depended on its true value? | | | NA |  |
|  | **Risk of bias judgement** | | | **Low** |  |
| **Bias in measurement of the outcome** | 4.1 Was the method of measuring the outcome inappropriate? | | | N |  |
|  | 4.2 Could measurement or ascertainment of the outcome have differed between intervention groups? | | | PN |  |
|  | 4.3 Were outcome assessors aware of the intervention received by study participants? | | | N |  |
|  | 4.4 If Y/PY/NI to 4.3: Could assessment of the outcome have been influenced by knowledge of intervention received? | | | NA |  |
|  | 4.5 If Y/PY/NI to 4.4: Is it likely that assessment of the outcome was influenced by knowledge of intervention received? | | | NA |  |
|  | **Risk of bias judgement** | | | **Low** |  |
| **Bias in selection of the reported result** | 5.1 Were the data that produced this result analysed in accordance with a pre-specified analysis plan that was finalized before unblinded outcome data were available for analysis? | | | Y |  |
|  | 5.2 ... multiple eligible outcome measurements (e.g. scales. definitions. time points) within the outcome domain? | | | N |  |
|  | 5.3 ... multiple eligible analyses of the data? | | | N |  |
|  | **Risk of bias judgement** | | | **Low** |  |
| **Overall bias** | **Risk of bias judgement** | | | **Some concerns** |  |

**Cairo et al. 2016 ^54^**

| **Domain** | **Signalling question** | **Response** | **Comments** |
| --- | --- | --- | --- |
| **Bias arising from the randomization process** | 1.1 Was the allocation sequence random? | Y | Randomization sealed and opaque envelopes opened after flap elevation |
|  | 1.2 Was the allocation sequence concealed until participants were enrolled and assigned to interventions? | Y |  |
|  | 1.3 Did baseline differences between intervention groups suggest a problem with the randomization process? | PN |  |
|  | **Risk of bias judgement** | **Low** |  |
| **Bias due to deviations from intended interventions** | 2.1.Were participants aware of their assigned intervention during the trial? | Y |  |
|  | 2.2.Were carers and people delivering the interventions aware of participants' assigned intervention during the trial? | Y |  |
|  | 2.3. If Y/PY/NI to 2.1 or 2.2: Were there deviations from the intended intervention that arose because of the experimental context? | PN |  |
|  | 2.4 If Y/PY to 2.3: Were these deviations likely to have affected the outcome? | NA |  |
|  | 2.5. If Y/PY/NI to 2.4: Were these deviations from intended intervention balanced between groups? | NA |  |
|  | 2.6 Was an appropriate analysis used to estimate the effect of assignment to intervention? | PN |  |
|  | 2.7 If N/PN/NI to 2.6: Was there potential for a substantial impact (on the result) of the failure to analyse participants in the group to which they were randomized? | PN |  |
|  | **Risk of bias judgement** | **Some concerns** |  |
| **Bias due to missing outcome data** | 3.1 Were data for this outcome available for all. or nearly all. participants randomized? | Y |  |
|  | 3.2 If N/PN/NI to 3.1: Is there evidence that result was not biased by missing outcome data? | NA |  |
|  | 3.3 If N/PN to 3.2: Could missingness in the outcome depend on its true value? | NA |  |
|  | 3.4 If Y/PY/NI to 3.3: Is it likely that missingness in the outcome depended on its true value? | NA |  |
|  | **Risk of bias judgement** | **Low** |  |
| **Bias in measurement of the outcome** | 4.1 Was the method of measuring the outcome inappropriate? | N |  |
|  | 4.2 Could measurement or ascertainment of the outcome have differed between intervention groups? | PN |  |
|  | 4.3 Were outcome assessors aware of the intervention received by study participants? | N |  |
|  | 4.4 If Y/PY/NI to 4.3: Could assessment of the outcome have been influenced by knowledge of intervention received? | NA |  |
|  | 4.5 If Y/PY/NI to 4.4: Is it likely that assessment of the outcome was influenced by knowledge of intervention received? | NA |  |
|  | **Risk of bias judgement** | **Low** |  |
| **Bias in selection of the reported result** | 5.1 Were the data that produced this result analysed in accordance with a pre-specified analysis plan that was finalized before unblinded outcome data were available for analysis? | Y |  |
|  | 5.2 ... multiple eligible outcome measurements (e.g. scales. definitions. time points) within the outcome domain? | N |  |
|  | 5.3 ... multiple eligible analyses of the data? | N |  |
|  | **Risk of bias judgement** | **Low** |  |
| **Overall bias** | **Risk of bias judgement** | **Some concerns** |  |

**Tonetti et al. 2018 ^58^**

| **Domain** | **Signalling question** | **Response** | **Comments** |
| --- | --- | --- | --- |
| **Bias arising from the randomization process** | 1.1 Was the allocation sequence random? | Y |  |
|  | 1.2 Was the allocation sequence concealed until participants were enrolled and assigned to interventions? | Y |  |
|  | 1.3 Did baseline differences between intervention groups suggest a problem with the randomization process? | PN |  |
|  | **Risk of bias judgement** | **Low** |  |
| **Bias due to deviations from intended interventions** | 2.1.Were participants aware of their assigned intervention during the trial? | Y |  |
|  | 2.2.Were carers and people delivering the interventions aware of participants' assigned intervention during the trial? | Y |  |
|  | 2.3. If Y/PY/NI to 2.1 or 2.2: Were there deviations from the intended intervention that arose because of the experimental context? | PN |  |
|  | 2.4 If Y/PY to 2.3: Were these deviations likely to have affected the outcome? | NA |  |
|  | 2.5. If Y/PY/NI to 2.4: Were these deviations from intended intervention balanced between groups? | NA |  |
|  | 2.6 Was an appropriate analysis used to estimate the effect of assignment to intervention? | PN |  |
|  | 2.7 If N/PN/NI to 2.6: Was there potential for a substantial impact (on the result) of the failure to analyse participants in the group to which they were randomized? | PN |  |
|  | **Risk of bias judgement** | **Some concerns** |  |
| **Bias due to missing outcome data** | 3.1 Were data for this outcome available for all. or nearly all. participants randomized? | Y |  |
|  | 3.2 If N/PN/NI to 3.1: Is there evidence that result was not biased by missing outcome data? | NA |  |
|  | 3.3 If N/PN to 3.2: Could missingness in the outcome depend on its true value? | NA |  |
|  | 3.4 If Y/PY/NI to 3.3: Is it likely that missingness in the outcome depended on its true value? | NA |  |
|  | **Risk of bias judgement** | **Low** |  |
| **Bias in measurement of the outcome** | 4.1 Was the method of measuring the outcome inappropriate? | N |  |
|  | 4.2 Could measurement or ascertainment of the outcome have differed between intervention groups? | PN |  |
|  | 4.3 Were outcome assessors aware of the intervention received by study participants? | N |  |
|  | 4.4 If Y/PY/NI to 4.3: Could assessment of the outcome have been influenced by knowledge of intervention received? | NA |  |
|  | 4.5 If Y/PY/NI to 4.4: Is it likely that assessment of the outcome was influenced by knowledge of intervention received? | NA |  |
|  | **Risk of bias judgement** | **Low** |  |
| **Bias in selection of the reported result** | 5.1 Were the data that produced this result analysed in accordance with a pre-specified analysis plan that was finalized before unblinded outcome data were available for analysis? | Y |  |
|  | 5.2 ... multiple eligible outcome measurements (e.g. scales. definitions. time points) within the outcome domain? | N |  |
|  | 5.3 ... multiple eligible analyses of the data? | N |  |
|  | **Risk of bias judgement** | **Low** |  |
| **Overall bias** | **Risk of bias judgement** | **Some concerns** |  |

**Pelekos et al. 2019^56^**

| **Domain** | **Signalling question** | **Response** | **Comments** |
| --- | --- | --- | --- |
| **Bias arising from the randomization process** | 1.1 Was the allocation sequence random? | Y |  |
|  | 1.2 Was the allocation sequence concealed until participants were enrolled and assigned to interventions? | NI |  |
|  | 1.3 Did baseline differences between intervention groups suggest a problem with the randomization process? | NI |  |
|  | **Risk of bias judgement** | **Some concerns** |  |
| **Bias due to deviations from intended interventions** | 2.1.Were participants aware of their assigned intervention during the trial? | NI |  |
|  | 2.2.Were carers and people delivering the interventions aware of participants' assigned intervention during the trial? | NI |  |
|  | 2.3. If Y/PY/NI to 2.1 or 2.2: Were there deviations from the intended intervention that arose because of the experimental context? | NI |  |
|  | 2.4 If Y/PY to 2.3: Were these deviations likely to have affected the outcome? | NA |  |
|  | 2.5. If Y/PY/NI to 2.4: Were these deviations from intended intervention balanced between groups? | NA |  |
|  | 2.6 Was an appropriate analysis used to estimate the effect of assignment to intervention? | NI |  |
|  | 2.7 If N/PN/NI to 2.6: Was there potential for a substantial impact (on the result) of the failure to analyse participants in the group to which they were randomized? | NI |  |
|  | **Risk of bias judgement** | **High** |  |
| **Bias due to missing outcome data** | 3.1 Were data for this outcome available for all. or nearly all. participants randomized? | Y |  |
|  | 3.2 If N/PN/NI to 3.1: Is there evidence that result was not biased by missing outcome data? | NA |  |
|  | 3.3 If N/PN to 3.2: Could missingness in the outcome depend on its true value? | NA |  |
|  | 3.4 If Y/PY/NI to 3.3: Is it likely that missingness in the outcome depended on its true value? | NA |  |
|  | **Risk of bias judgement** | **Low** |  |
| **Bias in measurement of the outcome** | 4.1 Was the method of measuring the outcome inappropriate? | N |  |
|  | 4.2 Could measurement or ascertainment of the outcome have differed between intervention groups? | NI |  |
|  | 4.3 Were outcome assessors aware of the intervention received by study participants? | N |  |
|  | 4.4 If Y/PY/NI to 4.3: Could assessment of the outcome have been influenced by knowledge of intervention received? | NA |  |
|  | 4.5 If Y/PY/NI to 4.4: Is it likely that assessment of the outcome was influenced by knowledge of intervention received? | NA |  |
|  | **Risk of bias judgement** | **Some concerns** |  |
| **Bias in selection of the reported result** | 5.1 Were the data that produced this result analysed in accordance with a pre-specified analysis plan that was finalized before unblinded outcome data were available for analysis? | Y |  |
|  | 5.2 ... multiple eligible outcome measurements (e.g. scales. definitions. time points) within the outcome domain? | N |  |
|  | 5.3 ... multiple eligible analyses of the data? | N |  |
|  | **Risk of bias judgement** | **Low** |  |
| **Overall bias** | **Risk of bias judgement** | **High** |  |

**Tonetti et al. 2021^57^**

| **Domain** | **Signalling question** | **Response** | **Comments** |
| --- | --- | --- | --- |
| **Bias arising from the randomization process** | 1.1 Was the allocation sequence random? | Y |  |
|  | 1.2 Was the allocation sequence concealed until participants were enrolled and assigned to interventions? | Y |  |
|  | 1.3 Did baseline differences between intervention groups suggest a problem with the randomization process? | PN |  |
|  | **Risk of bias judgement** | **Low** |  |
| **Bias due to deviations from intended interventions** | 2.1.Were participants aware of their assigned intervention during the trial? | Y |  |
|  | 2.2.Were carers and people delivering the interventions aware of participants' assigned intervention during the trial? | Y |  |
|  | 2.3. If Y/PY/NI to 2.1 or 2.2: Were there deviations from the intended intervention that arose because of the experimental context? | PN |  |
|  | 2.4 If Y/PY to 2.3: Were these deviations likely to have affected the outcome? | NA |  |
|  | 2.5. If Y/PY/NI to 2.4: Were these deviations from intended intervention balanced between groups? | NA |  |
|  | 2.6 Was an appropriate analysis used to estimate the effect of assignment to intervention? | PN |  |
|  | 2.7 If N/PN/NI to 2.6: Was there potential for a substantial impact (on the result) of the failure to analyse participants in the group to which they were randomized? | PN |  |
|  | **Risk of bias judgement** | **Some concerns** |  |
| **Bias due to missing outcome data** | 3.1 Were data for this outcome available for all. or nearly all. participants randomized? | Y |  |
|  | 3.2 If N/PN/NI to 3.1: Is there evidence that result was not biased by missing outcome data? | NA |  |
|  | 3.3 If N/PN to 3.2: Could missingness in the outcome depend on its true value? | NA |  |
|  | 3.4 If Y/PY/NI to 3.3: Is it likely that missingness in the outcome depended on its true value? | NA |  |
|  | **Risk of bias judgement** | **Low** |  |
| **Bias in measurement of the outcome** | 4.1 Was the method of measuring the outcome inappropriate? | N |  |
|  | 4.2 Could measurement or ascertainment of the outcome have differed between intervention groups? | PN |  |
|  | 4.3 Were outcome assessors aware of the intervention received by study participants? | N |  |
|  | 4.4 If Y/PY/NI to 4.3: Could assessment of the outcome have been influenced by knowledge of intervention received? | NA |  |
|  | 4.5 If Y/PY/NI to 4.4: Is it likely that assessment of the outcome was influenced by knowledge of intervention received? | NA |  |
|  | **Risk of bias judgement** | **Low** |  |
| **Bias in selection of the reported result** | 5.1 Were the data that produced this result analysed in accordance with a pre-specified analysis plan that was finalized before unblinded outcome data were available for analysis? | Y |  |
|  | 5.2 ... multiple eligible outcome measurements (e.g. scales. definitions. time points) within the outcome domain? | N |  |
|  | 5.3 ... multiple eligible analyses of the data? | N |  |
|  | **Risk of bias judgement** | **Low** |  |
| **Overall bias** | **Risk of bias judgement** | **Some concerns** |  |

**Table S4.** Risk of bias assessment for the included RCTs using The Cochrane Risk of Bias Tool 2 (RoB2) for Randomized Controlled Trials studies (Higgins et al..2023; Sterne et al..2019) for Patient-determined Aesthetic Outcome

| Publication | Randomization process | Deviations from intended interventions | Missing outcome data | Measurement of the outcome | Selection of the reported result | Overall risk of bias |
| --- | --- | --- | --- | --- | --- | --- |
| (Gonzalez-Febles et al. 2023)^30^ | Some concerns | Low | Low | Some concerns | Low | Some concerns |
| (Zangrando et al. 2020)^31^ | Low | Some concerns | Low | Some concerns | Low | Some concerns |
| (Rotundo et al. 2021)^32^ | Low | Some concerns | Low | Low | Low | Some concerns |
| (Carrera et al. 2023)^34^ | Low | Some concerns | Low | Some concerns | Low | Some concerns |
| (Elmahdi et al. 2022)^59^ | Some concerns | Some concerns | Low | Some concerns | Low | Some concerns |
| (Yilmaz et al. 2022)^60^ | Low | High | Some concerns | Some concerns | Low | High |
| (Bakhishov et al. 2020)^35^ | Low | High | High | Some concerns | Low | High |
| (Meza Mauricio et al. 2021)^36^ | Low | Some concerns | Low | Some concerns | Low | Some concerns |
| (Gorski et al. 2022)^37^ | Low | Some concerns | Low | Low | Low | Some concerns |
| (Cieslik et al. 2016)^38^ | Low | Some concerns | Low | Some concerns | Low | Some concerns |
| (Tavelli et al. 2022)^39^ | Low | Some concerns | Low | Low | Low | Some concerns |
| (Aroca et al. 2013)^61^ | Low | Some concerns | Low | Some concerns | Low | Some concerns |
| (Zucchelli et al. 2014)^40^ | Low | Some concerns | Low | Some concerns | Low | Some concerns |
| (Fernandez-Jimenez et al. 2023)^62^ | Low | Some concerns | Low | Low | Low | Some concerns |
| (Gorski et al.2023)^41^ | Low | Some concerns | Low | Low | Low | Some concerns |
| (Gorski et el. 2020)^42^ | Low | Some concerns | Low | Low | Low | Some concerns |
| (Zucchelli et al. 2009)^43^ | Some concerns | Some concerns | Low | Low | Low | Some concerns |
| (Nahas et al. 2020)^44^ | Low | Some concerns | Low | Some concerns | Low | Some concerns |
| (Kuka et al. 2018)^45^ | Low | Some concerns | Low | Some concerns | Low | Some concerns |
| (Trivedi BDS et al. 2023)^46^ | Some concerns | Some concerns | Low | Low | Low | Some concerns |
| (Ahmedbeyli et al. 2014)^47^ | Some concerns | Some concerns | Low | High | Low | High |
| (Ozcelik et al. 2011)^48^ | Low | Some concerns | Low | Some concerns | Low | Some concerns |
| (Rotundo et al. 2019)^63^ | Low | Some concerns | Low | Some concerns | Low | Some concerns |
| (Potey et al. 2019)^51^ | Some concerns | Some concerns | Low | Some concerns | Low | Some concerns |
| (Rajeswari et al. 2021)^64^ | Some concerns | Some concerns | Low | Some concerns | Low | Some concerns |
| (Ahmedbeyli et al. 2019)^52^ | Low | Some concerns | Low | Some concerns | Low | Some concerns |
| (Chen et al. 2023)^53^ | Low | Some concerns | Low | Some concerns | Low | Some concerns |
| (Cairo et al. 2016)^54^ | Low | Some concerns | Low | Low | Low | Some concerns |
| (Tonetti et al. 2018)^55^ | Low | Some concerns | Low | Low | Low | Some concerns |

1) Domain 1. Randomization process: allocation sequence was considered random when coin tossing was used; when not specified in the text. allocation concealment was rated as “No Information”. (NI). leading to a risk of bias judgment of “some concerns”; a small number of differences identified as ‘statistically significant’ (p > 0.05) were considered compatible with chance. thus not leading to a risk of bias

2) Domain 2. Deviation from intended intervention: when not otherwise specified and according to the study design and intervention used. participants were considered aware of their assigned intervention; intention-to-treat (ITT) analyses were considered appropriate. analyses excluding eligible trial participants post-randomization were considered inappropriate. leading to a risk of bias judgment of “some concerns”.

3) Domain 3. Missing outcome data: the availability of data from 95% of the participants was considered sufficient.

4) Domain 4. Measurement of the outcome: being the outcome evaluated a patients-reported outcome measure. and given that for PROMs the outcome assessor is the study participant. if patients were aware of the intervention. the outcome assessment was considered potentially influenced by knowledge of intervention received. leading to a judgment of ‘some concerns’; if blinding was not specified. the decision was based on the intervention used.

5) Domain 5. selection of the reported results: all reported results for the outcome measurements were considered in line with the intended analyses.

**Table S5. Individul Risk of bias assessment for Patient-determined Aesthetic Outcome**

**Gonzalez-Febles 2023^30^**

| **Domain** | **Signalling question** | | | **Response** | **Comments** |
| --- | --- | --- | --- | --- | --- |
| **Bias arising from the randomization process** | 1.1 Was the allocation sequence random? | | | Y | Subjects were randomized using a block randomization list (in blocks of 4 stratified by study centre) |
|  | 1.2 Was the allocation sequence concealed until participants were enrolled and assigned to interventions? | | | NI |  |
|  | 1.3 Did baseline differences between intervention groups suggest a problem with the randomization process? | | | N |  |
|  | **Risk of bias judgement** | | | **Some concerns** |  |
| **Bias due to deviations from intended interventions** | 2.1.Were participants aware of their assigned intervention during the trial? | | | Y |  |
|  | 2.2.Were carers and people delivering the interventions aware of participants' assigned intervention during the trial? | | | Y |  |
|  | 2.3. If Y/PY/NI to 2.1 or 2.2: Were there deviations from the intended intervention that arose because of the experimental context? | | | N |  |
|  | 2.4 If Y/PY to 2.3: Were these deviations likely to have affected the outcome? | | | NA |  |
|  | 2.5. If Y/PY/NI to 2.4: Were these deviations from intended intervention balanced between groups? | | | NA |  |
|  | 2.6 Was an appropriate analysis used to estimate the effect of assignment to intervention? | | | Y | Intention-to-treat principle was applied |
|  | 2.7 If N/PN/NI to 2.6: Was there potential for a substantial impact (on the result) of the failure to analyse participants in the group to which they were randomized? | | | NA |  |
|  | **Risk of bias judgement** | | | **Low** |  |
| **Bias due to missing outcome data** | 3.1 Were data for this outcome available for all. or nearly all. participants randomized? | | | Y |  |
|  | 3.2 If N/PN/NI to 3.1: Is there evidence that result was not biased by missing outcome data? | | | NA |  |
|  | 3.3 If N/PN to 3.2: Could missingness in the outcome depend on its true value? | | | NA |  |
|  | 3.4 If Y/PY/NI to 3.3: Is it likely that missingness in the outcome depended on its true value? | | | NA |  |
|  | **Risk of bias judgement** | | | **Low** |  |
| **Bias in measurement of the outcome** | 4.1 Was the method of measuring the outcome inappropriate? | | | N | PROMs evaluated with VAS. 14 days-diary. number of tablets. OHIP-14 and PREMs. |
|  | 4.2 Could measurement or ascertainment of the outcome have differed between intervention groups? | | | N |  |
|  | 4.3 Were outcome assessors aware of the intervention received by study participants? | | | Y |  |
|  | 4.4 If Y/PY/NI to 4.3: Could assessment of the outcome have been influenced by knowledge of intervention received? | | | NI |  |
|  | 4.5 If Y/PY/NI to 4.4: Is it likely that assessment of the outcome was influenced by knowledge of intervention received? | | | PN |  |
|  | **Risk of bias judgement** | | | **Some concerns** |  |
| **Bias in selection of the reported result** | 5.1 Were the data that produced this result analysed in accordance with a pre-specified analysis plan that was finalized before unblinded outcome data were available for analysis? | | | Y |  |
|  | 5.2 ... multiple eligible outcome measurements (e.g. scales. definitions. time points) within the outcome domain? | | | N |  |
|  | 5.3 ... multiple eligible analyses of the data? | | | N |  |
|  | **Risk of bias judgement** | | | **Low** |  |
| **Overall bias** | **Risk of bias judgement** | | | **Some concerns** |  |
|  |  |  |  |  |  |
| **Zangrando 2020^31^** |  |  |  |  |  |
| **Domain** | **Signalling question** | | | **Response** | **Comments** |
| **Bias arising from the randomization process** | 1.1 Was the allocation sequence random? | | | Y | A randomization tool was used to define side and technique. At the time of surgery the envelop was opened. |
|  | 1.2 Was the allocation sequence concealed until participants were enrolled and assigned to interventions? | | | Y |  |
|  | 1.3 Did baseline differences between intervention groups suggest a problem with the randomization process? | | | N |  |
|  | **Risk of bias judgement** | | | **Low** |  |
| **Bias due to deviations from intended interventions** | 2.1.Were participants aware of their assigned intervention during the trial? | | | NI |  |
|  | 2.2.Were carers and people delivering the interventions aware of participants' assigned intervention during the trial? | | | Y |  |
|  | 2.3. If Y/PY/NI to 2.1 or 2.2: Were there deviations from the intended intervention that arose because of the experimental context? | | | N |  |
|  | 2.4 If Y/PY to 2.3: Were these deviations likely to have affected the outcome? | | | NA |  |
|  | 2.5. If Y/PY/NI to 2.4: Were these deviations from intended intervention balanced between groups? | | | NA |  |
|  | 2.6 Was an appropriate analysis used to estimate the effect of assignment to intervention? | | | NI |  |
|  | 2.7 If N/PN/NI to 2.6: Was there potential for a substantial impact (on the result) of the failure to analyse participants in the group to which they were randomized? | | | N |  |
|  | **Risk of bias judgement** | | | **Some concerns** |  |
| **Bias due to missing outcome data** | 3.1 Were data for this outcome available for all. or nearly all. participants randomized? | | | N |  |
|  | 3.2 If N/PN/NI to 3.1: Is there evidence that result was not biased by missing outcome data? | | | N |  |
|  | 3.3 If N/PN to 3.2: Could missingness in the outcome depend on its true value? | | | PN |  |
|  | 3.4 If Y/PY/NI to 3.3: Is it likely that missingness in the outcome depended on its true value? | | | NA |  |
|  | **Risk of bias judgement** | | | **Low** |  |
| **Bias in measurement of the outcome** | 4.1 Was the method of measuring the outcome inappropriate? | | | N | VAS 7 days post surgey and patients' aesthetic evaluation at 6 months. |
|  | 4.2 Could measurement or ascertainment of the outcome have differed between intervention groups? | | | N |  |
|  | 4.3 Were outcome assessors aware of the intervention received by study participants? | | | NI |  |
|  | 4.4 If Y/PY/NI to 4.3: Could assessment of the outcome have been influenced by knowledge of intervention received? | | | NI |  |
|  | 4.5 If Y/PY/NI to 4.4: Is it likely that assessment of the outcome was influenced by knowledge of intervention received? | | | PN |  |
|  | **Risk of bias judgement** | | | **Some concerns** |  |
| **Bias in selection of the reported result** | 5.1 Were the data that produced this result analysed in accordance with a pre-specified analysis plan that was finalized before unblinded outcome data were available for analysis? | | | Y |  |
|  | 5.2 ... multiple eligible outcome measurements (e.g. scales. definitions. time points) within the outcome domain? | | | N |  |
|  | 5.3 ... multiple eligible analyses of the data? | | | N |  |
|  | **Risk of bias judgement** | | | **Low** |  |
| **Overall bias** | **Risk of bias judgement** | | | **Some concerns** |  |
|  |  |  |  |  |  |
| **Rotundo 2021^32^** |  |  |  |  |  |
| **Domain** | **Signalling question** | | | **Response** | **Comments** |
| **Bias arising from the randomization process** | 1.1 Was the allocation sequence random? | | | Y | Absence of detailed information oin the generation of the sequence. but mention to the sealed envelopes opened before surgery. |
|  | 1.2 Was the allocation sequence concealed until participants were enrolled and assigned to interventions? | | | Y |  |
|  | 1.3 Did baseline differences between intervention groups suggest a problem with the randomization process? | | | N |  |
|  | **Risk of bias judgement** | | | **Low** |  |
| **Bias due to deviations from intended interventions** | 2.1.Were participants aware of their assigned intervention during the trial? | | | PY |  |
|  | 2.2.Were carers and people delivering the interventions aware of participants' assigned intervention during the trial? | | | Y |  |
|  | 2.3. If Y/PY/NI to 2.1 or 2.2: Were there deviations from the intended intervention that arose because of the experimental context? | | | N |  |
|  | 2.4 If Y/PY to 2.3: Were these deviations likely to have affected the outcome? | | | NA |  |
|  | 2.5. If Y/PY/NI to 2.4: Were these deviations from intended intervention balanced between groups? | | | NA |  |
|  | 2.6 Was an appropriate analysis used to estimate the effect of assignment to intervention? | | | NI |  |
|  | 2.7 If N/PN/NI to 2.6: Was there potential for a substantial impact (on the result) of the failure to analyse participants in the group to which they were randomized? | | | N |  |
|  | **Risk of bias judgement** | | | **Some concerns** |  |
| **Bias due to missing outcome data** | 3.1 Were data for this outcome available for all. or nearly all. participants randomized? | | | Y |  |
|  | 3.2 If N/PN/NI to 3.1: Is there evidence that result was not biased by missing outcome data? | | | NA |  |
|  | 3.3 If N/PN to 3.2: Could missingness in the outcome depend on its true value? | | | NA |  |
|  | 3.4 If Y/PY/NI to 3.3: Is it likely that missingness in the outcome depended on its true value? | | | NA |  |
|  | **Risk of bias judgement** | | | **Low** |  |
| **Bias in measurement of the outcome** | 4.1 Was the method of measuring the outcome inappropriate? | | | N | SEI by an independent examiner; VAS by patients |
|  | 4.2 Could measurement or ascertainment of the outcome have differed between intervention groups? | | | N |  |
|  | 4.3 Were outcome assessors aware of the intervention received by study participants? | | | PY |  |
|  | 4.4 If Y/PY/NI to 4.3: Could assessment of the outcome have been influenced by knowledge of intervention received? | | | PN |  |
|  | 4.5 If Y/PY/NI to 4.4: Is it likely that assessment of the outcome was influenced by knowledge of intervention received? | | | NA |  |
|  | **Risk of bias judgement** | | | **Low** |  |
| **Bias in selection of the reported result** | 5.1 Were the data that produced this result analysed in accordance with a pre-specified analysis plan that was finalized before unblinded outcome data were available for analysis? | | | Y |  |
|  | 5.2 ... multiple eligible outcome measurements (e.g. scales. definitions. time points) within the outcome domain? | | | N |  |
|  | 5.3 ... multiple eligible analyses of the data? | | | N |  |
|  | **Risk of bias judgement** | | | **Low** |  |
| **Overall bias** | **Risk of bias judgement** | | | **Some concerns** |  |
|  |  |  |  |  |  |
| **Carrera 2023^34^** |  |  |  |  |  |
| **Domain** | **Signalling question** | | | **Response** | **Comments** |
| **Bias arising from the randomization process** | 1.1 Was the allocation sequence random? | | | PY | Coin tossing. on the day of surgery. immediately before the procedure. |
|  | 1.2 Was the allocation sequence concealed until participants were enrolled and assigned to interventions? | | | Y |  |
|  | 1.3 Did baseline differences between intervention groups suggest a problem with the randomization process? | | | N |  |
|  | **Risk of bias judgement** | | | **Low** |  |
| **Bias due to deviations from intended interventions** | 2.1.Were participants aware of their assigned intervention during the trial? | | | NI |  |
|  | 2.2.Were carers and people delivering the interventions aware of participants' assigned intervention during the trial? | | | Y |  |
|  | 2.3. If Y/PY/NI to 2.1 or 2.2: Were there deviations from the intended intervention that arose because of the experimental context? | | | N |  |
|  | 2.4 If Y/PY to 2.3: Were these deviations likely to have affected the outcome? | | | NA |  |
|  | 2.5. If Y/PY/NI to 2.4: Were these deviations from intended intervention balanced between groups? | | | NA |  |
|  | 2.6 Was an appropriate analysis used to estimate the effect of assignment to intervention? | | | NI |  |
|  | 2.7 If N/PN/NI to 2.6: Was there potential for a substantial impact (on the result) of the failure to analyse participants in the group to which they were randomized? | | | PN |  |
|  | **Risk of bias judgement** | | | **Some concerns** |  |
| **Bias due to missing outcome data** | 3.1 Were data for this outcome available for all. or nearly all. participants randomized? | | | Y |  |
|  | 3.2 If N/PN/NI to 3.1: Is there evidence that result was not biased by missing outcome data? | | | NA |  |
|  | 3.3 If N/PN to 3.2: Could missingness in the outcome depend on its true value? | | | NA |  |
|  | 3.4 If Y/PY/NI to 3.3: Is it likely that missingness in the outcome depended on its true value? | | | NA |  |
|  | **Risk of bias judgement** | | | **Low** |  |
| **Bias in measurement of the outcome** | 4.1 Was the method of measuring the outcome inappropriate? | | | N | RES. VAS and Questionnaire |
|  | 4.2 Could measurement or ascertainment of the outcome have differed between intervention groups? | | | PN |  |
|  | 4.3 Were outcome assessors aware of the intervention received by study participants? | | | PY |  |
|  | 4.4 If Y/PY/NI to 4.3: Could assessment of the outcome have been influenced by knowledge of intervention received? | | | NI |  |
|  | 4.5 If Y/PY/NI to 4.4: Is it likely that assessment of the outcome was influenced by knowledge of intervention received? | | | PN |  |
|  | **Risk of bias judgement** | | | **Some concerns** |  |
| **Bias in selection of the reported result** | 5.1 Were the data that produced this result analysed in accordance with a pre-specified analysis plan that was finalized before unblinded outcome data were available for analysis? | | | Y |  |
|  | 5.2 ... multiple eligible outcome measurements (e.g. scales. definitions. time points) within the outcome domain? | | | N |  |
|  | 5.3 ... multiple eligible analyses of the data? | | | N |  |
|  | **Risk of bias judgement** | | | **Low** |  |
| **Overall bias** | **Risk of bias judgement** | | | **Some concerns** |  |
|  |  |  |  |  |  |
| **Elmahdi 2022^59^** |  |  |  |  |  |
| **Domain** | **Signalling question** | | | **Response** | **Comments** |
| **Bias arising from the randomization process** | 1.1 Was the allocation sequence random? | | | Y |  |
|  | 1.2 Was the allocation sequence concealed until participants were enrolled and assigned to interventions? | | | NI |  |
|  | 1.3 Did baseline differences between intervention groups suggest a problem with the randomization process? | | | PN |  |
|  | **Risk of bias judgement** | | | **Some concerns** |  |
| **Bias due to deviations from intended interventions** | 2.1.Were participants aware of their assigned intervention during the trial? | | | PY |  |
|  | 2.2.Were carers and people delivering the interventions aware of participants' assigned intervention during the trial? | | | Y |  |
|  | 2.3. If Y/PY/NI to 2.1 or 2.2: Were there deviations from the intended intervention that arose because of the experimental context? | | | PN |  |
|  | 2.4 If Y/PY to 2.3: Were these deviations likely to have affected the outcome? | | | NA |  |
|  | 2.5. If Y/PY/NI to 2.4: Were these deviations from intended intervention balanced between groups? | | | NA |  |
|  | 2.6 Was an appropriate analysis used to estimate the effect of assignment to intervention? | | | NI |  |
|  | 2.7 If N/PN/NI to 2.6: Was there potential for a substantial impact (on the result) of the failure to analyse participants in the group to which they were randomized? | | | PN |  |
|  | **Risk of bias judgement** | | | **Some concerns** |  |
| **Bias due to missing outcome data** | 3.1 Were data for this outcome available for all. or nearly all. participants randomized? | | | Y |  |
|  | 3.2 If N/PN/NI to 3.1: Is there evidence that result was not biased by missing outcome data? | | | NA |  |
|  | 3.3 If N/PN to 3.2: Could missingness in the outcome depend on its true value? | | | NA |  |
|  | 3.4 If Y/PY/NI to 3.3: Is it likely that missingness in the outcome depended on its true value? | | | NA |  |
|  | **Risk of bias judgement** | | | **Low** |  |
| **Bias in measurement of the outcome** | 4.1 Was the method of measuring the outcome inappropriate? | | | N |  |
|  | 4.2 Could measurement or ascertainment of the outcome have differed between intervention groups? | | | PN |  |
|  | 4.3 Were outcome assessors aware of the intervention received by study participants? | | | PY |  |
|  | 4.4 If Y/PY/NI to 4.3: Could assessment of the outcome have been influenced by knowledge of intervention received? | | | NI |  |
|  | 4.5 If Y/PY/NI to 4.4: Is it likely that assessment of the outcome was influenced by knowledge of intervention received? | | | PN |  |
|  | **Risk of bias judgement** | | | **Some concerns** |  |
| **Bias in selection of the reported result** | 5.1 Were the data that produced this result analysed in accordance with a pre-specified analysis plan that was finalized before unblinded outcome data were available for analysis? | | | Y |  |
|  | 5.2 ... multiple eligible outcome measurements (e.g. scales. definitions. time points) within the outcome domain? | | | N |  |
|  | 5.3 ... multiple eligible analyses of the data? | | | N |  |
|  | **Risk of bias judgement** | | | **Low** |  |
| **Overall bias** | **Risk of bias judgement** | | | **Some concerns** |  |
|  |  |  |  |  |  |
| **Yilmaz 2022^60^** |  |  |  |  |  |
| **Domain** | **Signalling question** | | | **Response** | **Comments** |
| **Bias arising from the randomization process** | 1.1 Was the allocation sequence random? | | | PY | No clear mention to the randomization process nor sequence generation. but mention to the envelop opened by the surgeon. Not clearly specified if prior to surgery. |
|  | 1.2 Was the allocation sequence concealed until participants were enrolled and assigned to interventions? | | | PY |  |
|  | 1.3 Did baseline differences between intervention groups suggest a problem with the randomization process? | | | PN |  |
|  | **Risk of bias judgement** | | | **Low** |  |
| **Bias due to deviations from intended interventions** | 2.1.Were participants aware of their assigned intervention during the trial? | | | PY |  |
|  | 2.2.Were carers and people delivering the interventions aware of participants' assigned intervention during the trial? | | | Y |  |
|  | 2.3. If Y/PY/NI to 2.1 or 2.2: Were there deviations from the intended intervention that arose because of the experimental context? | | | PN |  |
|  | 2.4 If Y/PY to 2.3: Were these deviations likely to have affected the outcome? | | | NA |  |
|  | 2.5. If Y/PY/NI to 2.4: Were these deviations from intended intervention balanced between groups? | | | NA |  |
|  | 2.6 Was an appropriate analysis used to estimate the effect of assignment to intervention? | | | NI | Absence of Intention to treat analysis |
|  | 2.7 If N/PN/NI to 2.6: Was there potential for a substantial impact (on the result) of the failure to analyse participants in the group to which they were randomized? | | | PY | The exckusion of participant who dropped out introduces bias and the exclusion of such participants can impact the effect size |
|  | **Risk of bias judgement** | | | **High** |  |
| **Bias due to missing outcome data** | 3.1 Were data for this outcome available for all. or nearly all. participants randomized? | | | N |  |
|  | 3.2 If N/PN/NI to 3.1: Is there evidence that result was not biased by missing outcome data? | | | PN |  |
|  | 3.3 If N/PN to 3.2: Could missingness in the outcome depend on its true value? | | | NI |  |
|  | 3.4 If Y/PY/NI to 3.3: Is it likely that missingness in the outcome depended on its true value? | | | N |  |
|  | **Risk of bias judgement** | | | **Some concerns** |  |
| **Bias in measurement of the outcome** | 4.1 Was the method of measuring the outcome inappropriate? | | | PN |  |
|  | 4.2 Could measurement or ascertainment of the outcome have differed between intervention groups? | | | PN |  |
|  | 4.3 Were outcome assessors aware of the intervention received by study participants? | | | PY |  |
|  | 4.4 If Y/PY/NI to 4.3: Could assessment of the outcome have been influenced by knowledge of intervention received? | | | NI |  |
|  | 4.5 If Y/PY/NI to 4.4: Is it likely that assessment of the outcome was influenced by knowledge of intervention received? | | | PN |  |
|  | **Risk of bias judgement** | | | **Some concerns** |  |
| **Bias in selection of the reported result** | 5.1 Were the data that produced this result analysed in accordance with a pre-specified analysis plan that was finalized before unblinded outcome data were available for analysis? | | | Y |  |
|  | 5.2 ... multiple eligible outcome measurements (e.g. scales. definitions. time points) within the outcome domain? | | | N |  |
|  | 5.3 ... multiple eligible analyses of the data? | | | N |  |
|  | **Risk of bias judgement** | | | **Low** |  |
| **Overall bias** | **Risk of bias judgement** | | | **High** |  |
|  |  |  |  |  |  |
| **Bakhishov 2020^35^** |  |  |  |  |  |
| **Domain** | **Signalling question** | | | **Response** | **Comments** |
| **Bias arising from the randomization process** | 1.1 Was the allocation sequence random? | | | Y | Permuted block design used. Envelopes opened immediately after preparation of the recipient sites |
|  | 1.2 Was the allocation sequence concealed until participants were enrolled and assigned to interventions? | | | Y |  |
|  | 1.3 Did baseline differences between intervention groups suggest a problem with the randomization process? | | | N |  |
|  | **Risk of bias judgement** | | | **Low** |  |
| **Bias due to deviations from intended interventions** | 2.1.Were participants aware of their assigned intervention during the trial? | | | Y | Single blinded |
|  | 2.2.Were carers and people delivering the interventions aware of participants' assigned intervention during the trial? | | | Y |  |
|  | 2.3. If Y/PY/NI to 2.1 or 2.2: Were there deviations from the intended intervention that arose because of the experimental context? | | | N |  |
|  | 2.4 If Y/PY to 2.3: Were these deviations likely to have affected the outcome? | | | NA |  |
|  | 2.5. If Y/PY/NI to 2.4: Were these deviations from intended intervention balanced between groups? | | | NA |  |
|  | 2.6 Was an appropriate analysis used to estimate the effect of assignment to intervention? | | | NI | No ITT and exclusion from the analysis of 7 dropouts |
|  | 2.7 If N/PN/NI to 2.6: Was there potential for a substantial impact (on the result) of the failure to analyse participants in the group to which they were randomized? | | | PY | 7 patients are 20% of the sample |
|  | **Risk of bias judgement** | | | **High** |  |
| **Bias due to missing outcome data** | 3.1 Were data for this outcome available for all. or nearly all. participants randomized? | | | N | Data are not available for all participants randomized |
|  | 3.2 If N/PN/NI to 3.1: Is there evidence that result was not biased by missing outcome data? | | | N |  |
|  | 3.3 If N/PN to 3.2: Could missingness in the outcome depend on its true value? | | | NI |  |
|  | 3.4 If Y/PY/NI to 3.3: Is it likely that missingness in the outcome depended on its true value? | | | NI |  |
|  | **Risk of bias judgement** | | | **High** |  |
| **Bias in measurement of the outcome** | 4.1 Was the method of measuring the outcome inappropriate? | | | N |  |
|  | 4.2 Could measurement or ascertainment of the outcome have differed between intervention groups? | | | N |  |
|  | 4.3 Were outcome assessors aware of the intervention received by study participants? | | | Y |  |
|  | 4.4 If Y/PY/NI to 4.3: Could assessment of the outcome have been influenced by knowledge of intervention received? | | | NI |  |
|  | 4.5 If Y/PY/NI to 4.4: Is it likely that assessment of the outcome was influenced by knowledge of intervention received? | | | PN |  |
|  | **Risk of bias judgement** | | | **Some concerns** |  |
| **Bias in selection of the reported result** | 5.1 Were the data that produced this result analysed in accordance with a pre-specified analysis plan that was finalized before unblinded outcome data were available for analysis? | | | Y |  |
|  | 5.2 ... multiple eligible outcome measurements (e.g. scales. definitions. time points) within the outcome domain? | | | N |  |
|  | 5.3 ... multiple eligible analyses of the data? | | | N |  |
|  | **Risk of bias judgement** | | | **Low** |  |
| **Overall bias** | **Risk of bias judgement** | | | **High** |  |
|  |  |  |  |  |  |
| **Meza-Mauricio 2021^36^** |  |  |  |  |  |
| **Domain** | **Signalling question** | | | **Response** | **Comments** |
| **Bias arising from the randomization process** | 1.1 Was the allocation sequence random? | | | Y | Randomization and allocation concealment was guaranteed by an operator not involved in the clinical trial. Envelopes were opened during surgery after flap elevation |
|  | 1.2 Was the allocation sequence concealed until participants were enrolled and assigned to interventions? | | | Y |  |
|  | 1.3 Did baseline differences between intervention groups suggest a problem with the randomization process? | | | N |  |
|  | **Risk of bias judgement** | | | **Low** |  |
| **Bias due to deviations from intended interventions** | 2.1.Were participants aware of their assigned intervention during the trial? | | | PY |  |
|  | 2.2.Were carers and people delivering the interventions aware of participants' assigned intervention during the trial? | | | Y |  |
|  | 2.3. If Y/PY/NI to 2.1 or 2.2: Were there deviations from the intended intervention that arose because of the experimental context? | | | PN |  |
|  | 2.4 If Y/PY to 2.3: Were these deviations likely to have affected the outcome? | | | NA |  |
|  | 2.5. If Y/PY/NI to 2.4: Were these deviations from intended intervention balanced between groups? | | | NA |  |
|  | 2.6 Was an appropriate analysis used to estimate the effect of assignment to intervention? | | | NI | No mention to ITT |
|  | 2.7 If N/PN/NI to 2.6: Was there potential for a substantial impact (on the result) of the failure to analyse participants in the group to which they were randomized? | | | PN |  |
|  | **Risk of bias judgement** | | | **Some concerns** |  |
| **Bias due to missing outcome data** | 3.1 Were data for this outcome available for all. or nearly all. participants randomized? | | | N | 1 patient lost to follow up after receiving allocated intervention |
|  | 3.2 If N/PN/NI to 3.1: Is there evidence that result was not biased by missing outcome data? | | | PN |  |
|  | 3.3 If N/PN to 3.2: Could missingness in the outcome depend on its true value? | | | N |  |
|  | 3.4 If Y/PY/NI to 3.3: Is it likely that missingness in the outcome depended on its true value? | | | NA |  |
|  | **Risk of bias judgement** | | | **Low** |  |
| **Bias in measurement of the outcome** | 4.1 Was the method of measuring the outcome inappropriate? | | | N | RES evaluated by to masked and calibrated examiners. VAS |
|  | 4.2 Could measurement or ascertainment of the outcome have differed between intervention groups? | | | N |  |
|  | 4.3 Were outcome assessors aware of the intervention received by study participants? | | | PY |  |
|  | 4.4 If Y/PY/NI to 4.3: Could assessment of the outcome have been influenced by knowledge of intervention received? | | | NI |  |
|  | 4.5 If Y/PY/NI to 4.4: Is it likely that assessment of the outcome was influenced by knowledge of intervention received? | | | PN |  |
|  | **Risk of bias judgement** | | | **Some concerns** |  |
| **Bias in selection of the reported result** | 5.1 Were the data that produced this result analysed in accordance with a pre-specified analysis plan that was finalized before unblinded outcome data were available for analysis? | | | Y |  |
|  | 5.2 ... multiple eligible outcome measurements (e.g. scales. definitions. time points) within the outcome domain? | | | N |  |
|  | 5.3 ... multiple eligible analyses of the data? | | | N |  |
|  | **Risk of bias judgement** | | | **Low** |  |
| **Overall bias** | **Risk of bias judgement** | | | **Some concerns** |  |
|  |  |  |  |  |  |
| **Gorski 2022^37^** |  |  |  |  |  |
| **Domain** | **Signalling question** | | | **Response** | **Comments** |
| **Bias arising from the randomization process** | 1.1 Was the allocation sequence random? | | | Y | Computerized random number generator used. Allocation of treatment sites concelaed in opaque envelopes and releaved to the surgeon before the procedure |
|  | 1.2 Was the allocation sequence concealed until participants were enrolled and assigned to interventions? | | | Y |  |
|  | 1.3 Did baseline differences between intervention groups suggest a problem with the randomization process? | | | N |  |
|  | **Risk of bias judgement** | | | **Low** |  |
| **Bias due to deviations from intended interventions** | 2.1.Were participants aware of their assigned intervention during the trial? | | | N |  |
|  | 2.2.Were carers and people delivering the interventions aware of participants' assigned intervention during the trial? | | | Y |  |
|  | 2.3. If Y/PY/NI to 2.1 or 2.2: Were there deviations from the intended intervention that arose because of the experimental context? | | | PN |  |
|  | 2.4 If Y/PY to 2.3: Were these deviations likely to have affected the outcome? | | | NA |  |
|  | 2.5. If Y/PY/NI to 2.4: Were these deviations from intended intervention balanced between groups? | | | NA |  |
|  | 2.6 Was an appropriate analysis used to estimate the effect of assignment to intervention? | | | NI | No mention to ITT nor specified how |
|  | 2.7 If N/PN/NI to 2.6: Was there potential for a substantial impact (on the result) of the failure to analyse participants in the group to which they were randomized? | | | PN |  |
|  | **Risk of bias judgement** | | | **Some concerns** |  |
| **Bias due to missing outcome data** | 3.1 Were data for this outcome available for all. or nearly all. participants randomized? | | | N | 1 patient lost to follow up and was excluded from the analysis |
|  | 3.2 If N/PN/NI to 3.1: Is there evidence that result was not biased by missing outcome data? | | | PN | The missing data from the drop out combined with the lack of strategies to handle missing data (itt or imputation) could result in bias |
|  | 3.3 If N/PN to 3.2: Could missingness in the outcome depend on its true value? | | | PN |  |
|  | 3.4 If Y/PY/NI to 3.3: Is it likely that missingness in the outcome depended on its true value? | | | NA |  |
|  | **Risk of bias judgement** | | | **Low** |  |
| **Bias in measurement of the outcome** | 4.1 Was the method of measuring the outcome inappropriate? | | | N | VAS utilized for patients' esthetic satisfaction |
|  | 4.2 Could measurement or ascertainment of the outcome have differed between intervention groups? | | | N |  |
|  | 4.3 Were outcome assessors aware of the intervention received by study participants? | | | N |  |
|  | 4.4 If Y/PY/NI to 4.3: Could assessment of the outcome have been influenced by knowledge of intervention received? | | | NA |  |
|  | 4.5 If Y/PY/NI to 4.4: Is it likely that assessment of the outcome was influenced by knowledge of intervention received? | | | NA |  |
|  | **Risk of bias judgement** | | | **Low** |  |
| **Bias in selection of the reported result** | 5.1 Were the data that produced this result analysed in accordance with a pre-specified analysis plan that was finalized before unblinded outcome data were available for analysis? | | | Y |  |
|  | 5.2 ... multiple eligible outcome measurements (e.g. scales. definitions. time points) within the outcome domain? | | | N |  |
|  | 5.3 ... multiple eligible analyses of the data? | | | N |  |
|  | **Risk of bias judgement** | | | **Low** |  |
| **Overall bias** | **Risk of bias judgement** | | | **Some concerns** |  |
|  |  |  |  |  |  |
| **Cieślik-Wegemund 2016^38^** |  |  |  |  |  |
| **Domain** | **Signalling question** | | | **Response** | **Comments** |
| **Bias arising from the randomization process** | 1.1 Was the allocation sequence random? | | | Y | Block randomization method. sequence handed to the surgeon on the day the procedure was performed |
|  | 1.2 Was the allocation sequence concealed until participants were enrolled and assigned to interventions? | | | Y |  |
|  | 1.3 Did baseline differences between intervention groups suggest a problem with the randomization process? | | | N |  |
|  | **Risk of bias judgement** | | | **Low** |  |
| **Bias due to deviations from intended interventions** | 2.1.Were participants aware of their assigned intervention during the trial? | | | PY |  |
|  | 2.2.Were carers and people delivering the interventions aware of participants' assigned intervention during the trial? | | | Y |  |
|  | 2.3. If Y/PY/NI to 2.1 or 2.2: Were there deviations from the intended intervention that arose because of the experimental context? | | | PN |  |
|  | 2.4 If Y/PY to 2.3: Were these deviations likely to have affected the outcome? | | | NA |  |
|  | 2.5. If Y/PY/NI to 2.4: Were these deviations from intended intervention balanced between groups? | | | NA |  |
|  | 2.6 Was an appropriate analysis used to estimate the effect of assignment to intervention? | | | NI | No mention to ITT |
|  | 2.7 If N/PN/NI to 2.6: Was there potential for a substantial impact (on the result) of the failure to analyse participants in the group to which they were randomized? | | | PN |  |
|  | **Risk of bias judgement** | | | **Some concerns** |  |
| **Bias due to missing outcome data** | 3.1 Were data for this outcome available for all. or nearly all. participants randomized? | | | Y | All patients completed the study and attended all of the recall visits |
|  | 3.2 If N/PN/NI to 3.1: Is there evidence that result was not biased by missing outcome data? | | | NA |  |
|  | 3.3 If N/PN to 3.2: Could missingness in the outcome depend on its true value? | | | NA |  |
|  | 3.4 If Y/PY/NI to 3.3: Is it likely that missingness in the outcome depended on its true value? | | | NA |  |
|  | **Risk of bias judgement** | | | **Low** |  |
| **Bias in measurement of the outcome** | 4.1 Was the method of measuring the outcome inappropriate? | | | PN |  |
|  | 4.2 Could measurement or ascertainment of the outcome have differed between intervention groups? | | | N |  |
|  | 4.3 Were outcome assessors aware of the intervention received by study participants? | | | PY |  |
|  | 4.4 If Y/PY/NI to 4.3: Could assessment of the outcome have been influenced by knowledge of intervention received? | | | NI |  |
|  | 4.5 If Y/PY/NI to 4.4: Is it likely that assessment of the outcome was influenced by knowledge of intervention received? | | | PN |  |
|  | **Risk of bias judgement** | | | **Some concerns** |  |
| **Bias in selection of the reported result** | 5.1 Were the data that produced this result analysed in accordance with a pre-specified analysis plan that was finalized before unblinded outcome data were available for analysis? | | | Y |  |
|  | 5.2 ... multiple eligible outcome measurements (e.g. scales. definitions. time points) within the outcome domain? | | | N |  |
|  | 5.3 ... multiple eligible analyses of the data? | | | N |  |
|  | **Risk of bias judgement** | | | **Low** |  |
| **Overall bias** | **Risk of bias judgement** | | | **Some concerns** |  |
|  |  |  |  |  |  |
| **Tavelli 2022^39^** |  |  |  |  |  |
| **Domain** | **Signalling question** | | | **Response** | **Comments** |
| **Bias arising from the randomization process** | 1.1 Was the allocation sequence random? | | | Y | Stratified sequential randomization. sealed envelopes |
|  | 1.2 Was the allocation sequence concealed until participants were enrolled and assigned to interventions? | | | Y |  |
|  | 1.3 Did baseline differences between intervention groups suggest a problem with the randomization process? | | | N |  |
|  | **Risk of bias judgement** | | | **Low** |  |
| **Bias due to deviations from intended interventions** | 2.1.Were participants aware of their assigned intervention during the trial? | | | N | The surgeon received a sealed envelop with the patient ID number and a syringe containing a clear solution. either rhPDGF or saline |
|  | 2.2.Were carers and people delivering the interventions aware of participants' assigned intervention during the trial? | | | N |  |
|  | 2.3. If Y/PY/NI to 2.1 or 2.2: Were there deviations from the intended intervention that arose because of the experimental context? | | | NA |  |
|  | 2.4 If Y/PY to 2.3: Were these deviations likely to have affected the outcome? | | | NA |  |
|  | 2.5. If Y/PY/NI to 2.4: Were these deviations from intended intervention balanced between groups? | | | NA |  |
|  | 2.6 Was an appropriate analysis used to estimate the effect of assignment to intervention? | | | NI |  |
|  | 2.7 If N/PN/NI to 2.6: Was there potential for a substantial impact (on the result) of the failure to analyse participants in the group to which they were randomized? | | | PN |  |
|  | **Risk of bias judgement** | | | **Some concerns** |  |
| **Bias due to missing outcome data** | 3.1 Were data for this outcome available for all. or nearly all. participants randomized? | | | Y |  |
|  | 3.2 If N/PN/NI to 3.1: Is there evidence that result was not biased by missing outcome data? | | | NA |  |
|  | 3.3 If N/PN to 3.2: Could missingness in the outcome depend on its true value? | | | NA |  |
|  | 3.4 If Y/PY/NI to 3.3: Is it likely that missingness in the outcome depended on its true value? | | | NA |  |
|  | **Risk of bias judgement** | | | **Low** |  |
| **Bias in measurement of the outcome** | 4.1 Was the method of measuring the outcome inappropriate? | | | N |  |
|  | 4.2 Could measurement or ascertainment of the outcome have differed between intervention groups? | | | N |  |
|  | 4.3 Were outcome assessors aware of the intervention received by study participants? | | | N |  |
|  | 4.4 If Y/PY/NI to 4.3: Could assessment of the outcome have been influenced by knowledge of intervention received? | | | NA |  |
|  | 4.5 If Y/PY/NI to 4.4: Is it likely that assessment of the outcome was influenced by knowledge of intervention received? | | | NA |  |
|  | **Risk of bias judgement** | | | **Low** |  |
| **Bias in selection of the reported result** | 5.1 Were the data that produced this result analysed in accordance with a pre-specified analysis plan that was finalized before unblinded outcome data were available for analysis? | | | Y |  |
|  | 5.2 ... multiple eligible outcome measurements (e.g. scales. definitions. time points) within the outcome domain? | | | N |  |
|  | 5.3 ... multiple eligible analyses of the data? | | | N |  |
|  | **Risk of bias judgement** | | | **Low** |  |
| **Overall bias** | **Risk of bias judgement** | | | **Some concerns** |  |
|  |  |  |  |  |  |
| **Aroca 2013^61^** |  |  |  |  |  |
| **Domain** | **Signalling question** | | | **Response** | **Comments** |
| **Bias arising from the randomization process** | 1.1 Was the allocation sequence random? | | | PY | Randomization code broken when tunnel preparation was completed |
|  | 1.2 Was the allocation sequence concealed until participants were enrolled and assigned to interventions? | | | Y |  |
|  | 1.3 Did baseline differences between intervention groups suggest a problem with the randomization process? | | | PN |  |
|  | **Risk of bias judgement** | | | **Low** |  |
| **Bias due to deviations from intended interventions** | 2.1.Were participants aware of their assigned intervention during the trial? | | | PY |  |
|  | 2.2.Were carers and people delivering the interventions aware of participants' assigned intervention during the trial? | | | Y |  |
|  | 2.3. If Y/PY/NI to 2.1 or 2.2: Were there deviations from the intended intervention that arose because of the experimental context? | | | PN |  |
|  | 2.4 If Y/PY to 2.3: Were these deviations likely to have affected the outcome? | | | NA |  |
|  | 2.5. If Y/PY/NI to 2.4: Were these deviations from intended intervention balanced between groups? | | | NA |  |
|  | 2.6 Was an appropriate analysis used to estimate the effect of assignment to intervention? | | | NI | No ITT mentioned |
|  | 2.7 If N/PN/NI to 2.6: Was there potential for a substantial impact (on the result) of the failure to analyse participants in the group to which they were randomized? | | | PN |  |
|  | **Risk of bias judgement** | | | **Some concerns** |  |
| **Bias due to missing outcome data** | 3.1 Were data for this outcome available for all. or nearly all. participants randomized? | | | Y |  |
|  | 3.2 If N/PN/NI to 3.1: Is there evidence that result was not biased by missing outcome data? | | | NA |  |
|  | 3.3 If N/PN to 3.2: Could missingness in the outcome depend on its true value? | | | NA |  |
|  | 3.4 If Y/PY/NI to 3.3: Is it likely that missingness in the outcome depended on its true value? | | | NA |  |
|  | **Risk of bias judgement** | | | **Low** |  |
| **Bias in measurement of the outcome** | 4.1 Was the method of measuring the outcome inappropriate? | | | PN |  |
|  | 4.2 Could measurement or ascertainment of the outcome have differed between intervention groups? | | | PN |  |
|  | 4.3 Were outcome assessors aware of the intervention received by study participants? | | | PY |  |
|  | 4.4 If Y/PY/NI to 4.3: Could assessment of the outcome have been influenced by knowledge of intervention received? | | | NI |  |
|  | 4.5 If Y/PY/NI to 4.4: Is it likely that assessment of the outcome was influenced by knowledge of intervention received? | | | PN |  |
|  | **Risk of bias judgement** | | | **Some concerns** |  |
| **Bias in selection of the reported result** | 5.1 Were the data that produced this result analysed in accordance with a pre-specified analysis plan that was finalized before unblinded outcome data were available for analysis? | | | Y |  |
|  | 5.2 ... multiple eligible outcome measurements (e.g. scales. definitions. time points) within the outcome domain? | | | N |  |
|  | 5.3 ... multiple eligible analyses of the data? | | | N |  |
|  | **Risk of bias judgement** | | | **Low** |  |
| **Overall bias** | **Risk of bias judgement** | | | **Some concerns** |  |
|  |  |  |  |  |  |
| **Zucchelli 2014^40^** |  |  |  |  |  |
| **Domain** | **Signalling question** | | | **Response** | **Comments** |
| **Bias arising from the randomization process** | 1.1 Was the allocation sequence random? | | | Y | Computer generated sequence. envelopes opened before surgery |
|  | 1.2 Was the allocation sequence concealed until participants were enrolled and assigned to interventions? | | | Y |  |
|  | 1.3 Did baseline differences between intervention groups suggest a problem with the randomization process? | | | N |  |
|  | **Risk of bias judgement** | | | **Low** |  |
| **Bias due to deviations from intended interventions** | 2.1.Were participants aware of their assigned intervention during the trial? | | | PY |  |
|  | 2.2.Were carers and people delivering the interventions aware of participants' assigned intervention during the trial? | | | Y |  |
|  | 2.3. If Y/PY/NI to 2.1 or 2.2: Were there deviations from the intended intervention that arose because of the experimental context? | | | PN |  |
|  | 2.4 If Y/PY to 2.3: Were these deviations likely to have affected the outcome? | | | NA |  |
|  | 2.5. If Y/PY/NI to 2.4: Were these deviations from intended intervention balanced between groups? | | | NA |  |
|  | 2.6 Was an appropriate analysis used to estimate the effect of assignment to intervention? | | | NI | No ITT |
|  | 2.7 If N/PN/NI to 2.6: Was there potential for a substantial impact (on the result) of the failure to analyse participants in the group to which they were randomized? | | | PN |  |
|  | **Risk of bias judgement** | | | **Some concerns** |  |
| **Bias due to missing outcome data** | 3.1 Were data for this outcome available for all. or nearly all. participants randomized? | | | Y | No patients were lost to follow up |
|  | 3.2 If N/PN/NI to 3.1: Is there evidence that result was not biased by missing outcome data? | | | NA |  |
|  | 3.3 If N/PN to 3.2: Could missingness in the outcome depend on its true value? | | | NA |  |
|  | 3.4 If Y/PY/NI to 3.3: Is it likely that missingness in the outcome depended on its true value? | | | NA |  |
|  | **Risk of bias judgement** | | | **Low** |  |
| **Bias in measurement of the outcome** | 4.1 Was the method of measuring the outcome inappropriate? | | | N |  |
|  | 4.2 Could measurement or ascertainment of the outcome have differed between intervention groups? | | | PN |  |
|  | 4.3 Were outcome assessors aware of the intervention received by study participants? | | | PY |  |
|  | 4.4 If Y/PY/NI to 4.3: Could assessment of the outcome have been influenced by knowledge of intervention received? | | | NI |  |
|  | 4.5 If Y/PY/NI to 4.4: Is it likely that assessment of the outcome was influenced by knowledge of intervention received? | | | PN |  |
|  | **Risk of bias judgement** | | | **Some concerns** |  |
| **Bias in selection of the reported result** | 5.1 Were the data that produced this result analysed in accordance with a pre-specified analysis plan that was finalized before unblinded outcome data were available for analysis? | | | Y |  |
|  | 5.2 ... multiple eligible outcome measurements (e.g. scales. definitions. time points) within the outcome domain? | | | N |  |
|  | 5.3 ... multiple eligible analyses of the data? | | | N |  |
|  | **Risk of bias judgement** | | | **Low** |  |
| **Overall bias** | **Risk of bias judgement** | | | **Some concerns** |  |
|  |  |  |  |  |  |
| **Fernandez-Jimenez 2023^62^** |  |  |  |  |  |
| **Domain** | **Signalling question** | | | **Response** | **Comments** |
| **Bias arising from the randomization process** | 1.1 Was the allocation sequence random? | | | Y | Block randomization. assignments hidden until intervention |
|  | 1.2 Was the allocation sequence concealed until participants were enrolled and assigned to interventions? | | | Y |  |
|  | 1.3 Did baseline differences between intervention groups suggest a problem with the randomization process? | | | N |  |
|  | **Risk of bias judgement** | | | **Low** |  |
| **Bias due to deviations from intended interventions** | 2.1.Were participants aware of their assigned intervention during the trial? | | | N |  |
|  | 2.2.Were carers and people delivering the interventions aware of participants' assigned intervention during the trial? | | | Y |  |
|  | 2.3. If Y/PY/NI to 2.1 or 2.2: Were there deviations from the intended intervention that arose because of the experimental context? | | | N |  |
|  | 2.4 If Y/PY to 2.3: Were these deviations likely to have affected the outcome? | | | NA |  |
|  | 2.5. If Y/PY/NI to 2.4: Were these deviations from intended intervention balanced between groups? | | | NA |  |
|  | 2.6 Was an appropriate analysis used to estimate the effect of assignment to intervention? | | | NI |  |
|  | 2.7 If N/PN/NI to 2.6: Was there potential for a substantial impact (on the result) of the failure to analyse participants in the group to which they were randomized? | | | PN |  |
|  | **Risk of bias judgement** | | | **Some concerns** |  |
| **Bias due to missing outcome data** | 3.1 Were data for this outcome available for all. or nearly all. participants randomized? | | | N |  |
|  | 3.2 If N/PN/NI to 3.1: Is there evidence that result was not biased by missing outcome data? | | | PN |  |
|  | 3.3 If N/PN to 3.2: Could missingness in the outcome depend on its true value? | | | N | Reason for drop out specified. so no risk of Missing not at random |
|  | 3.4 If Y/PY/NI to 3.3: Is it likely that missingness in the outcome depended on its true value? | | | NA |  |
|  | **Risk of bias judgement** | | | **Low** |  |
| **Bias in measurement of the outcome** | 4.1 Was the method of measuring the outcome inappropriate? | | | N |  |
|  | 4.2 Could measurement or ascertainment of the outcome have differed between intervention groups? | | | PN |  |
|  | 4.3 Were outcome assessors aware of the intervention received by study participants? | | | N | Triple blind |
|  | 4.4 If Y/PY/NI to 4.3: Could assessment of the outcome have been influenced by knowledge of intervention received? | | | NA |  |
|  | 4.5 If Y/PY/NI to 4.4: Is it likely that assessment of the outcome was influenced by knowledge of intervention received? | | | NA |  |
|  | **Risk of bias judgement** | | | **Low** |  |
| **Bias in selection of the reported result** | 5.1 Were the data that produced this result analysed in accordance with a pre-specified analysis plan that was finalized before unblinded outcome data were available for analysis? | | | Y |  |
|  | 5.2 ... multiple eligible outcome measurements (e.g. scales. definitions. time points) within the outcome domain? | | | N |  |
|  | 5.3 ... multiple eligible analyses of the data? | | | N |  |
|  | **Risk of bias judgement** | | | **Low** |  |
| **Overall bias** | **Risk of bias judgement** | | | **Some concerns** |  |
|  |  |  |  |  |  |
| Gorski 2023^41^ |  |  |  |  |  |
| **Domain** | **Signalling question** | | | **Response** | **Comments** |
| **Bias arising from the randomization process** | 1.1 Was the allocation sequence random? | | | Y |  |
|  | 1.2 Was the allocation sequence concealed until participants were enrolled and assigned to interventions? | | | Y |  |
|  | 1.3 Did baseline differences between intervention groups suggest a problem with the randomization process? | | | N |  |
|  | **Risk of bias judgement** | | | **Low** |  |
| **Bias due to deviations from intended interventions** | 2.1.Were participants aware of their assigned intervention during the trial? | | | NI |  |
|  | 2.2.Were carers and people delivering the interventions aware of participants' assigned intervention during the trial? | | | Y |  |
|  | 2.3. If Y/PY/NI to 2.1 or 2.2: Were there deviations from the intended intervention that arose because of the experimental context? | | | PN |  |
|  | 2.4 If Y/PY to 2.3: Were these deviations likely to have affected the outcome? | | | NA |  |
|  | 2.5. If Y/PY/NI to 2.4: Were these deviations from intended intervention balanced between groups? | | | NA |  |
|  | 2.6 Was an appropriate analysis used to estimate the effect of assignment to intervention? | | | NI |  |
|  | 2.7 If N/PN/NI to 2.6: Was there potential for a substantial impact (on the result) of the failure to analyse participants in the group to which they were randomized? | | | PN |  |
|  | **Risk of bias judgement** | | | **Some concerns** |  |
| **Bias due to missing outcome data** | 3.1 Were data for this outcome available for all. or nearly all. participants randomized? | | | Y |  |
|  | 3.2 If N/PN/NI to 3.1: Is there evidence that result was not biased by missing outcome data? | | | NA |  |
|  | 3.3 If N/PN to 3.2: Could missingness in the outcome depend on its true value? | | | NA |  |
|  | 3.4 If Y/PY/NI to 3.3: Is it likely that missingness in the outcome depended on its true value? | | | NA |  |
|  | **Risk of bias judgement** | | | **Low** |  |
| **Bias in measurement of the outcome** | 4.1 Was the method of measuring the outcome inappropriate? | | | N |  |
|  | 4.2 Could measurement or ascertainment of the outcome have differed between intervention groups? | | | PN |  |
|  | 4.3 Were outcome assessors aware of the intervention received by study participants? | | | NI |  |
|  | 4.4 If Y/PY/NI to 4.3: Could assessment of the outcome have been influenced by knowledge of intervention received? | | | PN |  |
|  | 4.5 If Y/PY/NI to 4.4: Is it likely that assessment of the outcome was influenced by knowledge of intervention received? | | | NA |  |
|  | **Risk of bias judgement** | | | **Low** |  |
| **Bias in selection of the reported result** | 5.1 Were the data that produced this result analysed in accordance with a pre-specified analysis plan that was finalized before unblinded outcome data were available for analysis? | | | Y |  |
|  | 5.2 ... multiple eligible outcome measurements (e.g. scales. definitions. time points) within the outcome domain? | | | N |  |
|  | 5.3 ... multiple eligible analyses of the data? | | | N |  |
|  | **Risk of bias judgement** | | | **Low** |  |
| **Overall bias** | **Risk of bias judgement** | | | **Some concerns** |  |
|  |  |  |  |  |  |
| **Gorski 2020^42^** |  |  |  |  |  |
| **Domain** | **Signalling question** | | | **Response** | **Comments** |
| **Bias arising from the randomization process** | 1.1 Was the allocation sequence random? | | | Y | Random sequence. envelopes opened immediately before the procedure |
|  | 1.2 Was the allocation sequence concealed until participants were enrolled and assigned to interventions? | | | Y |  |
|  | 1.3 Did baseline differences between intervention groups suggest a problem with the randomization process? | | | N |  |
|  | **Risk of bias judgement** | | | **Low** |  |
| **Bias due to deviations from intended interventions** | 2.1.Were participants aware of their assigned intervention during the trial? | | | NI |  |
|  | 2.2.Were carers and people delivering the interventions aware of participants' assigned intervention during the trial? | | | Y |  |
|  | 2.3. If Y/PY/NI to 2.1 or 2.2: Were there deviations from the intended intervention that arose because of the experimental context? | | | N |  |
|  | 2.4 If Y/PY to 2.3: Were these deviations likely to have affected the outcome? | | | NA |  |
|  | 2.5. If Y/PY/NI to 2.4: Were these deviations from intended intervention balanced between groups? | | | NA |  |
|  | 2.6 Was an appropriate analysis used to estimate the effect of assignment to intervention? | | | NI |  |
|  | 2.7 If N/PN/NI to 2.6: Was there potential for a substantial impact (on the result) of the failure to analyse participants in the group to which they were randomized? | | | PN |  |
|  | **Risk of bias judgement** | | | **Some concerns** |  |
| **Bias due to missing outcome data** | 3.1 Were data for this outcome available for all. or nearly all. participants randomized? | | | Y |  |
|  | 3.2 If N/PN/NI to 3.1: Is there evidence that result was not biased by missing outcome data? | | | NA |  |
|  | 3.3 If N/PN to 3.2: Could missingness in the outcome depend on its true value? | | | NA |  |
|  | 3.4 If Y/PY/NI to 3.3: Is it likely that missingness in the outcome depended on its true value? | | | NA |  |
|  | **Risk of bias judgement** | | | **Low** |  |
| **Bias in measurement of the outcome** | 4.1 Was the method of measuring the outcome inappropriate? | | | N |  |
|  | 4.2 Could measurement or ascertainment of the outcome have differed between intervention groups? | | | PN |  |
|  | 4.3 Were outcome assessors aware of the intervention received by study participants? | | | NI |  |
|  | 4.4 If Y/PY/NI to 4.3: Could assessment of the outcome have been influenced by knowledge of intervention received? | | | PN |  |
|  | 4.5 If Y/PY/NI to 4.4: Is it likely that assessment of the outcome was influenced by knowledge of intervention received? | | | NA |  |
|  | **Risk of bias judgement** | | | **Low** |  |
| **Bias in selection of the reported result** | 5.1 Were the data that produced this result analysed in accordance with a pre-specified analysis plan that was finalized before unblinded outcome data were available for analysis? | | | Y |  |
|  | 5.2 ... multiple eligible outcome measurements (e.g. scales. definitions. time points) within the outcome domain? | | | N |  |
|  | 5.3 ... multiple eligible analyses of the data? | | | N |  |
|  | **Risk of bias judgement** | | | **Low** |  |
| **Overall bias** | **Risk of bias judgement** | | | **Some concerns** |  |
|  |  |  |  |  |  |
| **Zucchelli 2009^43^** |  |  |  |  |  |
| **Domain** | **Signalling question** | | | **Response** | **Comments** |
| **Bias arising from the randomization process** | 1.1 Was the allocation sequence random? | | | Y |  |
|  | 1.2 Was the allocation sequence concealed until participants were enrolled and assigned to interventions? | | | Y |  |
|  | 1.3 Did baseline differences between intervention groups suggest a problem with the randomization process? | | | N |  |
|  | **Risk of bias judgement** | | | **Low** |  |
| **Bias due to deviations from intended interventions** | 2.1.Were participants aware of their assigned intervention during the trial? | | | NI |  |
|  | 2.2.Were carers and people delivering the interventions aware of participants' assigned intervention during the trial? | | | Y |  |
|  | 2.3. If Y/PY/NI to 2.1 or 2.2: Were there deviations from the intended intervention that arose because of the experimental context? | | | PN |  |
|  | 2.4 If Y/PY to 2.3: Were these deviations likely to have affected the outcome? | | | NA |  |
|  | 2.5. If Y/PY/NI to 2.4: Were these deviations from intended intervention balanced between groups? | | | NA |  |
|  | 2.6 Was an appropriate analysis used to estimate the effect of assignment to intervention? | | | NI | No ITT |
|  | 2.7 If N/PN/NI to 2.6: Was there potential for a substantial impact (on the result) of the failure to analyse participants in the group to which they were randomized? | | | PN |  |
|  | **Risk of bias judgement** | | | **Some concerns** |  |
| **Bias due to missing outcome data** | 3.1 Were data for this outcome available for all. or nearly all. participants randomized? | | | Y |  |
|  | 3.2 If N/PN/NI to 3.1: Is there evidence that result was not biased by missing outcome data? | | | NA |  |
|  | 3.3 If N/PN to 3.2: Could missingness in the outcome depend on its true value? | | | NA |  |
|  | 3.4 If Y/PY/NI to 3.3: Is it likely that missingness in the outcome depended on its true value? | | | NA |  |
|  | **Risk of bias judgement** | | | **Low** |  |
| **Bias in measurement of the outcome** | 4.1 Was the method of measuring the outcome inappropriate? | | | N |  |
|  | 4.2 Could measurement or ascertainment of the outcome have differed between intervention groups? | | | PN |  |
|  | 4.3 Were outcome assessors aware of the intervention received by study participants? | | | NI |  |
|  | 4.4 If Y/PY/NI to 4.3: Could assessment of the outcome have been influenced by knowledge of intervention received? | | | PN |  |
|  | 4.5 If Y/PY/NI to 4.4: Is it likely that assessment of the outcome was influenced by knowledge of intervention received? | | | NA |  |
|  | **Risk of bias judgement** | | | **Low** |  |
| **Bias in selection of the reported result** | 5.1 Were the data that produced this result analysed in accordance with a pre-specified analysis plan that was finalized before unblinded outcome data were available for analysis? | | | Y |  |
|  | 5.2 ... multiple eligible outcome measurements (e.g. scales. definitions. time points) within the outcome domain? | | | N |  |
|  | 5.3 ... multiple eligible analyses of the data? | | | N |  |
|  | **Risk of bias judgement** | | | **Low** |  |
| **Overall bias** | **Risk of bias judgement** | | | **Some concerns** |  |
|  |  |  |  |  |  |
| **Nahas 2020^44^** |  |  |  |  |  |
| **Domain** | **Signalling question** | | | **Response** | **Comments** |
| **Bias arising from the randomization process** | 1.1 Was the allocation sequence random? | | | PY |  |
|  | 1.2 Was the allocation sequence concealed until participants were enrolled and assigned to interventions? | | | Y |  |
|  | 1.3 Did baseline differences between intervention groups suggest a problem with the randomization process? | | | N |  |
|  | **Risk of bias judgement** | | | **Low** |  |
| **Bias due to deviations from intended interventions** | 2.1.Were participants aware of their assigned intervention during the trial? | | | Y |  |
|  | 2.2.Were carers and people delivering the interventions aware of participants' assigned intervention during the trial? | | | Y |  |
|  | 2.3. If Y/PY/NI to 2.1 or 2.2: Were there deviations from the intended intervention that arose because of the experimental context? | | | PN |  |
|  | 2.4 If Y/PY to 2.3: Were these deviations likely to have affected the outcome? | | | NA |  |
|  | 2.5. If Y/PY/NI to 2.4: Were these deviations from intended intervention balanced between groups? | | | NA |  |
|  | 2.6 Was an appropriate analysis used to estimate the effect of assignment to intervention? | | | NI |  |
|  | 2.7 If N/PN/NI to 2.6: Was there potential for a substantial impact (on the result) of the failure to analyse participants in the group to which they were randomized? | | | PN |  |
|  | **Risk of bias judgement** | | | **Some concerns** |  |
| **Bias due to missing outcome data** | 3.1 Were data for this outcome available for all. or nearly all. participants randomized? | | | Y |  |
|  | 3.2 If N/PN/NI to 3.1: Is there evidence that result was not biased by missing outcome data? | | | NA |  |
|  | 3.3 If N/PN to 3.2: Could missingness in the outcome depend on its true value? | | | NA |  |
|  | 3.4 If Y/PY/NI to 3.3: Is it likely that missingness in the outcome depended on its true value? | | | NA |  |
|  | **Risk of bias judgement** | | | **Low** |  |
| **Bias in measurement of the outcome** | 4.1 Was the method of measuring the outcome inappropriate? | | | N |  |
|  | 4.2 Could measurement or ascertainment of the outcome have differed between intervention groups? | | | PN |  |
|  | 4.3 Were outcome assessors aware of the intervention received by study participants? | | | Y |  |
|  | 4.4 If Y/PY/NI to 4.3: Could assessment of the outcome have been influenced by knowledge of intervention received? | | | NI |  |
|  | 4.5 If Y/PY/NI to 4.4: Is it likely that assessment of the outcome was influenced by knowledge of intervention received? | | | PN |  |
|  | **Risk of bias judgement** | | | **Some concerns** |  |
| **Bias in selection of the reported result** | 5.1 Were the data that produced this result analysed in accordance with a pre-specified analysis plan that was finalized before unblinded outcome data were available for analysis? | | | Y |  |
|  | 5.2 ... multiple eligible outcome measurements (e.g. scales. definitions. time points) within the outcome domain? | | | N |  |
|  | 5.3 ... multiple eligible analyses of the data? | | | N |  |
|  | **Risk of bias judgement** | | | **Low** |  |
| **Overall bias** | **Risk of bias judgement** | | | **Some concerns** |  |
|  |  |  |  |  |  |
| **Kuka 2018^45^** |  |  |  |  |  |
| **Domain** | **Signalling question** | | | **Response** | **Comments** |
| **Bias arising from the randomization process** | 1.1 Was the allocation sequence random? | | | Y | Coin toss before surgery |
|  | 1.2 Was the allocation sequence concealed until participants were enrolled and assigned to interventions? | | | Y |  |
|  | 1.3 Did baseline differences between intervention groups suggest a problem with the randomization process? | | | N |  |
|  | **Risk of bias judgement** | | | **Low** |  |
| **Bias due to deviations from intended interventions** | 2.1.Were participants aware of their assigned intervention during the trial? | | | Y |  |
|  | 2.2.Were carers and people delivering the interventions aware of participants' assigned intervention during the trial? | | | Y |  |
|  | 2.3. If Y/PY/NI to 2.1 or 2.2: Were there deviations from the intended intervention that arose because of the experimental context? | | | PN |  |
|  | 2.4 If Y/PY to 2.3: Were these deviations likely to have affected the outcome? | | | NA |  |
|  | 2.5. If Y/PY/NI to 2.4: Were these deviations from intended intervention balanced between groups? | | | NA |  |
|  | 2.6 Was an appropriate analysis used to estimate the effect of assignment to intervention? | | | NI |  |
|  | 2.7 If N/PN/NI to 2.6: Was there potential for a substantial impact (on the result) of the failure to analyse participants in the group to which they were randomized? | | | N |  |
|  | **Risk of bias judgement** | | | **Some concerns** |  |
| **Bias due to missing outcome data** | 3.1 Were data for this outcome available for all. or nearly all. participants randomized? | | | Y |  |
|  | 3.2 If N/PN/NI to 3.1: Is there evidence that result was not biased by missing outcome data? | | | NA |  |
|  | 3.3 If N/PN to 3.2: Could missingness in the outcome depend on its true value? | | | NA |  |
|  | 3.4 If Y/PY/NI to 3.3: Is it likely that missingness in the outcome depended on its true value? | | | NA |  |
|  | **Risk of bias judgement** | | | **Low** |  |
| **Bias in measurement of the outcome** | 4.1 Was the method of measuring the outcome inappropriate? | | | N |  |
|  | 4.2 Could measurement or ascertainment of the outcome have differed between intervention groups? | | | PN |  |
|  | 4.3 Were outcome assessors aware of the intervention received by study participants? | | | Y |  |
|  | 4.4 If Y/PY/NI to 4.3: Could assessment of the outcome have been influenced by knowledge of intervention received? | | | NI |  |
|  | 4.5 If Y/PY/NI to 4.4: Is it likely that assessment of the outcome was influenced by knowledge of intervention received? | | | PN |  |
|  | **Risk of bias judgement** | | | **Some concerns** |  |
| **Bias in selection of the reported result** | 5.1 Were the data that produced this result analysed in accordance with a pre-specified analysis plan that was finalized before unblinded outcome data were available for analysis? | | | Y |  |
|  | 5.2 ... multiple eligible outcome measurements (e.g. scales. definitions. time points) within the outcome domain? | | | N |  |
|  | 5.3 ... multiple eligible analyses of the data? | | | N |  |
|  | **Risk of bias judgement** | | | **Low** |  |
| **Overall bias** | **Risk of bias judgement** | | | **Some concerns** |  |
|  |  |  |  |  |  |
| **Trivedi 2023^46^** |  |  |  |  |  |
| **Domain** | **Signalling question** | | | **Response** | **Comments** |
| **Bias arising from the randomization process** | 1.1 Was the allocation sequence random? | | | Y |  |
|  | 1.2 Was the allocation sequence concealed until participants were enrolled and assigned to interventions? | | | Y |  |
|  | 1.3 Did baseline differences between intervention groups suggest a problem with the randomization process? | | | N |  |
|  | **Risk of bias judgement** | | | **Low** |  |
| **Bias due to deviations from intended interventions** | 2.1.Were participants aware of their assigned intervention during the trial? | | | N |  |
|  | 2.2.Were carers and people delivering the interventions aware of participants' assigned intervention during the trial? | | | Y |  |
|  | 2.3. If Y/PY/NI to 2.1 or 2.2: Were there deviations from the intended intervention that arose because of the experimental context? | | | PN |  |
|  | 2.4 If Y/PY to 2.3: Were these deviations likely to have affected the outcome? | | | NA |  |
|  | 2.5. If Y/PY/NI to 2.4: Were these deviations from intended intervention balanced between groups? | | | NA |  |
|  | 2.6 Was an appropriate analysis used to estimate the effect of assignment to intervention? | | | NI |  |
|  | 2.7 If N/PN/NI to 2.6: Was there potential for a substantial impact (on the result) of the failure to analyse participants in the group to which they were randomized? | | | PN |  |
|  | **Risk of bias judgement** | | | **Some concerns** |  |
| **Bias due to missing outcome data** | 3.1 Were data for this outcome available for all. or nearly all. participants randomized? | | | Y |  |
|  | 3.2 If N/PN/NI to 3.1: Is there evidence that result was not biased by missing outcome data? | | | NA |  |
|  | 3.3 If N/PN to 3.2: Could missingness in the outcome depend on its true value? | | | NA |  |
|  | 3.4 If Y/PY/NI to 3.3: Is it likely that missingness in the outcome depended on its true value? | | | NA |  |
|  | **Risk of bias judgement** | | | **Low** |  |
| **Bias in measurement of the outcome** | 4.1 Was the method of measuring the outcome inappropriate? | | | N |  |
|  | 4.2 Could measurement or ascertainment of the outcome have differed between intervention groups? | | | PN |  |
|  | 4.3 Were outcome assessors aware of the intervention received by study participants? | | | N |  |
|  | 4.4 If Y/PY/NI to 4.3: Could assessment of the outcome have been influenced by knowledge of intervention received? | | | NA |  |
|  | 4.5 If Y/PY/NI to 4.4: Is it likely that assessment of the outcome was influenced by knowledge of intervention received? | | | NA |  |
|  | **Risk of bias judgement** | | | **Low** |  |
| **Bias in selection of the reported result** | 5.1 Were the data that produced this result analysed in accordance with a pre-specified analysis plan that was finalized before unblinded outcome data were available for analysis? | | | Y |  |
|  | 5.2 ... multiple eligible outcome measurements (e.g. scales. definitions. time points) within the outcome domain? | | | N |  |
|  | 5.3 ... multiple eligible analyses of the data? | | | N |  |
|  | **Risk of bias judgement** | | | **Low** |  |
| **Overall bias** | **Risk of bias judgement** | | | **Some concerns** |  |
|  |  |  |  |  |  |
| **Ahmedbeyli 2014^47^** |  |  |  |  |  |
| **Domain** | **Signalling question** | | | **Response** | **Comments** |
| **Bias arising from the randomization process** | 1.1 Was the allocation sequence random? | | | Y |  |
|  | 1.2 Was the allocation sequence concealed until participants were enrolled and assigned to interventions? | | | NI |  |
|  | 1.3 Did baseline differences between intervention groups suggest a problem with the randomization process? | | | N |  |
|  | **Risk of bias judgement** | | | **Some concerns** |  |
| **Bias due to deviations from intended interventions** | 2.1.Were participants aware of their assigned intervention during the trial? | | | NI |  |
|  | 2.2.Were carers and people delivering the interventions aware of participants' assigned intervention during the trial? | | | NI |  |
|  | 2.3. If Y/PY/NI to 2.1 or 2.2: Were there deviations from the intended intervention that arose because of the experimental context? | | | NI |  |
|  | 2.4 If Y/PY to 2.3: Were these deviations likely to have affected the outcome? | | | NA |  |
|  | 2.5. If Y/PY/NI to 2.4: Were these deviations from intended intervention balanced between groups? | | | NA |  |
|  | 2.6 Was an appropriate analysis used to estimate the effect of assignment to intervention? | | | NI |  |
|  | 2.7 If N/PN/NI to 2.6: Was there potential for a substantial impact (on the result) of the failure to analyse participants in the group to which they were randomized? | | | N |  |
|  | **Risk of bias judgement** | | | **Some concerns** |  |
| **Bias due to missing outcome data** | 3.1 Were data for this outcome available for all. or nearly all. participants randomized? | | | Y |  |
|  | 3.2 If N/PN/NI to 3.1: Is there evidence that result was not biased by missing outcome data? | | | NA |  |
|  | 3.3 If N/PN to 3.2: Could missingness in the outcome depend on its true value? | | | NA |  |
|  | 3.4 If Y/PY/NI to 3.3: Is it likely that missingness in the outcome depended on its true value? | | | NA |  |
|  | **Risk of bias judgement** | | | **Low** |  |
| **Bias in measurement of the outcome** | 4.1 Was the method of measuring the outcome inappropriate? | | | PN |  |
|  | 4.2 Could measurement or ascertainment of the outcome have differed between intervention groups? | | | NI |  |
|  | 4.3 Were outcome assessors aware of the intervention received by study participants? | | | NI |  |
|  | 4.4 If Y/PY/NI to 4.3: Could assessment of the outcome have been influenced by knowledge of intervention received? | | | NI |  |
|  | 4.5 If Y/PY/NI to 4.4: Is it likely that assessment of the outcome was influenced by knowledge of intervention received? | | | NI |  |
|  | **Risk of bias judgement** | | | **High** |  |
| **Bias in selection of the reported result** | 5.1 Were the data that produced this result analysed in accordance with a pre-specified analysis plan that was finalized before unblinded outcome data were available for analysis? | | | Y |  |
|  | 5.2 ... multiple eligible outcome measurements (e.g. scales. definitions. time points) within the outcome domain? | | | N |  |
|  | 5.3 ... multiple eligible analyses of the data? | | | N |  |
|  | **Risk of bias judgement** | | | **Low** |  |
| **Overall bias** | **Risk of bias judgement** | | | **High** |  |
|  |  |  |  |  |  |
| **Ozcelik 2011^48^** |  |  |  |  |  |
| **Domain** | **Signalling question** | | | **Response** | **Comments** |
| **Bias arising from the randomization process** | 1.1 Was the allocation sequence random? | | | Y | Coin toss. Envelopes opened at time of surgery |
|  | 1.2 Was the allocation sequence concealed until participants were enrolled and assigned to interventions? | | | Y |  |
|  | 1.3 Did baseline differences between intervention groups suggest a problem with the randomization process? | | | N |  |
|  | **Risk of bias judgement** | | | **Low** |  |
| **Bias due to deviations from intended interventions** | 2.1.Were participants aware of their assigned intervention during the trial? | | | PY |  |
|  | 2.2.Were carers and people delivering the interventions aware of participants' assigned intervention during the trial? | | | Y |  |
|  | 2.3. If Y/PY/NI to 2.1 or 2.2: Were there deviations from the intended intervention that arose because of the experimental context? | | | PN |  |
|  | 2.4 If Y/PY to 2.3: Were these deviations likely to have affected the outcome? | | | NA |  |
|  | 2.5. If Y/PY/NI to 2.4: Were these deviations from intended intervention balanced between groups? | | | NA |  |
|  | 2.6 Was an appropriate analysis used to estimate the effect of assignment to intervention? | | | NI |  |
|  | 2.7 If N/PN/NI to 2.6: Was there potential for a substantial impact (on the result) of the failure to analyse participants in the group to which they were randomized? | | | N |  |
|  | **Risk of bias judgement** | | | **Some concerns** |  |
| **Bias due to missing outcome data** | 3.1 Were data for this outcome available for all. or nearly all. participants randomized? | | | N | 2 patients (one in test and one in control group) were excluded from the study and from the analysis |
|  | 3.2 If N/PN/NI to 3.1: Is there evidence that result was not biased by missing outcome data? | | | PN |  |
|  | 3.3 If N/PN to 3.2: Could missingness in the outcome depend on its true value? | | | N |  |
|  | 3.4 If Y/PY/NI to 3.3: Is it likely that missingness in the outcome depended on its true value? | | | NA |  |
|  | **Risk of bias judgement** | | | **Low** |  |
| **Bias in measurement of the outcome** | 4.1 Was the method of measuring the outcome inappropriate? | | | N |  |
|  | 4.2 Could measurement or ascertainment of the outcome have differed between intervention groups? | | | PN |  |
|  | 4.3 Were outcome assessors aware of the intervention received by study participants? | | | PY |  |
|  | 4.4 If Y/PY/NI to 4.3: Could assessment of the outcome have been influenced by knowledge of intervention received? | | | NI |  |
|  | 4.5 If Y/PY/NI to 4.4: Is it likely that assessment of the outcome was influenced by knowledge of intervention received? | | | PN |  |
|  | **Risk of bias judgement** | | | **Some concerns** |  |
| **Bias in selection of the reported result** | 5.1 Were the data that produced this result analysed in accordance with a pre-specified analysis plan that was finalized before unblinded outcome data were available for analysis? | | | Y |  |
|  | 5.2 ... multiple eligible outcome measurements (e.g. scales. definitions. time points) within the outcome domain? | | | N |  |
|  | 5.3 ... multiple eligible analyses of the data? | | | N |  |
|  | **Risk of bias judgement** | | | **Low** |  |
| **Overall bias** | **Risk of bias judgement** | | | **Some concerns** |  |
|  |  |  |  |  |  |
| **Rotundo 2019^63^** |  |  |  |  |  |
| **Domain** | **Signalling question** | | | **Response** | **Comments** |
| **Bias arising from the randomization process** | 1.1 Was the allocation sequence random? | | | Y | Block randomization. envelopes openened after flap elevation |
|  | 1.2 Was the allocation sequence concealed until participants were enrolled and assigned to interventions? | | | Y |  |
|  | 1.3 Did baseline differences between intervention groups suggest a problem with the randomization process? | | | N |  |
|  | **Risk of bias judgement** | | | **Low** |  |
| **Bias due to deviations from intended interventions** | 2.1.Were participants aware of their assigned intervention during the trial? | | | Y |  |
|  | 2.2.Were carers and people delivering the interventions aware of participants' assigned intervention during the trial? | | | Y |  |
|  | 2.3. If Y/PY/NI to 2.1 or 2.2: Were there deviations from the intended intervention that arose because of the experimental context? | | | PN |  |
|  | 2.4 If Y/PY to 2.3: Were these deviations likely to have affected the outcome? | | | NA |  |
|  | 2.5. If Y/PY/NI to 2.4: Were these deviations from intended intervention balanced between groups? | | | NA |  |
|  | 2.6 Was an appropriate analysis used to estimate the effect of assignment to intervention? | | | NI |  |
|  | 2.7 If N/PN/NI to 2.6: Was there potential for a substantial impact (on the result) of the failure to analyse participants in the group to which they were randomized? | | | N |  |
|  | **Risk of bias judgement** | | | **Some concerns** |  |
| **Bias due to missing outcome data** | 3.1 Were data for this outcome available for all. or nearly all. participants randomized? | | | Y |  |
|  | 3.2 If N/PN/NI to 3.1: Is there evidence that result was not biased by missing outcome data? | | | NA |  |
|  | 3.3 If N/PN to 3.2: Could missingness in the outcome depend on its true value? | | | NA |  |
|  | 3.4 If Y/PY/NI to 3.3: Is it likely that missingness in the outcome depended on its true value? | | | NA |  |
|  | **Risk of bias judgement** | | | **Low** |  |
| **Bias in measurement of the outcome** | 4.1 Was the method of measuring the outcome inappropriate? | | | N |  |
|  | 4.2 Could measurement or ascertainment of the outcome have differed between intervention groups? | | | PN |  |
|  | 4.3 Were outcome assessors aware of the intervention received by study participants? | | | Y |  |
|  | 4.4 If Y/PY/NI to 4.3: Could assessment of the outcome have been influenced by knowledge of intervention received? | | | NI |  |
|  | 4.5 If Y/PY/NI to 4.4: Is it likely that assessment of the outcome was influenced by knowledge of intervention received? | | | PN |  |
|  | **Risk of bias judgement** | | | **Some concerns** |  |
| **Bias in selection of the reported result** | 5.1 Were the data that produced this result analysed in accordance with a pre-specified analysis plan that was finalized before unblinded outcome data were available for analysis? | | | Y |  |
|  | 5.2 ... multiple eligible outcome measurements (e.g. scales. definitions. time points) within the outcome domain? | | | N |  |
|  | 5.3 ... multiple eligible analyses of the data? | | | N |  |
|  | **Risk of bias judgement** | | | **Low** |  |
| **Overall bias** | **Risk of bias judgement** | | | **Some concerns** |  |
|  |  |  |  |  |  |
| **Potey 2019^51^** |  |  |  |  |  |
| **Domain** | **Signalling question** | | | **Response** | **Comments** |
| **Bias arising from the randomization process** | 1.1 Was the allocation sequence random? | | | Y |  |
|  | 1.2 Was the allocation sequence concealed until participants were enrolled and assigned to interventions? | | | NI |  |
|  | 1.3 Did baseline differences between intervention groups suggest a problem with the randomization process? | | | N |  |
|  | **Risk of bias judgement** | | | **Some concerns** |  |
| **Bias due to deviations from intended interventions** | 2.1.Were participants aware of their assigned intervention during the trial? | | | PY |  |
|  | 2.2.Were carers and people delivering the interventions aware of participants' assigned intervention during the trial? | | | Y |  |
|  | 2.3. If Y/PY/NI to 2.1 or 2.2: Were there deviations from the intended intervention that arose because of the experimental context? | | | PN |  |
|  | 2.4 If Y/PY to 2.3: Were these deviations likely to have affected the outcome? | | | NA |  |
|  | 2.5. If Y/PY/NI to 2.4: Were these deviations from intended intervention balanced between groups? | | | NA |  |
|  | 2.6 Was an appropriate analysis used to estimate the effect of assignment to intervention? | | | NI |  |
|  | 2.7 If N/PN/NI to 2.6: Was there potential for a substantial impact (on the result) of the failure to analyse participants in the group to which they were randomized? | | | N |  |
|  | **Risk of bias judgement** | | | **Some concerns** |  |
| **Bias due to missing outcome data** | 3.1 Were data for this outcome available for all. or nearly all. participants randomized? | | | Y |  |
|  | 3.2 If N/PN/NI to 3.1: Is there evidence that result was not biased by missing outcome data? | | | NA |  |
|  | 3.3 If N/PN to 3.2: Could missingness in the outcome depend on its true value? | | | NA |  |
|  | 3.4 If Y/PY/NI to 3.3: Is it likely that missingness in the outcome depended on its true value? | | | NA |  |
|  | **Risk of bias judgement** | | | **Low** |  |
| **Bias in measurement of the outcome** | 4.1 Was the method of measuring the outcome inappropriate? | | | N |  |
|  | 4.2 Could measurement or ascertainment of the outcome have differed between intervention groups? | | | PN |  |
|  | 4.3 Were outcome assessors aware of the intervention received by study participants? | | | PY |  |
|  | 4.4 If Y/PY/NI to 4.3: Could assessment of the outcome have been influenced by knowledge of intervention received? | | | NI |  |
|  | 4.5 If Y/PY/NI to 4.4: Is it likely that assessment of the outcome was influenced by knowledge of intervention received? | | | PN |  |
|  | **Risk of bias judgement** | | | **Some concerns** |  |
| **Bias in selection of the reported result** | 5.1 Were the data that produced this result analysed in accordance with a pre-specified analysis plan that was finalized before unblinded outcome data were available for analysis? | | | Y |  |
|  | 5.2 ... multiple eligible outcome measurements (e.g. scales. definitions. time points) within the outcome domain? | | | N |  |
|  | 5.3 ... multiple eligible analyses of the data? | | | N |  |
|  | **Risk of bias judgement** | | | **Low** |  |
| **Overall bias** | **Risk of bias judgement** | | | **Some concerns** |  |
|  |  |  |  |  |  |
| **Rajeswari 2021^64^** |  |  |  |  |  |
| **Domain** | **Signalling question** | | | **Response** | **Comments** |
| **Bias arising from the randomization process** | 1.1 Was the allocation sequence random? | | | Y | Coin toss. subsequent surgery aftert 1 month. there is no evidence provided to confirm concealment |
|  | 1.2 Was the allocation sequence concealed until participants were enrolled and assigned to interventions? | | | NI |  |
|  | 1.3 Did baseline differences between intervention groups suggest a problem with the randomization process? | | | N |  |
|  | **Risk of bias judgement** | | | **Some concerns** |  |
| **Bias due to deviations from intended interventions** | 2.1.Were participants aware of their assigned intervention during the trial? | | | PY |  |
|  | 2.2.Were carers and people delivering the interventions aware of participants' assigned intervention during the trial? | | | Y |  |
|  | 2.3. If Y/PY/NI to 2.1 or 2.2: Were there deviations from the intended intervention that arose because of the experimental context? | | | PN |  |
|  | 2.4 If Y/PY to 2.3: Were these deviations likely to have affected the outcome? | | | NA |  |
|  | 2.5. If Y/PY/NI to 2.4: Were these deviations from intended intervention balanced between groups? | | | NA |  |
|  | 2.6 Was an appropriate analysis used to estimate the effect of assignment to intervention? | | | NI |  |
|  | 2.7 If N/PN/NI to 2.6: Was there potential for a substantial impact (on the result) of the failure to analyse participants in the group to which they were randomized? | | | PN |  |
|  | **Risk of bias judgement** | | | **Some concerns** |  |
| **Bias due to missing outcome data** | 3.1 Were data for this outcome available for all. or nearly all. participants randomized? | | | Y |  |
|  | 3.2 If N/PN/NI to 3.1: Is there evidence that result was not biased by missing outcome data? | | | NA |  |
|  | 3.3 If N/PN to 3.2: Could missingness in the outcome depend on its true value? | | | NA |  |
|  | 3.4 If Y/PY/NI to 3.3: Is it likely that missingness in the outcome depended on its true value? | | | NA |  |
|  | **Risk of bias judgement** | | | **Low** |  |
| **Bias in measurement of the outcome** | 4.1 Was the method of measuring the outcome inappropriate? | | | N |  |
|  | 4.2 Could measurement or ascertainment of the outcome have differed between intervention groups? | | | PN |  |
|  | 4.3 Were outcome assessors aware of the intervention received by study participants? | | | PY |  |
|  | 4.4 If Y/PY/NI to 4.3: Could assessment of the outcome have been influenced by knowledge of intervention received? | | | NI |  |
|  | 4.5 If Y/PY/NI to 4.4: Is it likely that assessment of the outcome was influenced by knowledge of intervention received? | | | PN |  |
|  | **Risk of bias judgement** | | | **Some concerns** |  |
| **Bias in selection of the reported result** | 5.1 Were the data that produced this result analysed in accordance with a pre-specified analysis plan that was finalized before unblinded outcome data were available for analysis? | | | Y |  |
|  | 5.2 ... multiple eligible outcome measurements (e.g. scales. definitions. time points) within the outcome domain? | | | N |  |
|  | 5.3 ... multiple eligible analyses of the data? | | | N |  |
|  | **Risk of bias judgement** | | | **Low** |  |
| **Overall bias** | **Risk of bias judgement** | | | **High** |  |
|  |  |  |  |  |  |
| **Ahmedbeyli 2019^52^** |  |  |  |  |  |
| **Domain** | **Signalling question** | | | **Response** | **Comments** |
| **Bias arising from the randomization process** | 1.1 Was the allocation sequence random? | | | Y |  |
|  | 1.2 Was the allocation sequence concealed until participants were enrolled and assigned to interventions? | | | Y |  |
|  | 1.3 Did baseline differences between intervention groups suggest a problem with the randomization process? | | | N |  |
|  | **Risk of bias judgement** | | | **Low** |  |
| **Bias due to deviations from intended interventions** | 2.1.Were participants aware of their assigned intervention during the trial? | | | Y |  |
|  | 2.2.Were carers and people delivering the interventions aware of participants' assigned intervention during the trial? | | | Y |  |
|  | 2.3. If Y/PY/NI to 2.1 or 2.2: Were there deviations from the intended intervention that arose because of the experimental context? | | | PN |  |
|  | 2.4 If Y/PY to 2.3: Were these deviations likely to have affected the outcome? | | | NA |  |
|  | 2.5. If Y/PY/NI to 2.4: Were these deviations from intended intervention balanced between groups? | | | NA |  |
|  | 2.6 Was an appropriate analysis used to estimate the effect of assignment to intervention? | | | NI |  |
|  | 2.7 If N/PN/NI to 2.6: Was there potential for a substantial impact (on the result) of the failure to analyse participants in the group to which they were randomized? | | | N |  |
|  | **Risk of bias judgement** | | | **Some concerns** |  |
| **Bias due to missing outcome data** | 3.1 Were data for this outcome available for all. or nearly all. participants randomized? | | | Y |  |
|  | 3.2 If N/PN/NI to 3.1: Is there evidence that result was not biased by missing outcome data? | | | NA |  |
|  | 3.3 If N/PN to 3.2: Could missingness in the outcome depend on its true value? | | | NA |  |
|  | 3.4 If Y/PY/NI to 3.3: Is it likely that missingness in the outcome depended on its true value? | | | NA |  |
|  | **Risk of bias judgement** | | | **Low** |  |
| **Bias in measurement of the outcome** | 4.1 Was the method of measuring the outcome inappropriate? | | | N |  |
|  | 4.2 Could measurement or ascertainment of the outcome have differed between intervention groups? | | | PN |  |
|  | 4.3 Were outcome assessors aware of the intervention received by study participants? | | | Y |  |
|  | 4.4 If Y/PY/NI to 4.3: Could assessment of the outcome have been influenced by knowledge of intervention received? | | | NI |  |
|  | 4.5 If Y/PY/NI to 4.4: Is it likely that assessment of the outcome was influenced by knowledge of intervention received? | | | PN |  |
|  | **Risk of bias judgement** | | | **Some concerns** |  |
| **Bias in selection of the reported result** | 5.1 Were the data that produced this result analysed in accordance with a pre-specified analysis plan that was finalized before unblinded outcome data were available for analysis? | | | Y |  |
|  | 5.2 ... multiple eligible outcome measurements (e.g. scales. definitions. time points) within the outcome domain? | | | N |  |
|  | 5.3 ... multiple eligible analyses of the data? | | | N |  |
|  | **Risk of bias judgement** | | | **Low** |  |
| **Overall bias** | **Risk of bias judgement** | | | **Some concerns** |  |
|  |  |  |  |  |  |
| **Chen 2023^53^** |  |  |  |  |  |
| **Domain** | **Signalling question** | | | **Response** | **Comments** |
| **Bias arising from the randomization process** | 1.1 Was the allocation sequence random? | | | Y |  |
|  | 1.2 Was the allocation sequence concealed until participants were enrolled and assigned to interventions? | | | Y |  |
|  | 1.3 Did baseline differences between intervention groups suggest a problem with the randomization process? | | | N |  |
|  | **Risk of bias judgement** | | | **Low** |  |
| **Bias due to deviations from intended interventions** | 2.1.Were participants aware of their assigned intervention during the trial? | | | Y |  |
|  | 2.2.Were carers and people delivering the interventions aware of participants' assigned intervention during the trial? | | | Y |  |
|  | 2.3. If Y/PY/NI to 2.1 or 2.2: Were there deviations from the intended intervention that arose because of the experimental context? | | | PN |  |
|  | 2.4 If Y/PY to 2.3: Were these deviations likely to have affected the outcome? | | | NA |  |
|  | 2.5. If Y/PY/NI to 2.4: Were these deviations from intended intervention balanced between groups? | | | NA |  |
|  | 2.6 Was an appropriate analysis used to estimate the effect of assignment to intervention? | | | NI |  |
|  | 2.7 If N/PN/NI to 2.6: Was there potential for a substantial impact (on the result) of the failure to analyse participants in the group to which they were randomized? | | | PN |  |
|  | **Risk of bias judgement** | | | **Some concerns** |  |
| **Bias due to missing outcome data** | 3.1 Were data for this outcome available for all. or nearly all. participants randomized? | | | Y |  |
|  | 3.2 If N/PN/NI to 3.1: Is there evidence that result was not biased by missing outcome data? | | | NA |  |
|  | 3.3 If N/PN to 3.2: Could missingness in the outcome depend on its true value? | | | NA |  |
|  | 3.4 If Y/PY/NI to 3.3: Is it likely that missingness in the outcome depended on its true value? | | | NA |  |
|  | **Risk of bias judgement** | | | **Low** |  |
| **Bias in measurement of the outcome** | 4.1 Was the method of measuring the outcome inappropriate? | | | N |  |
|  | 4.2 Could measurement or ascertainment of the outcome have differed between intervention groups? | | | PN |  |
|  | 4.3 Were outcome assessors aware of the intervention received by study participants? | | | Y |  |
|  | 4.4 If Y/PY/NI to 4.3: Could assessment of the outcome have been influenced by knowledge of intervention received? | | | NI |  |
|  | 4.5 If Y/PY/NI to 4.4: Is it likely that assessment of the outcome was influenced by knowledge of intervention received? | | | PN |  |
|  | **Risk of bias judgement** | | | **Some concerns** |  |
| **Bias in selection of the reported result** | 5.1 Were the data that produced this result analysed in accordance with a pre-specified analysis plan that was finalized before unblinded outcome data were available for analysis? | | | Y |  |
|  | 5.2 ... multiple eligible outcome measurements (e.g. scales. definitions. time points) within the outcome domain? | | | N |  |
|  | 5.3 ... multiple eligible analyses of the data? | | | N |  |
|  | **Risk of bias judgement** | | | **Low** |  |
| **Overall bias** | **Risk of bias judgement** | | | **Some concerns** |  |

**Cairo et al. 2016^54^**

| **Domain** | **Signalling question** | **Response** | **Comments** |
| --- | --- | --- | --- |
| **Bias arising from the randomization process** | 1.1 Was the allocation sequence random? | Y | Randomization sealed and opaque envelopes opened after flap elevation |
|  | 1.2 Was the allocation sequence concealed until participants were enrolled and assigned to interventions? | Y |  |
|  | 1.3 Did baseline differences between intervention groups suggest a problem with the randomization process? | PN |  |
|  | **Risk of bias judgement** | **Low** |  |
| **Bias due to deviations from intended interventions** | 2.1.Were participants aware of their assigned intervention during the trial? | Y |  |
|  | 2.2.Were carers and people delivering the interventions aware of participants' assigned intervention during the trial? | Y |  |
|  | 2.3. If Y/PY/NI to 2.1 or 2.2: Were there deviations from the intended intervention that arose because of the experimental context? | PN |  |
|  | 2.4 If Y/PY to 2.3: Were these deviations likely to have affected the outcome? | NA |  |
|  | 2.5. If Y/PY/NI to 2.4: Were these deviations from intended intervention balanced between groups? | NA |  |
|  | 2.6 Was an appropriate analysis used to estimate the effect of assignment to intervention? | PN |  |
|  | 2.7 If N/PN/NI to 2.6: Was there potential for a substantial impact (on the result) of the failure to analyse participants in the group to which they were randomized? | PN |  |
|  | **Risk of bias judgement** | **Some concerns** |  |
| **Bias due to missing outcome data** | 3.1 Were data for this outcome available for all. or nearly all. participants randomized? | Y |  |
|  | 3.2 If N/PN/NI to 3.1: Is there evidence that result was not biased by missing outcome data? | NA |  |
|  | 3.3 If N/PN to 3.2: Could missingness in the outcome depend on its true value? | NA |  |
|  | 3.4 If Y/PY/NI to 3.3: Is it likely that missingness in the outcome depended on its true value? | NA |  |
|  | **Risk of bias judgement** | **Low** |  |
| **Bias in measurement of the outcome** | 4.1 Was the method of measuring the outcome inappropriate? | N |  |
|  | 4.2 Could measurement or ascertainment of the outcome have differed between intervention groups? | N |  |
|  | 4.3 Were outcome assessors aware of the intervention received by study participants? | Y |  |
|  | 4.4 If Y/PY/NI to 4.3: Could assessment of the outcome have been influenced by knowledge of intervention received? | PN |  |
|  | 4.5 If Y/PY/NI to 4.4: Is it likely that assessment of the outcome was influenced by knowledge of intervention received? | NA |  |
|  | **Risk of bias judgement** | **Low** |  |
| **Bias in selection of the reported result** | 5.1 Were the data that produced this result analysed in accordance with a pre-specified analysis plan that was finalized before unblinded outcome data were available for analysis? | Y |  |
|  | 5.2 ... multiple eligible outcome measurements (e.g. scales. definitions. time points) within the outcome domain? | N |  |
|  | 5.3 ... multiple eligible analyses of the data? | N |  |
|  | **Risk of bias judgement** | **Low** |  |
| **Overall bias** | **Risk of bias judgement** | **Some concerns** |  |

**Tonetti et al. 2018 ^55^**

| **Domain** | **Signalling question** | **Response** | **Comments** |
| --- | --- | --- | --- |
| **Bias arising from the randomization process** | 1.1 Was the allocation sequence random? | Y | Random permuted blocks. block size of 4 with minimization for cigarette smoking. Opaque envelopes opened upon completition of the common portion of the surgery |
|  | 1.2 Was the allocation sequence concealed until participants were enrolled and assigned to interventions? | Y |  |
|  | 1.3 Did baseline differences between intervention groups suggest a problem with the randomization process? | PN |  |
|  | **Risk of bias judgement** | **Low** |  |
| **Bias due to deviations from intended interventions** | 2.1.Were participants aware of their assigned intervention during the trial? | Y |  |
|  | 2.2.Were carers and people delivering the interventions aware of participants' assigned intervention during the trial? | Y |  |
|  | 2.3. If Y/PY/NI to 2.1 or 2.2: Were there deviations from the intended intervention that arose because of the experimental context? | PN |  |
|  | 2.4 If Y/PY to 2.3: Were these deviations likely to have affected the outcome? | NA |  |
|  | 2.5. If Y/PY/NI to 2.4: Were these deviations from intended intervention balanced between groups? | NA |  |
|  | 2.6 Was an appropriate analysis used to estimate the effect of assignment to intervention? | PN |  |
|  | 2.7 If N/PN/NI to 2.6: Was there potential for a substantial impact (on the result) of the failure to analyse participants in the group to which they were randomized? | PN |  |
|  | **Risk of bias judgement** | **Some concerns** |  |
| **Bias due to missing outcome data** | 3.1 Were data for this outcome available for all. or nearly all. participants randomized? | Y |  |
|  | 3.2 If N/PN/NI to 3.1: Is there evidence that result was not biased by missing outcome data? | NA |  |
|  | 3.3 If N/PN to 3.2: Could missingness in the outcome depend on its true value? | NA |  |
|  | 3.4 If Y/PY/NI to 3.3: Is it likely that missingness in the outcome depended on its true value? | NA |  |
|  | **Risk of bias judgement** | **Low** |  |
| **Bias in measurement of the outcome** | 4.1 Was the method of measuring the outcome inappropriate? | N |  |
|  | 4.2 Could measurement or ascertainment of the outcome have differed between intervention groups? | PN |  |
|  | 4.3 Were outcome assessors aware of the intervention received by study participants? | Y |  |
|  | 4.4 If Y/PY/NI to 4.3: Could assessment of the outcome have been influenced by knowledge of intervention received? | PN |  |
|  | 4.5 If Y/PY/NI to 4.4: Is it likely that assessment of the outcome was influenced by knowledge of intervention received? | NA |  |
|  | **Risk of bias judgement** | **Low** |  |
| **Bias in selection of the reported result** | 5.1 Were the data that produced this result analysed in accordance with a pre-specified analysis plan that was finalized before unblinded outcome data were available for analysis? | Y |  |
|  | 5.2 ... multiple eligible outcome measurements (e.g. scales. definitions. time points) within the outcome domain? | N |  |
|  | 5.3 ... multiple eligible analyses of the data? | N |  |
|  | **Risk of bias judgement** | **Low** |  |
| **Overall bias** | **Risk of bias judgement** | **Some concerns** |  |

**Table S6. Descriptive demographic data and characteristics of the studies**

| **id** | **study** | **country** | **study_design** | **study_type** | **Setting** | **Funding** | **Follow-up (days)** | **Technique** | **Adjunctive** | **type_adjunctive** | **mean_age (years)** | **sd_age (years)** | **total n° subjects** | **female (n)** | **male (n)** | **total n° recessions** | **location** | **type_recession** | **smoking** | **duration_surg_mean (min)** | **sd_duration (min)** | **painkillers** | **antibiotics** |
| --- | --- | --- | --- | --- | --- | --- | --- | --- | --- | --- | --- | --- | --- | --- | --- | --- | --- | --- | --- | --- | --- | --- | --- |
| 1 | Gonzalez-Febles 2023_CAF_ATG^30^ | Spain | parallel | RCT | university | open access CRUE-CSIC | 180 | CAF | ATG | CTG | 34.7 | 9.5 | 29 | 10 | 5 | 83 | BOTH | RT1. RT2 | no | 63.9 | 18 | yes | NA |
|  | Gonzalez-Febles 2023_TUN_ATG^30^ | Spain | parallel | RCT | university | open access CRUE-CSIC | 180 | TUN | ATG | CTG | 34 | 9.5 |  | 12 | 3 |  | BOTH | RT1. RT2 | no | 44.5 | 6.4 | yes | NA |
| 2 | Zangrando 2020_CAF_SCTG^31^ | Brazil | split-mouth | RCT | university | university (government) | 180 | CAF | ATG | CTG-deepithelialized | 36.94 | NA | 21 | 13 | 8 | 84 | NA | RT1 | no | NA | NA | yes | NA |
|  | Zangrando 2020_CAF_SCTG^31^ | Brazil | split-mouth | RCT | university | university (government) | 180 | CAF | ATG | CTG- subepithelial | 36.94 | NA |  | 13 | 8 |  | NA | RT1 | no | NA | NA | yes | NA |
| 3 | Rotundo 2019_CAF_NO_ADJ^63^ | Italy | parallel | RCT | private office | NA | 365 | CAF | NO_ADJ | / | 38.1 | 7.3 | 24 | 10 | 2 | 61 | MAX | RT1 | no | 36.1 | 4.6 | yes | NA |
|  | Rotundo 2019_CAF_GS^63^ | Italy | parallel | RCT | private office | NA | 365 | CAF | GS | CM | 31.4 | 4.9 |  | 9 | 3 |  | MAX | RT1 | no | 47.3 | 5.8 | yes | NA |
| 4 | Rotundo 2021_CAF_NO_ADJ^32^ | Italy | parallel | RCT | private office | NA | 365 | CAF | NO_ADJ | / | 38.1 | 7.3 | 24 | 10 | 2 | 61 | MAX | RT1 | no | 36.1 | 4.6 | yes | NA |
|  | Rotundo 2021_CAF_GS^32^ | Italy | parallel | RCT | private office | NA | 365 | CAF | GS | CM | 31.4 | 4.9 |  | 9 | 3 |  | MAX | RT1 | no | 47.3 | 5.8 | yes | NA |
| 5 | Rakasevic 2020_TUN_GS^33^ | Serbia | split-mouth | RCT | university | industry | 365 | TUN | GS | CTG | 30.5 | 7.9 | 20 | 11 | 9 | 114 | BOTH | RT1 | yes (n=5) | NA |  | yes | yes |
|  | Rakasevic 2020_TUN_ATG^33^ | Serbia | split-mouth | RCT | university | industry | 365 | TUN | ATG | CM | 30.5 | 7.9 |  | 11 | 9 |  | BOTH | RT1 | yes (n=5) | NA |  | yes | yes |
| 6 | Lakshmi 2023_TUN_ATG^1^ | India | parallel | RCT | university | NA | 180 | TUN | ATG | CTG | 37.39 | 6.4 | 28 | NA | NA | 64 | NA | RT1 | yes | 48.85 | 1.57 | yes | NA |
|  | Lakshmi 2023_TUN_GS^1^ | India | parallel | RCT | university | NA | 180 | TUN | GS | CM | 37.91 | 6.91 |  | NA | NA |  | NA | RT1 | yes | 26.23 | 1.87 | yes | NA |
| 7 | Carrera 2023_TUN_ATG^34^ | Brazil | split-mouth | RCT | university | university (government) | 480 | TUN | ATG | CTG | 35 | NA | 14 | 7 | 7 | 72 | MAX | RT1 | no | 53.36 | 11.55 | yes | yes |
|  | Carrera 2023_TUN_BA^34^ | Brazil | split-mouth | RCT | university | university (government) | 480 | TUN | BA | PRF | 35 | NA |  | 7 | 7 |  | MAX | RT1 | no | 51.36 | 13.47 | yes | yes |
| 8 | Elmahdi 2022_TUN_ADM^59^ | Egypt | parallel | RCT | university | university | 270 | TUN | GS | ADM | 42 | 6 | 24 | 10 | 2 | 67 | BOTH | RT1 | no | NA | NA | yes | yes |
|  | Elmahdi 2022_TUN_ATG^59^ | Egypt | parallel | RCT | university | university | 270 | TUN | ATG | CTG | 39 | 8 |  | 9 | 3 |  | BOTH | RT1 | no | NA | NA | yes | yes |
| 9 | Ylmaz 2022_FGG^60^ | Turkey | parallel | RCT | university | NA | 365 | FGG | NO_ADJ | / | 40.55 | 10.36 | 50 | 22 | 3 | 140 | MAN | RT1. RT2 | no | NA | NA | yes | NA |
|  | Ylmaz 2022_TUN_ATG^60^ | Turkey | parallel | RCT | university | NA | 365 | TUN | ADJ | CTG | 43.55 | 11.36 |  | 14 | 11 |  | MAN | RT1. RT2 | no | NA | NA | yes | NA |
| 10 | Bakhishov 2021_TUN_ATG^35^ | Turkey | parallel | RCT | university | NA | 365 | TUN | ATG | CTG- subepithelial | 40.57 | 12.17 | 27 | 4 | 10 | 61 | BOTH | RT1 | no | 66.07 | 10.59 | yes | NA |
|  | Bakhishov 2021_TUN_ATG^35^ | Turkey | parallel | RCT | university | NA | 365 | TUN | ATG | CTG-deepithelialized | 43.5 | 9.26 |  | 8 | 5 |  | BOTH | RT1 | no | 70.42 | 12.87 | yes | NA |
| 11 | Meza-Mauricio 2021_CAF_ATG^36^ | Brazil | parallel | RCT | university | NA | 365 | CAF | ARG | CTG- subepithelial | 38.1 | 7.2 | 41 | 12 | 8 | 130 | MAX | RT1 | no | 48.8 | 15.06 | yes | NA |
|  | Meza-Mauricio 2021_CAF_GS^36^ | Brazil | parallel | RCT | university | NA | 365 | CAF | GS | CM | 36.3 | 6.1 |  | 12 | 9 |  | MAX | RT1 | no | 36 | 8.1 | yes | NA |
| 12 | Gorski 2022_TUN_ATG^37^ | Poland | split-mouth | RCT | university | NA | 365 | TUN | ATG | CTG | 28.87 | 4.46 | 20 | 11 | 9 | 142 | BOTH | RT1. RT2 | no | NA | NA | yes | NA |
|  | Gorski 2022_TUN_ATG_EDTA^37^ | Poland | split-mouth | RCT | university | NA | 365 | TUN | ATG_EDTA | CTG_EDTA | 28.87 | 4.46 |  | 11 | 9 |  | BOTH | RT1. RT2 | no | NA | NA | yes | NA |
| 13 | Tavelli 2022_CAF_GS^39^ | USA | parallel | RCT | university | both | 180 | CAF | GS | CM | 40.9 | 12.3 | 30 | 8 | 7 | 91 | NA | RT1 | yes | NA | NA | yes | yes |
|  | Tavelli 2022_CAF_GS_BA^39^ | USA | parallel | RCT | university | both | 180 | CAF | GS_BA | rhPDGF + CM | 36 | 11 |  | 11 | 4 |  | NA | RT1 | no | NA | NA | yes | yes |
| 14 | Aroca 2013_TUN_GS^61^ | Hungary | split-mouth | RCT | university | NA | 365 | TUN | GS | CM | NA | NA | 22 | NA | NA | 156 | BOTH | RT1 | no | 42.5 | 4.8 | yes | yes |
|  | Aroca 2013_TUN_ATG^61^ | Hungary | split-mouth | RCT | university | NA | 365 | TUN | ATG | CTG- subepithelial | NA | NA |  | NA | NA |  | BOTH | RT1 | no | 58.6 | 6.6 | yes | yes |
| 15 | Zucchelli 2014_CAF_NO_ADJ^40^ | Italy | parallel | RCT | university | university | 365 | CAF | NO_ADG | / | 34.2 | 6.2 | 50 | 15 | 10 | 149 | MAX | RT1 | yes | 29.8 | 3.2 | yes | NA |
|  | Zucchelli 2014_CAF_ATG^40^ | Italy | parallel | RCT | university | university | 365 | CAF | ATG | CTG-deepithelialized | 33.2 | 7.4 |  | 14 | 11 |  | MAX | RT1 | yes | 40.1 | 6.8 | yes | NA |
| 16 | Fernández-Jiménez 2023_CAF_ATG^62^ | Spain | parallel | RCT | university | university | 365 | CAF | ATG | CTG- subepithelial | 61.16 | 10.37 | 24 | 6 | 6 | 84 | BOTH | RT2 | yes | NA | NA | yes | yes |
|  | Fernández-Jiménez 2023_VISTA_ATG^62^ | Spain | parallel | RCT | university | university | 365 | VISTA | ATG | CTG- subepithelial | 55.26 | 7.89 |  | 8 | 4 |  | BOTH | RT2 | yes | NA | NA | yes | yes |
| 17 | Gorski 2023_TUN_ATG^41^ | Poland | split-mouth | RCT | university | NA | 180 | TUN | ATG | CTG- subepithelial | NA | NA | 20 | 19 | 5 | 142 | BOTH | RT1. RT2 | no | NA | NA | yes | NA |
|  | Gorski 2023_TUN_ATG_BA^41^ | Poland | split-mouth | RCT | university | NA | 180 | TUN | ATG_BA | CTG- subepithelial+Hyaluronic acid | NA | NA |  | 19 | 5 |  | BOTH | RT1. RT2 | no | NA | NA | yes | NA |
| 18 | Gorski 2020_TUN_ATG^42^ | Poland | split-mouth | RCT | university | NA | 180 | TUN | ATG | CTG- subepithelial | 28.35 | 4.51 | 20 | 13 | 7 | 150 | BOTH | RT1. RT2 | no | NA | NA | yes | NA |
|  | Gorski 2020_TUN_ATG_BA^42^ | Poland | split-mouth | RCT | university | NA | 180 | TUN | ATG_BA | CTG_EMD | 28.35 | 4.51 |  | 13 | 7 |  | BOTH | RT1. RT2 | no | NA | NA | yes | NA |
| 19 | Zucchelli 2009_CAF_NO_ADJ^43^ | Italy | parallel | RCT | university | NA | 365 | CAF+vertical incisions | NO_ADG | / | 33.2 | 5.8 | 32 | 10 | 6 | 92 | MAX | RT1 | yes | 34.6 | 3.9 | yes | NA |
|  | Zucchelli 2009_CAF_NO_ADJ^43^ | Italy | parallel | RCT | university | NA | 365 | CAF | NO_ADG | / | 32.2 | 6.4 |  | 10 | 6 |  | MAX | RT1 | yes | 28.7 | 2.2 | yes | NA |
| 20 | Cieślik-Wegemund 2016_TUN_ATG^38^ | Poland | parallel | RCT | university | NA | 180 | TUN | ATG | / | NA | NA | 28 | 12 | 2 | 106 | BOTH | RT1 | no | NA | NA | NA | no |
|  | Cieślik-Wegemund 2016_TUN_GS^38^ | Poland | parallel | RCT | university | NA | 180 | TUN | GS | CM | NA | NA |  | 7 | 7 |  | BOTH | RT1 | no | NA | NA | NA | yes |
| 21 | Nahas 2020_CAF_ATG^44^ | Brazil | split-mouth | RCT | university | both | 365 | CAF | ATG | CTG- subepithelial | 32.7 | 8.1 | 15 | 8 | 7 | 82 | NA | RT1 (Miller 1) | no | 47.7 | 6.1 | yes | NA |
|  | Nahas 2020_CAF_GS^44^ | Brazil | split-mouth | RCT | university | both | 365 | CAF | GS | CM | 32.7 | 8.1 |  | 8 | 7 |  | NA | RT 1(Miller 1) | no | 31.3 | 4.3 | yes | NA |
| 22 | Kuka 2018_CAF_NO_ADJ^45^ | Turkey | parallel | RCT | university | NA | 365 | CAF | NO_ADG | / | NA | NA | 24 | NA | NA | 52 | BOTH | RT1 (Miller 1) | no | NA | NA | yes | yes |
|  | Kuka 2018_CAF_BA^45^ | Turkey | parallel | RCT | university | NA | 365 | CAF | BA | PRF | NA | NA |  | NA | NA |  | BOTH | RT1 (Miller 1) | no | NA | NA | yes | yes |
| 23 | Trivedi 2023_PST_NO_ADJ^46^ | India | split-mouth | RCT | university | NA | 365 | PST | NO_ADG | / | NA | NA | 25 | 10 | 15 | 165 | MAX | RT1 | no | NA | NA | yes | yes |
|  | Trivedi 2023_PST_BA^46^ | India | split-mouth | RCT | university | NA | 365 | PST | BA | A-PRF | NA | NA |  | 10 | 15 |  | MAX | RT1 | no | NA | NA | yes | yes |
| 24 | Ahmedbeyli 2014_CAF_NO_ADJ^47^ | Turkey | parallel | RCT | university | university | 365 | CAF | NO_ADG | / | NA | NA | 24 | nr | NA | 48 | MAX | RT1 (Miller 1) | no | NA | NA | yes | yes |
|  | Ahmedbeyli 2014_CAF_GS^47^ | Turkey | parallel | RCT | university | university | 365 | CAF | GS | ADM | NA | NA |  | NA | NA |  | MAX | RT1 (Miller 1) | no | NA | NA | yes | yes |
| 25 | Ozcelik 2011_CAF_NO_ADJ^48^ | Turkey | parallel | RCT | university | university | 180 | CAF | NO_ADG | / | NA | NA | 41 | NA | NA | 155 | MAX | RT1 | no | NA | NA | yes | NA |
|  | Ozcelik 2011_CAF_OB^48^ | Turkey | parallel | RCT | university | university | 180 | CAF | OB | / | NA | NA |  | NA | NA |  | MAX | RT1 | no | NA | NA | yes | NA |
| 26 | Pietruska 2018_TUN_ATG^49^ | Poland | split-mouth | RCT | university | university | 365 | TUN | ATG | CTG-deepithelialized | NA | NA | 20 | 13 | 7 | 91 | MAN | RT1 | no | NA | NA | yes | NA |
|  | Pietruska 2018_TUN_GS^49^ | Poland | split-mouth | RCT | university | university | 365 | TUN | GS | CM | NA | NA |  | 13 | 7 |  | MAN | RT1 | no | NA | NA | yes | NA |
| 27 | Santamaria 2022_CAF_ATG^50^ | Brazil | parallel | RCT | university | university (government) | 180 | CAF | ATG | CTG- subepithelial | 53.42 | 8.1 | 38 | 11 | 8 | 78 | BOTH | RT1 | no | 57.2 | 6.3 | yes | yes |
|  | Santamaria 2022_CAF_GS^50^ | Brazil | parallel | RCT | university | university (government) | 180 | CAF | GS | CM | 50.2 | 8 |  | 14 | 5 |  | BOTH | RT1 | no | 37.3 | 8 | yes | yes |
| 28 | Potey 2019_CAF_OB^51^ | India | parallel | RCT | university | NA | 180 | CAF | OB | / | NA | NA | 20 | NA | NA | 150 | MAX | RT1 | no | NA | NA | yes | yes |
|  | Potey 2019_CAF_OB_BA^51^ | India | parallel | RCT | university | NA | 180 | CAF | OB_BA | A-PRF | NA | NA |  | NA | NA |  | MAX | RT1 | no | NA | NA | yes | yes |
| 29 | Rajeswari 2021_CAF_BA^64^ | India | split-mouth | RCT | university | NA | 540 | CAF | BA | A-PRF | 34.2 | 9.2 | 16 | 10 | 6 | 107 | NA | RT1 | no | NA | NA | yes | yes |
|  | Rajeswari 2021_VISTA_BA^64^ | India | split-mouth | RCT | university | NA | 540 | VISTA | BA | A-PRF | 34.2 | 9.2 |  | 10 | 6 |  | NA | RT1 | no | NA | NA | yes | yes |
| 30 | Ahmedbeyli 2019_CAF_GS^52^ | Turkey | parallel | RCT | private office | self-funded | 365 | CAF +vertical incisions | GS | ADM | NA | NA | 22 | NA | NA | 55 | MAX | RT1 | no | NA | NA | yes | yes |
|  | Ahmedbeyli 2019_CAF_GS^52^ | Turkey | parallel | RCT | private office | self-funded | 365 | CAF | GS | ADM | NA | NA |  | NA | NA |  | MAX | RT1 | no | NA | NA | yes | yes |
| 31 | Chen 2023_TUN_ATG^53^ | China | parallel | RCT | university | NA | 365 | TUN | ATG | CTG- subepithelial | 37.35 | 10.99 | 24 | 7 | 5 | 59 | BOTH | RT1 | no | NA | NA | yes | yes |
|  | Chen 2023_VISTA_ATG^53^ | China | parallel | RCT | university | NA | 365 | VISTA | ATG | CTG- subepithelial | 35.33 | 10.6 |  | 8 | 4 |  | BOTH | RT1 | no | NA | NA | yes | yes |
| 32 | Cairo 2016_CAF_NO_ADJ^54^ | Italy | parallel | RCT | university | university | 365 | CAF | NO_ADG | / | 35.1 | 10.4 | 32 | 10 | 6 | 74 | MAX | RT1 | yes (n=3) | 54.7 | 4.2 | yes | NA |
|  | Cairo 2016_CAF_ATG^54^ | Italy | parallel | RCT | university | university | 365 | CAF | ATG | CTG- subepithelial | 33.4 | 7 |  | 13 | 3 |  | MAX | RT1 | yes (n=2) | 79.4 | 5.6 | yes | NA |
| 33 | Tonetti 2018_CAF_CTG^55^ | Italy | parallel | RCT | university | both | 180 | CAF | ATG | CTG-deepithelialized | 39.1 | 10.5 | 187 | 61 | 34 | 485 | BOTH | RT1 | yes | 69.7 | 24.3 | yes | yes |
|  | Tonetti 2018_CAF_GS^55^ | Italy | parallel | RCT | university | both | 180 | CAF | GS | CM | 41.2 | 10 |  | 57 | 35 |  | BOTH | RT1 | yes | 53.2 | 17.7 | yes | yes |
| 34 | Pelekos 2019_CAF_CTG^56^ | China | parallel | RCT | university | both | 180 | CAF | ATG | CTG-deepithelialized | 39.1 | 10.5 | 183 | 61 | 34 | 475 | NA | RT1 | yes | 69.7 | 24.3 | yes | yes |
|  | Pelekos 2019_CAF_GS^56^ | China | parallel | RCT | university | both | 180 | CAF | GS | CM | 41.2 | 10 |  | 57 | 35 |  | NA | RT1 | yes | 53.2 | 17.7 | yes | yes |
| 35 | Tonetti 2021_CAF_ATG^57^ | Italy | parallel | RCT | university | both | 1080 | CAF | ATG | CTG-deepithelialized | 39.1 | 10.5 | 125 | 37 | 27 | 307 | BOTH | RT1 | yes | 69.7 | 24.3 | yes | yes |
|  | Tonetti 2021_CAF_GS^57^ | Italy | parallel | RCT | university | both | 1080 | CAF | GS | CM | 41.2 | 10 |  | 37 | 24 |  | BOTH | RT1 | yes | 53.2 | 17.7 | yes | yes |

CAF: coronally advanced flap; TUN: tunnel; VISTA: Vista; PST: Pinhole surgical technique; ATG: autogenous graft (any type of technique); CM: collagen membrane; ADM: allodermic membrane; BA: biologic agents (enamel matrix derivative, platelet rich fibrin, chorion membrane, hyaluronic acid, growth factors, recombinant human factors); FGG: free gingival graft; OB: orthodontic application; GS: gingival substitute; ADJ: adjunctives; RT: recession type; CTG: connective tissue graft; MAX: maxillary arc; MAN:mandibular arch; NA: not available

**Table S7. Summary of the meta-analysis of primary and secondary outcomes**

|  | **Technique** | **n** | **WME** | **95% CI** | | **p** | **I2 (%)** | **p** |
| --- | --- | --- | --- | --- | --- | --- | --- | --- |
|  |  |  |  | **Lower** | **Upper** |  |  |  |
| **Rec_baseline (mm)** | ***Overall*** | 60 | 2.66 | 2.49 | 2.83 | <0.001 | 97.4 | <0.001 |
|  | ***CAF*** | 30 | 3.00 | 2.84 | 3.15 | <0.001 | 93.8 | <0.001 |
|  | *With adjunctive* | 22 | 2.96 | 2.80 | 3.13 | <0.001 | 93.0 | <0.001 |
|  | *Without adjunctive* | 8 | 3.09 | 2.73 | 3.45 | <0.001 | 94.7 | <0.001 |
|  | ***TUN*** | 25 | 2.33 | 2.12 | 2.53 | <0.001 | 95.4 | <0.001 |
|  | ***FCG*** | 1 | 3.58 | 3.26 | 3.90 | <0.001 |  |  |
|  | ***VISTA*** | 2 | 2.58 | 2.05 | 3.11 | <0.001 | 90.5 | 0.001 |
|  | ***PST*** | 2 | 1.42 | 1.29 | 1.55 | <0.001 | 45.6 | 0.175 |
| **Mean_root_cover_pc(%)** | ***Overall*** | 52 | 82.59 | 71.33 | 93.86 | <0.001 | 99.8 | <0.001 |
|  | ***CAF*** | 24 | 86.53 | 83.31 | 89.75 | <0.001 | 92.9 | <0.001 |
|  | *With adjunctive* | 18 | 86.86 | 83.18 | 90.55 | <0.001 | 92.7 | <0.001 |
|  | *Without adjunctive* | 6 | 85.12 | 77.31 | 92.94 | <0.001 | 94.6 | <0.001 |
|  | ***TUN*** | 23 | 80.85 | 64.50 | 97.21 | <0.001 | 99.7 | <0.001 |
|  | ***FCG*** | 1 | 63.54 | 51.84 | 75.24 | <0.001 |  |  |
|  | ***VISTA*** | 2 | 83.50 | 66.17 | 100.00 | <0.001 | 79.6 | 0.027 |
|  | ***PST*** | 2 | 79.67 | 65.97 | 93.37 | <0.001 | 86.7 | 0.006 |
| **CRC (prevalence, %)** | ***Overall*** | 60 | 62.7 | 57.0 | 68.4 | <0.001 | 94.06 | <0.001 |
|  | ***CAF*** | 32 | 65.2 | 58.7 | 71.7 | <0.001 | 90.47 | <0.001 |
|  | *With adjunctive* | 24 | 65.5 | 58.1 | 72.9 | <0.001 | 90.59 | <0.001 |
|  | *Without adjunctive* | 8 | 64.0 | 49.9 | 78.1 | <0.001 | 90.48 | <0.001 |
|  | ***TUN*** | 23 | 61.0 | 50.0 | 71.9 | <0.001 | 96.30 | <0.001 |
|  | ***FGG*** | 1 | 10.0 | 0.7 | 19.3 |  |  |  |
|  | ***VISTA*** | 2 | 59.2 | 35.9 | 82.4 | <0.001 | 75.94 | 0.042 |
|  | ***PST*** | 2 | 67.4 | 51.9 | 82.8 | <0.001 | 78.96 | 0.029 |
| **REC_RED (mm)** | ***Overall*** | 62 | 2.22 | 2.11 | 2.32 | <0.001 | 99.2 | <0.001 |
|  | ***CAF*** | 32 | 2.58 | 2.49 | 2.67 | <0.001 | 98.6 | <0.001 |
|  | *With adjunctive* | 24 | 2.57 | 2.47 | 2.67 | <0.001 | 98.8 | <0.001 |
|  | *Without adjunctive* | 8 | 2.63 | 2.23 | 3.03 | <0.001 | 95.6 | <0.001 |
|  | ***TUN*** | 25 | 1.86 | 1.66 | 2.07 | <0.001 | 95.6 | <0.001 |
|  | ***FCG*** | 1 | 2.29 | 1.88 | 2.70 | <0.001 |  |  |
|  | ***VISTA*** | 2 | 2.37 | 1.70 | 2.69 | 0.021 | 79.2 | 0.028 |
|  | ***PST*** | 2 | 1.13 | 0.83 | 1.43 | <0.001 | 94.0 | <0.001 |
| **KT_change (mm)** | ***Overall*** | 60 | 0.74 | 0.59 | 0.89 | <0.001 | 96.9 | <0.001 |
|  | ***CAF*** | 31 | 0.58 | 0.37 | 0.79 | <0.001 | 97.7 | <0.001 |
|  | *With adjunctive* | 23 | 0.68 | 0.42 | 0.94 | <0.001 | 98.0 | <0.001 |
|  | *Without adjunctive* | 8 | 0.33 | 0.09 | 0.57 | 0.007 | 93.1 | <0.001 |
|  | ***TUN*** | 24 | 0.79 | 0.55 | 1.03 | <0.001 | 94.4 | <0.001 |
|  | ***FCG*** | 1 | 4.43 | 3.84 | 5.02 |  |  | <0.001 |
|  | ***VISTA*** | 2 | 0.74 | 0.47 | 1.00 | 0.143 | 53.4 | <0.001 |
|  | ***PST*** | 2 | 1.14 | 0.45 | 1.82 | <0.001 | 96.7 | 0.001 |
| **GT_change (mm)** | ***Overall*** | 38 | 0.56 | 0.43 | 0.68 | <0.001 | 99.6 | <0.001 |
|  | ***CAF*** | 14 | 0.40 | 0.24 | 0.57 | <0.001 | 99.5 | <0.001 |
|  | *With adjunctive* | 10 | 0.59 | 0.50 | 0.67 | <0.001 | 92.9 | <0.001 |
|  | *Without adjunctive* | 4 | 0.03 | -0.026 | 0.09 | 0.296 | 91.6 | <0.001 |
|  | ***TUN*** | 22 | 0.65 | 0.54 | 0.77 | <0.001 | 98.5 | <0.001 |
|  | ***FCG*** | 1 | 0.72 | 0.62 | 0.82 | <0.001 |  |  |
|  | ***VISTA*** | 1 | 0.41 | 0.19 | 0.63 | <0.001 |  |  |
| **CAL_change (mm)** | ***Overall*** | 56 | 2.17 | 1.97 | 2.38 | <0.001 | 97.3 | <0.001 |
|  | ***CAF*** | 27 | 2.39 | 2.11 | 2.67 | <0.001 | 97.0 | <0.001 |
|  | *With adjunctive* | 19 | 2.32 | 1.97 | 2.67 | <0.001 | 97.0 | <0.001 |
|  | *Without adjunctive* | 8 | 2.54 | 2.05 | 3.04 | <0.001 | 97.2 | <0.001 |
|  | ***TUN*** | 24 | 2.01 | 1.73 | 2.29 | <0.001 | 96.9 | <0.001 |
|  | ***FCG*** | 1 | 2.05 | 1.54 | 2.56 | <0.001 |  |  |
|  | ***VISTA*** | 2 | 1.89 | 0.93 | 2.85 | <0.001 | 91.7 | 0.001 |
|  | ***PST*** | 2 | 1.54 | 1.08 | 2.00 | <0.001 | 90.0 | 0.002 |
| **Pain_VAS_10** | ***Overall*** | 18 | 2.67 | 2.28 | 3.06 | <0.001 | 99.3 | <0.001 |
|  | ***CAF*** | 8 | 3.75 | 1.27 | 6.24 | 0.003 | 98.7 | <0.001 |
|  | *With adjunctive* | 7 | 4.13 | 1.39 | 6.87 | 0.003 | 98.8 | <0.001 |
|  | *Without adjunctive* | 1 | 1.20 | -0.05 | 2.45 | 0.061 |  |  |
|  | ***TUN*** | 9 | 2.25 | 1.80 | 2.69 | <0.001 | 99.5 | <0.001 |
|  | ***VISTA*** | 1 | 3.17 | 1.01 | 5.33 | 0.004 |  |  |
| **Pain_VAS_100** | ***Overall*** | 10 | 24.34 | 16.46 | 32.22 | <0.001 | 98.4 | <0.001 |
|  | ***CAF*** | 5 | 45.75 | 33.41 | 58.08 | <0.001 | 92.3 | <0.001 |
|  | *With adjunctive* | 4 | 51.21 | 37.98 | 65.44 | <0.001 | 80.2 | 0.002 |
|  | *Without adjunctive* | 1 | 28.90 | 25.47 | 32.33 | <0.001 |  |  |
|  | ***TUN*** | 5 | 24.34 | 16.46 | 32.22 | <0.001 | 74.5 | 0.003 |
| **eprf_res** | ***Overall*** | 43 | 8.31 | 8.11 | 8.50 | <0.001 | 93.2 | <0.001 |
|  | ***CAF*** | 22 | 8.03 | 7.78 | 8.27 | <0.001 | 89.9 | <0.001 |
|  | *With adjunctive* | 19 | 8.08 | 7.82 | 8.34 | <0.001 | 90.1 | <0.001 |
|  | *Without adjunctive* | 3 | 7.62 | 7.29 | 7.96 | <0.001 | 22.8 | 0.274 |
|  | ***TUN*** | 18 | 8.53 | 8.23 | 8.83 | <0.001 | 89.9 | <0.001 |
|  | ***VISTA*** | 1 | 8.82 | 8.29 | 9.35 | <0.001 |  |  |
|  | ***PST*** | 2 | 9.05 | 8.38 | 9.72 | <0.001 | 90.1 | 0.001 |
| **eptf_vas** | ***Overall*** | 32 | 8.59 | 8.29 | 8.89 | <0.001 | 93.9 | <0.001 |
|  | ***CAF*** | 17 | 8.52 | 80.2 | 9.02 | <0.001 | 96.5 | <0.001 |
|  | *With adjunctive* | 13 | 8.58 | 8.03 | 9.13 | <0.001 | 96.3 | <0.001 |
|  | *Without adjunctive* | 4 | 8.33 | 7.24 | 9.41 | <0.001 | 94.3 | <0.001 |
|  | ***TUN*** | 13 | 8.61 | 8.35 | 8.88 | <0.001 | 74.9 | <0.001 |
|  | ***VISTA*** | 2 | 9.05 | 8.69 | 9.42 | <0.001 | 0.0 | 0.557 |

CAF: coronally advanced flap; TUN: tunnel; VISTA: Vista; PST: Pinhole surgical technique; ATG: autogenous graft (any type of technique); CM: collagen membrane; ADM: allodermic membrane; BA: biologic agents (enamel matrix derivative, platelet rich fibrin, chorion membrane, hyaluronic acid, growth factors, recombinant human factors); FGG: free gingival graft; OB: orthodontic application; GS: gingival substitute; ADJ: adjunctives; RT: recession type; CTG: connective tissue graft; CRC: complete root coverage, Rec: recession; VAS: visual analogue scale; eprf: esthetic professional evaluation; eptf: esthetic patient evaluation

Figure S1. Forest plot from random effects of a meta-analysis evaluating the difference in percentage of mean root coverage among techniques [weight mean difference. 95% confidence interval (CI)]. CAF= coronally advanced flap; TUN= tunnel; Vista=Vista; PST=Pinhole surgical technique

CAF: coronally advanced flap; TUN: tunnel; VISTA: Vista; PST: Pinhole surgical technique; ATG: autogenous graft (any type of technique); CM: collagen membrane; ADM: allodermic membrane; BA: biologic agents (enamel matrix derivative, platelet rich fibrin, chorion membrane, hyaluronic acid, growth factors, recombinant human factors); FGG: free gingival graft; OB: orthodontic application; GS: gingival substitute; ADJ: adjunctives; RT: recession type; CTG: connective tissue graft;

Figure S2. Forest plot from random effects of a meta-analysis evaluating the difference in recession reduction among techniques [weight mean difference. 95% confidence interval (CI)]. CAF= coronally advanced flap; TUN= tunnel; Vista=Vista; PST=Pinhole surgical technique

CAF: coronally advanced flap; TUN: tunnel; VISTA: Vista; PST: Pinhole surgical technique; ATG: autogenous graft (any type of technique); CM: collagen membrane; ADM: allodermic membrane; BA: biologic agents (enamel matrix derivative, platelet rich fibrin, chorion membrane, hyaluronic acid, growth factors, recombinant human factors); FGG: free gingival graft; OB: orthodontic application; GS: gingival substitute; ADJ: adjunctives; RT: recession type; CTG: connective tissue graft;

**Figure S3.** Forest plot from random effects of a meta-analysis evaluating the difference in keratinized tissue change among techniques [weight mean difference. 95% confidence interval (CI)]. CAF= coronally advanced flap; TUN= tunnel; Vista=Vista; PST=Pinhole surgical technique

CAF: coronally advanced flap; TUN: tunnel; VISTA: Vista; PST: Pinhole surgical technique; ATG: autogenous graft (any type of technique); CM: collagen membrane; ADM: allodermic membrane; BA: biologic agents (enamel matrix derivative, platelet rich fibrin, chorion membrane, hyaluronic acid, growth factors, recombinant human factors); FGG: free gingival graft; OB: orthodontic application; GS: gingival substitute; ADJ: adjunctives; RT: recession type; CTG: connective tissue graft;

**Figure S4.** Forest plot from random effects of a meta-analysis evaluating the difference in gingival thickness change among techniques [weight mean difference. 95% confidence interval (CI)]. CAF= coronally advanced flap; TUN= tunnel; Vista=Vista; PST=Pinhole surgical technique

CAF: coronally advanced flap; TUN: tunnel; VISTA: Vista; PST: Pinhole surgical technique; ATG: autogenous graft (any type of technique); CM: collagen membrane; ADM: allodermic membrane; BA: biologic agents (enamel matrix derivative, platelet rich fibrin, chorion membrane, hyaluronic acid, growth factors, recombinant human factors); FGG: free gingival graft; OB: orthodontic application; GS: gingival substitute; ADJ: adjunctives; RT: recession type; CTG: connective tissue graft;

**Figure S5.** Forest plot from random effects of a meta-analysis evaluating the difference in clinical attachment level change among techniques [weight mean difference. 95% confidence interval (CI)]. CAF= coronally advanced flap; TUN= tunnel; Vista=Vista; PST=Pinhole surgical technique

CAF: coronally advanced flap; TUN: tunnel; VISTA: Vista; PST: Pinhole surgical technique; ATG: autogenous graft (any type of technique); CM: collagen membrane; ADM: allodermic membrane; BA: biologic agents (enamel matrix derivative, platelet rich fibrin, chorion membrane, hyaluronic acid, growth factors, recombinant human factors); FGG: free gingival graft; OB: orthodontic application; GS: gingival substitute; ADJ: adjunctives; RT: recession type; CTG: connective tissue graft;

**Figure S6.** Forest plot from random effects of a meta-analysis evaluating the difference in postoperative pain among techniques through VAS 0-10 (a) and VAS 0-100 (b)[weight mean difference. 95% confidence interval (CI)]. CAF= coronally advanced flap; TUN= tunnel; Vista=Vista; PST=Pinhole surgical technique

a)

CAF: coronally advanced flap; TUN: tunnel; VISTA: Vista; PST: Pinhole surgical technique; ATG: autogenous graft (any type of technique); CM: collagen membrane; ADM: allodermic membrane; BA: biologic agents (enamel matrix derivative, platelet rich fibrin, chorion membrane, hyaluronic acid, growth factors, recombinant human factors); FGG: free gingival graft; OB: orthodontic application; GS: gingival substitute; ADJ: adjunctives; RT: recession type; CTG: connective tissue graft;

b)

CAF: coronally advanced flap; TUN: tunnel; VISTA: Vista; PST: Pinhole surgical technique; ATG: autogenous graft (any type of technique); CM: collagen membrane; ADM: allodermic membrane; BA: biologic agents (enamel matrix derivative, platelet rich fibrin, chorion membrane, hyaluronic acid, growth factors, recombinant human factors); FGG: free gingival graft; OB: orthodontic application; GS: gingival substitute; ADJ: adjunctives; RT: recession type; CTG: connective tissue graft;

REFERENCES

1. Lakshmi MR, Gottumukkala S, Penmetsa GS, et al. Clinical outcomes of root coverage using porcine-derived collagen matrix with modified coronally advanced tunnel technique (MCAT) in multiple gingival recessions in smokers - a randomized controlled clinical trial. *Clin Oral Investig*. 2023;27:1101-1111.

2. Sterne JAC, Savovic J, Page MJ, et al. RoB 2: a revised tool for assessing risk of bias in randomised trials. *BMJ*. 2019;366:l4898.

3. Moola S. Risky business 'mis'interpretation of observational evidence. *JBI Database System Rev Implement Rep*. 2017;15:1-2.

4. Carcuac O, Trullenque-Eriksson A,Derks J. Modified free gingival graft technique for treatment of gingival recession defects at mandibular incisors: A randomized clinical trial. *J Periodontol*. 2023;94:722-730.

5. Stahli A, Duong HY, Imber JC, et al. Recession coverage using the modified coronally advanced tunnel and connective tissue graft with or without enamel matrix derivative: 5-year results of a randomised clinical trial. *Clin Oral Investig*. 2023;27:105-113.

6. Mercado F, Hamlet S,Ivanovski S. A 3-year prospective clinical and patient-centered trial on subepithelial connective tissue graft with or without enamel matrix derivative in Class I-II Miller recessions. *J Periodontal Res*. 2020;55:296-306.

7. Bommala M, Koduganti RR, Panthula VR, et al. Efficacy of root coverage with the use of the conventional versus laser-assisted flap technique with platelet-rich fibrin in class I and class II gingival recession: A randomized clinical trial. *Dent Med Probl*. 2023;60:583-592.

8. Peter TG, Tadepalli A, Parthasarathy H, et al. Comparison of the Clinical Efficacy of Bioresorbable Xenogeneic Peritoneal Membrane vs Connective Tissue Graft in the Management of Gingival Recession Defects: A Randomized Controlled Clinical Trial. *Int J Periodontics Restorative Dent*. 2023:s244-s254.

9. George SG, Kanakamedala AK, Mahendra J, et al. Treatment of gingival recession using a coronally-advanced flap procedure with or without placental membrane. *J Investig Clin Dent*. 2018;9:e12340.

10. Milinkovic I, Aleksic Z, Jankovic S, et al. Clinical application of autologous fibroblast cell culture in gingival recession treatment. *J Periodontal Res*. 2015;50:363-370.

11. Roman A, Soanca A, Kasaj A, et al. Subepithelial connective tissue graft with or without enamel matrix derivative for the treatment of Miller class I and II gingival recessions: a controlled randomized clinical trial. *J Periodontal Res*. 2013;48:563-572.

12. Suzuki KT, de Jesus Hernandez Martinez C, Suemi MI, et al. Root coverage using coronally advanced flap with porcine-derived acellular dermal matrix or subepithelial connective tissue graft: a randomized controlled clinical trial. *Clin Oral Investig*. 2020;24:4077-4087.

13. McGuire MK, Scheyer ET, Lipton DI, et al. Randomized, controlled, clinical trial to evaluate a xenogeneic collagen matrix as an alternative to free gingival grafting for oral soft tissue augmentation: A 6- to 8-year follow-up. *J Periodontol*. 2021;92:1088-1095.

14. Parlak HM, Yilmaz BT, Durmaz MH, et al. The effects of vertically coronally advanced flap and free gingival graft techniques on shallow vestibule: a randomized comparative prospective trial. *Clin Oral Investig*. 2023;27:7425-7436.

15. Zucchelli G, Mele M, Stefanini M, et al. Patient morbidity and root coverage outcome after subepithelial connective tissue and de-epithelialized grafts: a comparative randomized-controlled clinical trial. *J Clin Periodontol*. 2010;37:728-738.

16. Elena RD, Miren VF, Ana-Maria GD, et al. Analysis of the treatment of RT2 recessions with a xenogeneic collagen matrix vs. connective tissue graft combined with a coronally advanced flap. A double-blinded randomized clinical trial. *Clin Oral Investig*. 2024;28:215.

17. Zuhr O, Rebele SF, Schneider D, et al. Tunnel technique with connective tissue graft versus coronally advanced flap with enamel matrix derivative for root coverage: a RCT using 3D digital measuring methods. Part I. Clinical and patient-centred outcomes. *J Clin Periodontol*. 2014;41:582-592.

18. Nickles K, Ratka-Kruger P, Neukranz E, et al. Ten-year results after connective tissue grafts and guided tissue regeneration for root coverage. *J Periodontol*. 2010;81:827-836.

19. Cardaropoli D,Cardaropoli G. Healing of gingival recessions using a collagen membrane with a hemineralized xenograft: a randomized controlled clinical trial. *Int J Periodontics Restorative Dent*. 2009;29:59-67.

20. Boltchi FE, Allen EP,Hallmon WW. The use of a bioabsorbable barrier for regenerative management of marginal tissue recession. I. Report of 100 consecutively treated teeth. *J Periodontol*. 2000;71:1641-1653.

21. Damante CA, Karam P, Ferreira R, et al. Root surface demineralization by citric acid/tetracycline gel and aPDT associated to subepithelial connective tissue graft improves root coverage outcomes. A 12-month preliminary randomized clinical trial. *J Photochem Photobiol B*. 2019;197:111528.

22. Nizam N, Bengisu O,Sonmez S. Micro- and macrosurgical techniques in the coverage of gingival recession using connective tissue graft: 2 years follow-up. *J Esthet Restor Dent*. 2015;27:71-83.

23. Ucak Turer O, Ozcan M, Alkaya B, et al. Clinical evaluation of injectable platelet-rich fibrin with connective tissue graft for the treatment of deep gingival recession defects: A controlled randomized clinical trial. *J Clin Periodontol*. 2020;47:72-80.

24. Fernandes-Dias SB, de Marco AC, Santamaria M, Jr., et al. Connective tissue graft associated or not with low laser therapy to treat gingival recession: randomized clinical trial. *J Clin Periodontol*. 2015;42:54-61.

25. Barootchi S, Tavelli L, Di Gianfilippo R, et al. Gingival Phenotype Modification as a Result of Root Coverage Procedure with Two Human Dermal Matrices: Long-Term Assessment of a Randomized Clinical Trial. *Int J Periodontics Restorative Dent*. 2021;41:719-726.

26. Pradhan S, Shetty N,Kamath D. Title-comparison of coronally advanced flap with chorion membrane vs coronally advanced flap with connective tissue graft in the treatment of multiple gingival recessions: a split-mouth randomised controlled study. *F1000Res*. 2022;11:533.

27. Ongoz Dede F, Bozkurt Dogan S, Celen K, et al. Comparison of the clinical efficacy of concentrated growth factor and advanced platelet-rich fibrin in the treatment of type I multiple gingival recessions: a controlled randomized clinical trial. *Clin Oral Investig*. 2023;27:645-657.

28. Cevallos CAR, de Resende DRB, Damante CA, et al. Free gingival graft and acellular dermal matrix for gingival augmentation: a 15-year clinical study. *Clin Oral Investig*. 2020;24:1197-1203.

29. Naziker Y,Ertugrul AS. Aesthetic evaluation of free gingival graft applied by partial de-epithelialization and free gingival graft applied by conventional method: a randomized controlled clinical study. *Clin Oral Investig*. 2023;27:4029-4038.

30. Gonzalez-Febles J, Romandini M, Laciar-Oudshoorn F, et al. Tunnel vs. coronally advanced flap in combination with a connective tissue graft for the treatment of multiple gingival recessions: a multi-center randomized clinical trial. *Clin Oral Investig*. 2023;27:3627-3638.

31. Zangrando MSR, Eustachio RR, de Rezende MLR, et al. Clinical and patient-centered outcomes using two types of subepithelial connective tissue grafts: A split-mouth randomized clinical trial. *J Periodontol*. 2021;92:814-822.

32. Rotundo R, Genzano L, Nieri M, et al. Smile esthetic evaluation of mucogingival reconstructive surgery. *Odontology*. 2021;109:295-302.

33. Rakasevic DL, Milinkovic IZ, Jankovic SM, et al. The use of collagen porcine dermal matrix and connective tissue graft with modified coronally advanced tunnel technique in the treatment of multiple adjacent type I gingival recessions: A randomized, controlled clinical trial. *J Esthet Restor Dent*. 2020;32:681-690.

34. Carrera TMI, Machado LM, Soares MTR, et al. Root coverage with platelet-rich fibrin or connective tissue graft: a split-mouth randomized trial. *Braz Oral Res*. 2023;37:e084.

35. Bakhishov H, Isler SC, Bozyel B, et al. De-epithelialized gingival graft versus subepithelial connective tissue graft in the treatment of multiple adjacent gingival recessions using the tunnel technique: 1-year results of a randomized clinical trial. *J Clin Periodontol*. 2021;48:970-983.

36. Meza-Mauricio J, Cortez-Gianezzi J, Duarte PM, et al. Comparison between a xenogeneic dermal matrix and connective tissue graft for the treatment of multiple adjacent gingival recessions: a randomized controlled clinical trial. *Clin Oral Investig*. 2021;25:6919-6929.

37. Gorski B, Szerszen M,Kaczynski T. Effect of 24% EDTA root conditioning on the outcome of modified coronally advanced tunnel technique with subepithelial connective tissue graft for the treatment of multiple gingival recessions: a randomized clinical trial. *Clin Oral Investig*. 2022;26:1761-1772.

38. Cieslik-Wegemund M, Wierucka-Mlynarczyk B, Tanasiewicz M, et al. Tunnel Technique With Collagen Matrix Compared With Connective Tissue Graft for Treatment of Periodontal Recession: A Randomized Clinical Trial. *J Periodontol*. 2016;87:1436-1443.

39. Tavelli L, Barootchi S, Rodriguez MV, et al. Recombinant human platelet-derived growth factor improves root coverage of a collagen matrix for multiple adjacent gingival recessions: A triple-blinded, randomized, placebo-controlled trial. *J Clin Periodontol*. 2022;49:1169-1184.

40. Zucchelli G, Mounssif I, Mazzotti C, et al. Coronally advanced flap with and without connective tissue graft for the treatment of multiple gingival recessions: a comparative short- and long-term controlled randomized clinical trial. *J Clin Periodontol*. 2014;41:396-403.

41. Gorski B, Skierska I, Szerszen M, et al. Tunnel technique with cross-linked hyaluronic acid in addition to subepithelial connective tissue graft, compared with connective tissue graft alone, for the treatment of multiple gingival recessions: 6-month outcomes of a randomized clinical trial. *Clin Oral Investig*. 2023;27:2395-2406.

42. Gorski B, Gorska R, Wysokinska-Miszczuk J, et al. Tunnel technique with enamel matrix derivative in addition to subepithelial connective tissue graft compared with connective tissue graft alone for the treatment of multiple gingival recessions: a randomized clinical trial. *Clin Oral Investig*. 2020;24:4475-4486.

43. Zucchelli G, Mele M, Mazzotti C, et al. Coronally advanced flap with and without vertical releasing incisions for the treatment of multiple gingival recessions: a comparative controlled randomized clinical trial. *J Periodontol*. 2009;80:1083-1094.

44. Nahas R, Gondim V, Carvalho CV, et al. Treatment of multiple recessions with collagen matrix versus connective tissue: a randomized clinical trial. *Braz Oral Res*. 2020;33:e123.

45. Kuka S, Ipci SD, Cakar G, et al. Clinical evaluation of coronally advanced flap with or without platelet-rich fibrin for the treatment of multiple gingival recessions. *Clin Oral Investig*. 2018;22:1551-1558.

46. Trivedi DS, Kolte AP, Kolte RA, et al. Comparative evaluation of pinhole surgical technique with and without A-PRF in the treatment of multiple adjacent recession defects: A clinico radiographic study. *J Esthet Restor Dent*. 2024;36:324-334.

47. Ahmedbeyli C, Ipci SD, Cakar G, et al. Clinical evaluation of coronally advanced flap with or without acellular dermal matrix graft on complete defect coverage for the treatment of multiple gingival recessions with thin tissue biotype. *J Clin Periodontol*. 2014;41:303-310.

48. Ozcelik O, Haytac MC,Seydaoglu G. Treatment of multiple gingival recessions using a coronally advanced flap procedure combined with button application. *J Clin Periodontol*. 2011;38:572-580.

49. Pietruska M, Skurska A, Podlewski L, et al. Clinical evaluation of Miller class I and II recessions treatment with the use of modified coronally advanced tunnel technique with either collagen matrix or subepithelial connective tissue graft: A randomized clinical study. *J Clin Periodontol*. 2019;46:86-95.

50. Santamaria MP, Rossato A, Fernanda Ferreira Ferraz L, et al. Multiple gingival recessions associated with non-carious cervical lesions treated by partial restoration and modified coronally advanced flap with either connective tissue graft or xenogeneic acellular dermal matrix: A randomized clinical trial. *J Periodontol*. 2023;94:731-741.

51. Potey AM, Kolte RA, Kolte AP, et al. Coronally advanced flap with and without platelet-rich fibrin in the treatment of multiple adjacent recession defects: A randomized controlled split-mouth trial. *J Indian Soc Periodontol*. 2019;23:436-441.

52. Ahmedbeyli C, Dirikan Ipci S, Cakar G, et al. Coronally advanced flap and envelope type of flap plus acellular dermal matrix graft for the treatment of thin phenotype multiple recession defects. A randomized clinical trial. *J Clin Periodontol*. 2019;46:1024-1029.

53. Chen Z, Zhong J, Xie Y, et al. Does vestibular incision improve the outcomes of vestibular incision subperiosteal tunnel technique: A randomized clinical trial for treatment of multiple adjacent type 1 gingival recession. *J Esthet Restor Dent*. 2023;35:1131-1138.

54. Cairo F, Cortellini P, Pilloni A, et al. Clinical efficacy of coronally advanced flap with or without connective tissue graft for the treatment of multiple adjacent gingival recessions in the aesthetic area: a randomized controlled clinical trial. *J Clin Periodontol*. 2016;43:849-856.

55. Tonetti MS, Cortellini P, Pellegrini G, et al. Xenogenic collagen matrix or autologous connective tissue graft as adjunct to coronally advanced flaps for coverage of multiple adjacent gingival recession: Randomized trial assessing non-inferiority in root coverage and superiority in oral health-related quality of life. *J Clin Periodontol*. 2018;45:78-88.

56. Pelekos G, Lu JZ, Ho DKL, et al. Aesthetic assessment after root coverage of multiple adjacent recessions with coronally advanced flap with adjunctive collagen matrix or connective tissue graft: Randomized clinical trial. *J Clin Periodontol*. 2019;46:564-571.

57. Tonetti MS, Cortellini P, Bonaccini D, et al. Autologous connective tissue graft or xenogenic collagen matrix with coronally advanced flaps for coverage of multiple adjacent gingival recession. 36-month follow-up of a randomized multicentre trial. *J Clin Periodontol*. 2021;48:962-969.

58. Tavelli L, Barootchi S, Nguyen TVN, et al. Efficacy of tunnel technique in the treatment of localized and multiple gingival recessions: A systematic review and meta-analysis. *J Periodontol*. 2018;89:1075-1090.

59. Elmahdi F, Reda A,Hosny M. Evaluation of Subepithelial Connective Tissue Graft Versus Acellular Dermal Matrix with Modified Coronally Advanced Tunnel Technique in Treatment of Multiple Gingival Recessions: A Randomized, Parallel-Design Clinical Trial. *Int J Periodontics Restorative Dent*. 2022;42:741-751.

60. Yilmaz BT, Comerdov E, Kutuk C, et al. Modified coronally advanced tunnel versus epithelialized free gingival graft technique in gingival phenotype modification: a comparative randomized controlled clinical trial. *Clin Oral Investig*. 2022;26:6283-6293.

61. Aroca S, Molnar B, Windisch P, et al. Treatment of multiple adjacent Miller class I and II gingival recessions with a Modified Coronally Advanced Tunnel (MCAT) technique and a collagen matrix or palatal connective tissue graft: a randomized, controlled clinical trial. *J Clin Periodontol*. 2013;40:713-720.

62. Fernandez-Jimenez A, Estefania-Fresco R, Garcia-De-La-Fuente AM, et al. Comparative study of the modified VISTA technique (m-VISTA) versus the coronally advanced flap (CAF) in the treatment of multiple Miller class III/RT2 recessions: a randomized clinical trial. *Clin Oral Investig*. 2023;27:505-517.

63. Rotundo R, Genzano L, Patel D, et al. Adjunctive benefit of a xenogenic collagen matrix associated with coronally advanced flap for the treatment of multiple gingival recessions: A superiority, assessor-blind, randomized clinical trial. *J Clin Periodontol*. 2019;46:1013-1023.

64. Rajeswari SR, Triveni MG, Kumar ABT, et al. Patient-centered comparative outcome analysis of platelet-rich fibrin-reinforced vestibular incision subperiosteal tunnel access technique and Zucchelli's technique. *J Indian Soc Periodontol*. 2021;25:320-329.
